# Learning loss due to school closures during the COVID-19 pandemic

**DOI:** 10.1073/pnas.2022376118

**Published:** 2021-04-07

**Authors:** Per Engzell, Arun Frey, Mark D. Verhagen

**Affiliations:** ^a^Leverhulme Centre for Demographic Science, University of Oxford, Oxford OX1 1JD, United Kingdom;; ^b^Nuffield College, University of Oxford, Oxford OX1 1NF, United Kingdom;; ^c^Swedish Institute for Social Research, Stockholm University, 106 91 Stockholm, Sweden; ^d^Department of Sociology, University of Oxford, Oxford OX1 1JD, United Kingdom

**Keywords:** COVID-19, learning loss, school closures, social inequality, digital divide

## Abstract

Suspension of face-to-face instruction in schools during the COVID-19 pandemic has led to concerns about consequences for students’ learning. So far, data to study this question have been limited. Here we evaluate the effect of school closures on primary school performance using exceptionally rich data from The Netherlands (*n* ≈ 350,000). We use the fact that national examinations took place before and after lockdown and compare progress during this period to the same period in the 3 previous years. The Netherlands underwent only a relatively short lockdown (8 wk) and features an equitable system of school funding and the world’s highest rate of broadband access. Still, our results reveal a learning loss of about 3 percentile points or 0.08 standard deviations. The effect is equivalent to one-fifth of a school year, the same period that schools remained closed. Losses are up to 60% larger among students from less-educated homes, confirming worries about the uneven toll of the pandemic on children and families. Investigating mechanisms, we find that most of the effect reflects the cumulative impact of knowledge learned rather than transitory influences on the day of testing. Results remain robust when balancing on the estimated propensity of treatment and using maximum-entropy weights or with fixed-effects specifications that compare students within the same school and family. The findings imply that students made little or no progress while learning from home and suggest losses even larger in countries with weaker infrastructure or longer school closures.

## Significance

School closures have been a common tool in the battle against COVID-19. Yet, their costs and benefits remain insufficiently known. We use a natural experiment that occurred as national examinations in The Netherlands took place before and after lockdown to evaluate the impact of school closures on students’ learning. The Netherlands is interesting as a “best-case” scenario, with a short lockdown, equitable school funding, and world-leading rates of broadband access. Despite favorable conditions, we find that students made little or no progress while learning from home. Learning loss was most pronounced among students from disadvantaged homes.

The COVID-19 pandemic is transforming society in profound ways, often exacerbating social and economic inequalities in its wake. In an effort to curb its spread, governments around the world have moved to suspend face-to-face teaching in schools, affecting some 95% of the world’s student population—the largest disruption to education in history (1). The United Nations Convention on the Rights of the Child states that governments should provide primary education for all on the basis of equal opportunity (2). To weigh the costs of school closures against public health benefits (3–6), it is crucial to know whether students are learning less in lockdown and whether disadvantaged students do so disproportionately.

Whereas previous research examined the impact of summer recess on learning, or disruptions from events such as extreme weather or teacher strikes (7–12), COVID-19 presents a unique challenge that makes it unclear how to apply past lessons. Concurrent effects on the economy make parents less equipped to provide support, as they struggle with economic uncertainty or demands of working from home (13, 14). The health and mortality risk of the pandemic incurs further psychological costs, as does the toll of social isolation (15, 16). Family violence is projected to rise, putting already vulnerable students at increased risk (17, 18). At the same time, the scope of the pandemic may compel governments and schools to respond more actively than during other disruptive events.

Data on learning loss during lockdown have been slow to emerge. Unlike societal sectors like the economy or the healthcare system, school systems usually do not post data at high-frequency intervals. Schools and teachers have been struggling to adopt online-based solutions for instruction, let alone for assessment and accountability (10, 19). Early data from online learning platforms suggest a drop in coursework completed (20) and an increased dispersion of test scores (21). Survey evidence suggests that children spend considerably less time studying during lockdown, and some (but not all) studies report differences by home background (22–26). More recently, data have emerged from students returning to school (27–29). Our study represents one of the first attempts to quantify learning loss from COVID-19 using externally validated tests, a representative sample, and techniques that allow for causal inference.

## Study Setting

In this study, we present evidence on the pandemic’s effect on student progress in The Netherlands, using a dataset covering 15% of Dutch primary schools throughout the years 2017 to 2020 (*n* ≈ 350,000). The data include biannual test scores in core subjects for students aged 8 to 11 y, as well as student demographics and school characteristics. Hypotheses and analysis protocols for this study were preregistered ( *SI Appendix*, section 4.1). Our main interest is whether learning stalled during lockdown and whether students from less-educated homes were disproportionately affected. In addition, we examine differences by sex, school grade, subject, and prior performance.

The Dutch school system combines centralized and equitable school funding with a high degree of autonomy in school management (30, 31). The country is close to the Organization for Economic Cooperation and Development (OECD) average in school spending and reading performance, but among its top performers in math (32). No other country has higher rates of broadband penetration (33, 34), and efforts were made early in the pandemic to ensure access to home learning devices (35). School closures were short in comparative perspective ( *SI Appendix*, section 1), and the first wave of the pandemic had less of an impact than in other European countries (36, 37). For these reasons, The Netherlands presents a “best-case” scenario, providing a likely lower bound on learning loss elsewhere in Europe and the world. Despite favorable conditions, survey evidence from lockdown indicates high levels of dissatisfaction with remote learning (38) and considerable disparities in help with schoolwork and learning resources (39).

**Fig. 1.**
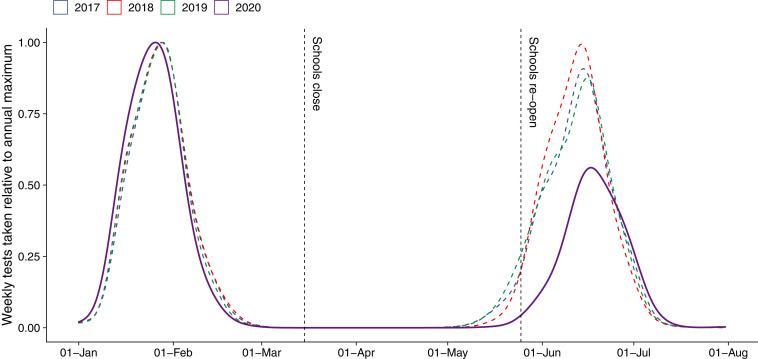
Distribution of testing dates 2017 to 2020 and timeline of 2020 school closures. Density curves show the distribution of testing dates for national standardized assessments in 2020 and the three comparison years 2017 to 2019. Vertical lines show the beginning and end of nationwide school closures in 2020. Schools closed nationally on March 16 and reopened on May 11, after 8 wk of remote learning. Our difference-in-differences design compares learning progress between the two testing dates in 2020 to that in the 3 previous years.

Key to our study design is the fact that national assessments take place twice a year in The Netherlands (40): halfway into the school year in January to February and at the end of the school year in June. In 2020, these testing dates occurred just before and after the first nationwide school closures that lasted 8 wk starting March 16 (Fig. 1). Access to data from 3 y prior to the pandemic allows us to create a natural benchmark against which to assess learning loss. We do so using a difference-in-differences design ( *SI Appendix*, section 4.2) and address loss to follow-up using various techniques: regression adjustment, rebalancing on propensity scores and maximum-entropy weights, and fixed-effects designs that compare students within the same schools and families.

## Results

**Fig. 2.**
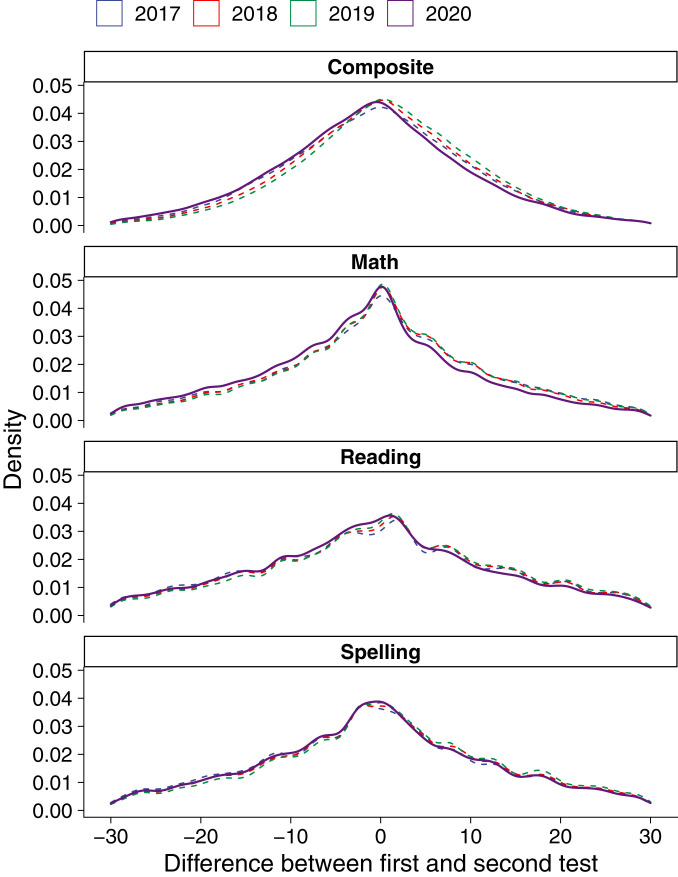
Difference in test scores 2017 to 2020. Density curves show the difference between students’ percentile placement between the first and the second test in each of the years 2017 to 2020. Note that this graph does not adjust for confounding due to trends, testing date, or sample composition, which we address in subsequent analyses using a variety of techniques.

We assess standardized tests in math, spelling, and reading for students aged 8 to 11 y (Dutch school grades 4 to 7) and a composite score of all three subjects. Results are transformed into percentiles by imposing a uniform distribution separately by subject, grade, and testing occasion: midyear vs. end of year. Fig. 2 shows the difference between students’ percentile placement in the midyear and end-of-year tests for each of the years 2017 to 2020. This graph reveals a raw difference ranging from −0.76 percentiles in spelling to −2.15 percentiles in math. However, this difference does not adjust for confounding due to trends, testing date, or sample composition. To address these factors, and assess group differences in learning loss, we go on to estimate a difference-in-differences model ( *SI Appendix*, section 4.2). In our baseline specification, we adjust for a linear trend in year and the time elapsed between testing dates and cluster standard errors at the school level.

### Baseline Specification.

**Fig. 3.**
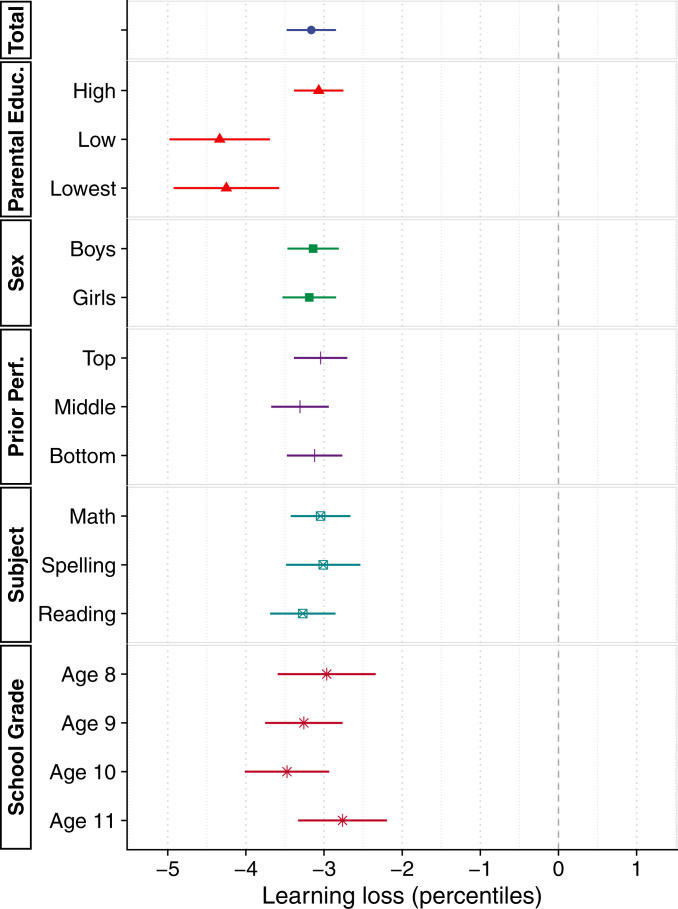
Estimates of learning loss for the whole sample and by subgroup and test. The graph shows estimates of learning loss from a difference-in-differences specification that compares learning progress between the two testing dates in 2020 to that in the 3 previous years. Statistical controls include time elapsed between testing dates and a linear trend in year. Point estimates are with 95% confidence intervals, with robust standard errors accounting for clustering at the school level. One percentile point corresponds to ∼ 0.025 SD. Where not otherwise noted, effects refer to a composite score of math, spelling, and reading. Regression tables underlying these results can be found in *SI Appendix*, section 7.1.

Fig. 3 shows our baseline estimate of learning loss in 2020 compared to the 3 previous years, using a composite score of students’ performance in math, spelling, and reading. Students lost on average 3.16 percentile points in the national distribution, equivalent to 0.08 standard deviations (SD) ( *SI Appendix*, section 4.3). Losses are not distributed equally but concentrated among students from less-educated homes. Those in the two lowest categories of parental education—together accounting for 8% of the population ( *SI Appendix*, section 5.1)—suffered losses 40% larger than the average student (estimates by parental education: high, −3.07; low, −4.34; lowest, −4.25). In contrast, we find little evidence that the effect differs by sex, school grade, subject, or prior performance. In *SI Appendix*, section 7.9, we document considerable variation by school, with some schools seeing a learning slide of 10 percentile points or more and others recording no losses or even small gains.

### Placebo Analysis and Year Exclusions.

In *SI Appendix*, sections 7.2 and 7.3, we examine the assumptions of our identification strategy in several ways. To confirm that our baseline specification is not prone to false positives, we perform a placebo analysis assigning treatment status to each of the 3 comparison years ( *SI Appendix*, section 7.2). In each case, the 95% confidence interval of our main effect spans zero. We also reestimate our main specification dropping comparison years one at a time ( *SI Appendix*, section 7.3). These results are estimated with less precision but otherwise in line with those of our main analysis. In *SI Appendix*, section 7.13, we report placebo analyses for a wider range of specifications than reported in the main text and confirm that our preferred specification fares better than reasonable alternatives in avoiding spurious results.

### Adjusting for Loss to Follow-up.

**Fig. 4.**
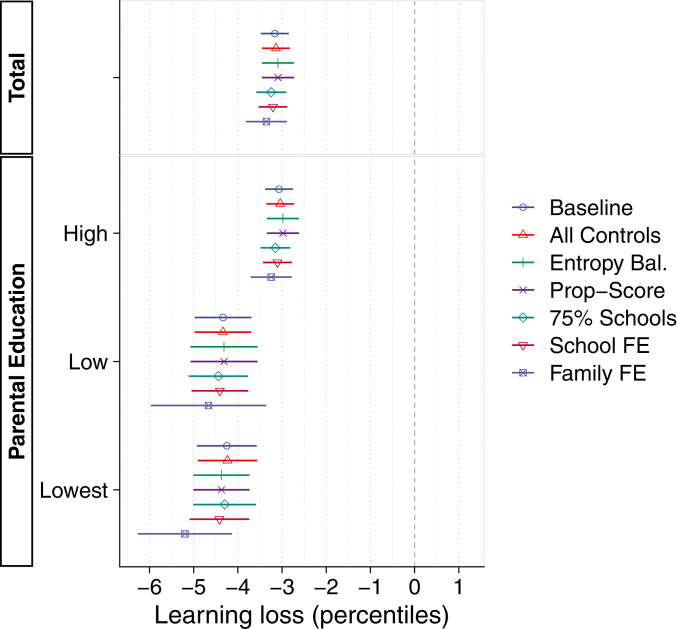
Robustness to specification. The graph shows estimates of learning loss for the whole sample and separately by parental education, using a variety of adjustments for loss to follow-up. Point estimates are with 95% confidence intervals, with robust standard errors accounting for clustering at the school level. For details, see *Materials and Methods* and *SI Appendix*, sections 4.2 and 7.4–7.8.

In Fig. 4, we report a series of additional specifications addressing the fact that only a subset of students returning after lockdown took the tests. Our difference-in-differences design discards those students who did not take the tests, which might lead to bias if their performance trajectories differ from those we observe. In *SI Appendix*, Table S3, we show that the treatment sample is not skewed on sex, parental education, or prior performance. Therefore, adjusting for these covariates makes little difference to our results ( *SI Appendix*, section 7.1). Next, we balance treatment and control groups on a wider set of covariates, including at the school level, using maximum-entropy weights and the estimated propensity of treatment ( *SI Appendix*, section 7.4). Moreover, we restrict analysis to schools where at least 75% of students took their tests after lockdown ( *SI Appendix*, section 7.5). Finally, we adjust for time-invariant confounding at the school and family level using fixed-effects models ( *SI Appendix*, sections 7.6 and 7.7). As Fig. 4 shows, social inequalities grow somewhat when adjusting for selection at the school and family level. The largest gap in effect sizes between educational backgrounds is found in our within-family analysis, estimated at 60% (parental education: high, −3.25; low, −4.67; lowest, −5.20). However, the fixed-effects specification shifts the sample toward larger families, and effects in this subsample are similar using our baseline specification ( *SI Appendix*, section 7.7).

### Knowledge Learned vs. Transitory Influences.

**Fig. 5.**
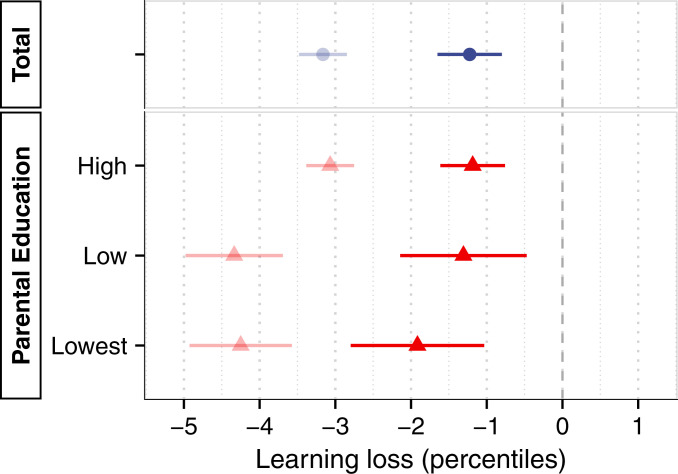
Knowledge learned vs. transitory influences. The graph compares estimates for the composite achievement score in our main analysis (light color) with test not designed to assess curricular content (dark color). Both sets of estimates refer to our baseline specification reported in Fig. 3. Point estimates are with 95% confidence intervals, with robust standard errors accounting for clustering at the school level. For details, see *Materials and Methods* and *SI Appendix*, sections 3.1 and 7.8.

Do these results actually reflect a decrease in knowledge learned or more transient “day of exam” effects? Social distancing measures may have altered factors such as seating arrangements or indoor climate that in turn can influence student performance (41–43). Following school reopenings, tests were taken in person under normal conditions and with minimal social distancing. Still, students may have been under stress or simply unaccustomed to the school setting after several weeks at home. Similarly, if remote teaching covered the requisite material but put less emphasis on test-taking skills, results may have declined while knowledge remained stable. We address this by inspecting performance on generic tests of learning readiness ( *SI Appendix*, section 3.1). These tests present the student with a series of words to be read aloud within a given time. Understanding of the words is not needed, and no curricular content is covered. The results, in Fig. 5, show that the treatment effects shrink by nearly two-thirds compared to our main outcome (main effect −1.19 vs. −3.16), suggesting that differences in knowledge learned account for the majority of the drop in performance. In years prior to the pandemic, we observe no such difference in students’ performance between the two types of test ( *SI Appendix*, section 7.8).

### Specification Curve Analysis.

To identify the model components that exert the most influence on the magnitude of estimates we assessed more than 2,000 alternative models in a specification curve analysis (44) ( *SI Appendix*, section 7.13). Doing so identifies the control for pretreatment trends as the most influential, followed by the control for test timing and the inclusion of school and family fixed effects. Disregarding the trend and instead assuming a counterfactual where achievement had stayed flat between 2019 and 2020, the estimated treatment effect shrinks by 21% to −2.51 percentiles ( *SI Appendix*, section 7.11). However, failure to adjust for pretreatment trends generates placebo estimates that are biased in a positive direction and is thus likely to underestimate treatment effects. Excluding adjustment for testing date decreases the effect size by 12%, while including fixed effects increases it by 1.6% (school level) or 6.3% (family level). The placebo estimate closest to zero is found in the version of our preferred specification that includes family fixed effects. The specification curve also reveals that treatment effects in math are more invariant to assumptions than those in either reading or spelling.

## Discussion

During the pandemic-induced lockdown in 2020, schools in many countries were forced to close for extended periods. It is of great policy interest to know whether students are able to have their educational needs met under these circumstances and to identify groups at special risk. In this study, we have addressed this question with uniquely rich data on primary school students in The Netherlands. There is clear evidence that students are learning less during lockdown than in a typical year. These losses are evident throughout the age range we study and across all of the three subject areas: math, spelling, and reading. The size of these effects is on the order of 3 percentile points or 0.08 SD, but students from disadvantaged homes are disproportionately affected. Among less-educated households, the size of the learning slide is up to 60% larger than in the general population.

Are these losses large or small? One way to anchor these effects is as a proportion of gains made in a normal year. Typical estimates of yearly progress for primary school range between 0.30 and 0.60 SD (45). In their projections of learning loss due to the pandemic, the World Bank assumes a yearly progress of 0.40 SD (46). We validate these benchmarks in our data by exploiting variation in testing dates during comparison years and show that test scores improve by 0.30 to 0.40 percentiles per week, equivalent to 0.31 to 0.41 SD annually ( *SI Appendix*, section 4.3). Using the larger benchmark, a treatment effect of 3.16 percentiles would translate into 3.16/0.40 = 7.9 wk of lost learning—nearly exactly the same period that schools in The Netherlands remained closed. Using the smaller benchmark, learning loss exceeds the period of school closures (3.16/0.30 = 10.5 wk), implying that students regressed during this time. At the same time, some studies indicate a progress of up to 0.80 SD annually at the low extreme of our age range (45, 47), which would indicate that remote learning operated at 50% efficiency.

Another relevant source of comparison is studies of how students progress when school is out of session for summer (7–10). This literature reports reductions in achievement ranging from 0.001 to 0.010 SD per school day lost (10). Our estimated treatment effect translates into 3.16/35 = 0.09 percentiles or 0.002 SD per school day and is thus on the lower end of that range.* Although early influential studies also found that summer is a time when socioeconomic learning gaps widen, this finding has failed to replicate in more recent studies (8, 9) or in European samples (48, 49). However, there are limits to the analogy between summer recess and forced school closures, when children are still being expected to learn at a normal pace (50). Our results show that learning loss was particularly pronounced for students from disadvantaged homes, confirming the fears held by many that school closures would cause socioeconomic gaps to widen (51–55).

We have described The Netherlands as a best-case scenario due to the country’s short school closures, high degree of technological preparedness, and equitable school funding. However, this does not mean that circumstances were ideal. The short duration of school closures gave students, educators, and parents little time to adapt. It is possible that remote learning might improve with time (47). At the very least, our results imply that technological access is not itself sufficient to guarantee high-quality remote instruction. The high degree of school autonomy in The Netherlands is also likely to have created considerable variation in the pandemic response, possibly explaining the wide school-level variation in estimated learning loss ( *SI Appendix*, section 7.9).

Are these results a temporary setback that schools and teachers can eventually compensate? Only time will tell whether students rebound, remain stable, or fall farther behind. Dynamic models of learning stress how small losses can accumulate into large disadvantages with time (56–58). Studies of school days lost due to other causes are mixed—some find durable effects and spillovers to adult earnings (59, 60), while others report a fadeout of effects over time (61, 62). If learning losses are transient and concentrated in the initial phase of the pandemic, this could explain why results from the United States appear less dramatic than first feared. Early estimates suggest that grades 3 to 8 students more than 6 mo into the pandemic underperformed by 7.5 percentile points in math but saw no loss in reading achievement (28).

Nevertheless, the magnitude of our findings appears to validate scenarios projected by bodies such as the European Commission (34) and the World Bank (46).^†^ This is alarming in light of the much larger losses projected in countries less prepared for the pandemic. Moreover, our results may underestimate the full costs of school closures even in the context that we study. Test scores do not consider children’s psychosocial development (63, 64), either societal costs due to productivity decline or heightened pressure among parents (65, 66). Overall, our results highlight the importance of social investment strategies to “build back better” and enhance resilience and equity in education. Further research is needed to assess the success of such initiatives and address the long-term fallout of the pandemic for student learning and wellbeing.

## Materials and Methods

Three features of the Dutch education system make this study possible ( *SI Appendix*, section 2). The first one is the student monitoring system, which provides our test score data (40). This system comprises a series of mandatory tests that are taken twice a year throughout a child’s primary school education (ages 6 to 12 y). The second one is the weighted system for school funding, which until recently obliged schools to collect information on the family background of all students (31). Third is the fact that some schools rely on third-party service providers to curate data and provide analytical insights. It is not uncommon that such providers generate anonymized datasets for research purposes. We partnered with the Mirror Foundation (https://www.mirrorfoundation.org/), an independent research foundation associated with one such service provider, who gave us access to a fully anonymized dataset of students’ test scores. The sample covers 15% of all primary schools and is broadly representative of the national student body ( *SI Appendix*, section 5.1).

### Test Scores.

Nationally standardized tests are taken across three main subjects: math, spelling, and reading ( *SI Appendix*, section 3.1). Students across The Netherlands take the same examination within a given year. These tests are administered in school, and each of them lasts up to 60 min. Test results are transformed to percentile scores, but the norm for transformation is the same across years so absolute changes in performance over time are preserved. We rely on translation keys provided by the test producer to assign percentile scores. However, as these keys are actually based on smaller samples than that at our disposal, we further impose a uniform distribution in our sample within cells defined by subject, grade, and testing occasion: midyear vs. end of year.

Our main outcome is a composite score that takes the average of all nonmissing values in the three areas (math, spelling, and reading). In sensitivity analyses in *SI Appendix*, section 7.1, we require a student to have a valid score on all three subjects. We also display separate results for the three subtests in Fig. 3. The test in math contains both abstract problems and contextual problems that describe a concrete task. The test in reading assesses the student’s ability to understand written texts, including both factual and literary content. The test in spelling asks the student to write down a series of words, demonstrating that the student has mastered the spelling rules. Reliability on these tests is excellent: Composite achievement scores correlate above 0.80 for an individual across 2 study years ( *SI Appendix*, section 5.3).

As an alternative outcome we also assess students’ performance on shorter assessments of oral reading fluency in Fig. 5 ( *SI Appendix*, section 3.1). This test consists of a set of cards with words of increasing difficulty to be read aloud during an allotted time. In the terminology of the test producer, its goal is to assess “technical reading ability”—likely a mix of reading ability, cognitive processing, and verbal fluency. We interpret it as a test of learning readiness. Crucially, comprehension of the words is not needed and students and parents are discouraged to prepare for it. As this part of the assessment does not test for the retention of curricular content, we would expect it to be less affected by school closures, which is indeed what we find.

### Parental Education.

Data on parental education are collected by schools as part of the national system of weighted student funding, which allocates greater funds per student to schools that serve disadvantaged populations. The variable codes as high educated those households where at least one parent has a degree above lower secondary education, as low educated those where both parents have a degree above primary education but neither has one above lower secondary education, and as lowest educated those where at least one parent has no degree above primary education and neither parent has a degree above lower secondary education. These groups make up, respectively, 92, 4, and 4% of the student body and our sample ( *SI Appendix*, section 5.1). We provide a more extensive discussion of this variable in *SI Appendix*, sections 3.2, 5.3, and 5.4.

### Other Covariates.

Sex is a binary variable distinguishing boys and girls. Prior performance is constructed from all test results in the previous year. We create a composite score similar to our main outcome variable and split this into tertiles of top, middle, and bottom performance. School grade is the year the student belongs in at the time of testing. School starts at age 4 y in The Netherlands but the first three grades are less intensive and more akin to kindergarten. The last grade of comprehensive school is grade 8, but this grade is shorter and does not feature much additional didactic material. In matched analyses using reweighting on the propensity of treatment and maximum-entropy weights, we also include a set of school characteristics described in *SI Appendix*, section 3.2: school-level socioeconomic disadvantage, proportion of non-Western immigrants in the school’s neighborhood, and school denomination.

### Difference-in-Differences Analysis.

We analyze the rate of progress in 2020 to that in previous years using a difference-in-differences design. This first involves taking the difference in educational achievement prelockdown (measured using the midyear test) compared to that postlockdown (measured using the end-of-year test): $\Delta y_{i}^{2020}=y_{i}^{2020-\text{end}}-y_{i}^{2020-\text{mid}}$ , where $y_{i}$ is some achievement measure for student i and the superscript 2020 denotes the treatment year. We then calculate the same difference in the 3 y prior to the pandemic, $\Delta y_{i}^{2017-2019}$ . These differences can then be compared in a regression specification,

$$\Delta y_{i}=\alpha +Z_{i}^{\prime }\gamma +\delta T_{i}+\epsilon_{ij},$$ [1]

where $Z_{i}$ is a vector of control variables, $T_{i}$ is an indicator for the treatment year 2020, and $\epsilon_{ij}$ is an independent and identically distributed error term clustered at the school level. In our baseline specification, $Z_{i}$ includes a linear trend for the year of testing and a variable capturing the number of days between the two tests. To assess heterogeneity in the treatment effect, we add terms interacting each student characteristic $X_{i}$ with the treatment indicator $T_{i}$ ,

$$\Delta y_{i}=\alpha +Z_{i}^{\prime }\gamma +\beta X_{i}+\delta_{0}T_{i}+\delta_{1}T_{i}X_{i}+\epsilon_{ij},$$ [2]

where $X_{i}$ is one of parental education, student sex, or prior performance. In addition, we estimate Eq. **1** separately by grade and subject. In *SI Appendix*, section 3.2, we provide more extensive motivation and description of our model and the additional strategies we use to deal with loss to follow-up. Throughout our analyses, we adjust confidence intervals for clustering on schools using robust standard errors.

### Effect Size Conversion.

Our effect sizes are expressed on the scale of percentiles. In educational research it is common to use standard-deviation–based metrics such as Cohen’s d (67). Assuming that percentiles were drawn from an underlying normal distribution, we use the following formula to convert between one and the other:

$$\text{d}=\Phi^{-1}\left(0.50+\frac{\delta }{100}\right),$$ [3]

where δ is the treatment effect on the percentile scale, and $\Phi^{-1}$ is the inverse cumulative standard normal distribution. Generally, with “small” or “medium” effect sizes in the range d∈[−0.5,0.5] , this transformation implies a conversion factor of about 0.025 SD per percentile.

### Propensity Score and Entropy Weighting.

Moreover, we match treatment and control groups on a wider range of individual- and school-level characteristics using reweighting on the propensity of treatment (68) and maximum-entropy balancing (69). In both cases, we use sex, parental education, prior performance, two- and three-way interactions between them, a student’s school grade, and school-level covariates: school denomination, school disadvantage, and neighborhood ethnic composition. Propensity of treatment weights involves first estimating the probability of treatment using a binary response (logit) model and then reweighting observations so that they are balanced on this propensity across comparison and treatment groups. The entropy balancing procedure instead uses maximum-entropy weights that are calibrated to directly balance comparison and treatment groups nonparametrically on the observed covariates.

### School and Family Fixed Effects.

We perform within-school and within-family analyses using fixed-effects specifications (70). The within-school design discards all variation between schools by introducing a separate intercept for each school. By doing so, it eliminates all unobserved heterogeneity across schools which might have biased our results if, for example, schools where progression within the school year is worse than average are overrepresented during the treatment year. The same logic applies to the within-family design, which discards all variation between families by introducing a separate intercept for each group of siblings identified in our data. This step reduces the size of our sample by approximately 60%, as not every student has a sibling attending a sampled school within the years that we are able to observe.

## Supplementary Material

Supplementary File

## Data Availability

The data underlying this study are confidential and cannot be shared due to ethical and legal constraints. We obtained access through a partnership with a nonprofit who made specific arrangements to allow this research to be done. For other researchers to access the exact same data, they would have to participate in a similar partnership. Equivalent data are, however, in the process of being added to existing datasets widely used for research, such as the Nationaal Cohortonderzoek Onderwijs (NCO). Analysis scripts underlying all results reported in this article are available online at https://github.com/MarkDVerhagen/Learning_Loss_COVID-19.

## Footnotes

*Although the school closure lasted for 8 wk, one of these weeks occurred during Easter, which leaves 7 wk × 5 = 35 effective school days.

^†^The World Bank’s “optimistic” scenario—schools operating at 60% efficiency for 3 mo—projects a 0.06 SD loss in standardized test scores (46). The European Commission posits a lower-bound learning loss of 0.008 SD/wk (34), which multiplied by 8 wk translates to 0.064 SD. Both these scenarios are on the same order of magnitude as our findings if marginally smaller.
