# Supplementary material for: Learning loss due to school closures during the COVID-19 pandemic
Source: Proc Natl Acad Sci U S A. 2021 Apr 7;118(17):e2022376118. doi: 10.1073/pnas.2022376118 (PMC8092566; doi:10.1073/pnas.2022376118)
Supplement: Supplementary File [file pnas.2022376118.sapp.pdf]

Supplementary Information for

Learning loss due to school closures during the COVID-19 pandemic

Per Engzell, Arun Frey, Mark Verhagen

Corresponding author: Per Engzell.
E-mail: per.engzell@nuffield.ox.ac.uk

This PDF file includes:

Supplementary text
Figs. S1 to S21
Tables S1 to S27
References for SI reference citations

Contents

1 Study context 4

2 Data sources 4
2.1 Student monitoring system (LVS) 4
2.2 Student background data 5
2.3 Data partner (Mirror Foundation) 5

3 Variables 5
3.1 Outcomes 5
3.1.1 Curricular tests 5
3.1.2 Learning readiness 5
3.2 Covariates 6
3.2.1 School grade 6
3.2.2 Parental education 6
3.2.3 Prior performance 6
3.2.4 Immigrant background 6
3.2.5 School disadvantage 7
3.2.6 School denomination 7
3.2.7 Sex and sibling identification 7

4 Analytical strategy 7
4.1 Pre-analysis plan 7
4.2 Identification strategy 8
4.3 Effect size conversions 10
4.3.1 Percentiles and standardized effects 10
4.3.2 Benchmarks for annual progress 10
4.4 Statistical software 11

5 Quality control 11
5.1 Representativeness 11
5.2 Missing data 11
5.3 Measurement error 12
5.3.1 Parental education 12
5.3.2 Test scores 12
5.4 Confounding 13

6 Descriptive statistics 13

7 Additional results 13
7.1 Regression tables 13
7.2 Placebo analysis 14
7.3 Year exclusions 14
7.4 Covariate balancing 14
7.5 Near-complete schools 14
7.6 School fixed effects 14
7.7 Family fixed effects 14
7.8 Learning readiness 15
7.9 School variability 15
7.10 Three-way interactions 15
7.11 Trend assumption 15
7.12 Days between tests 15
7.13 Specification curve 16

References 65

List of Figures

S1 School closures in the OECD . . . 17
S2 Representativity of the sample. . . . 18
S3 Trends in student progress . . . 19
S4 Results with complete subject scores . . . 20
S5 Placebo effects for non-treated years . . . 21
S6 Robustness dropping comparison years . . . 22
S7 Balancing plot for weighted comparisons . . . 23
S8 Results with covariate balancing . . . 24
S9 Results for near-complete schools . . . 25
S10 School fixed effects . . . 26
S11 Family fixed effects . . . 27
S12 Results for learning readiness . . . 28
S13 School-level effects . . . 29
S14 School-level effects with controls . . . 30
S15 Robustness to trend assumption . . . 31
S16 Robustness excluding testing date . . . 32
S17 Specification curve: Composite . . . 33
S18 Specification curve: Maths . . . 34
S19 Specification curve: Spelling . . . 35
S20 Specification curve: Reading . . . 36
S21 Specification curve: Placebo . . . 37

List of Tables

S1 Estimated weekly learning gains . . . 38
S2 Percent missing data . . . 39
S3 Summary statistics . . . 40
S4 Correlation between prior and current performance . . . 41
S5 Immigrant status by parental education . . . 42
S6 Performance by test, year, and student characteristics . . . 43
S7 Main effects by subject . . . 44
S8 Results by parental education and subject . . . 45
S9 Results by student sex and subject . . . 46
S10 Results by prior performance and subject . . . 47
S11 Results by grade and subject . . . 48
S12 Main effects with controls . . . 49
S13 Main effects, complete subject scores only . . . 50
S14 Main effects in near-complete schools . . . 51
S15 Social inequality in near-complete schools . . . 52
S16 Main effects with school fixed effects . . . 53
S17 Social inequality with school fixed effects . . . 54
S18 Main effects with sibling fixed effects . . . 55
S19 Social inequality with sibling fixed effects . . . 56
S20 Main effects, OLS in sibling subsample . . . 57
S21 Social inequality, OLS in sibling subsample . . . 58
S22 Social inequality by student sex . . . 59
S23 Social inequality by prior performance . . . 60
S24 Main effects with single comparison year . . . 61
S25 Social inequality with single comparison year . . . 62
S26 Main effects excluding testing date . . . 63
S27 Social inequality excluding testing date . . . 64

Supporting Information Text

1. Study context

Education in the Netherlands is based on a common school up to the age of 12, after which students are placed on separate tracks (1, 2). Schooling is compulsory from age 5 to 16, but the majority of children start at the age of 4 (1). The Dutch system combines a high degree of school autonomy with a centralized system for school funding and accountability (1–3). The system ultimately dates back to the early 20th century, and arose as a compromise to give schools equal access to state funding regardless of denomination (3, 4). To this day, most schools are denominational, predominantly Roman Catholic or Protestant. Schools are run by local school boards, and the right to establish a school is enshrined in the constitution, once certain basic criteria are met (1). All schools are funded by the Ministry of Education, Culture and Science ("Ministry of Education" henceforth), with schools that serve disadvantaged students receiving a larger budget per capita (5). The Dutch system achieves a high degree of both efficiency and equity as measured by performance on international student assessments (2). The Netherlands, while close to the OECD average in school spending and in Reading performance, places among Europe's top performers in Maths (6).

During the first wave of the COVID-19 pandemic, the government pursued a so-called "intelligent lockdown," relying on voluntary cooperation and allowing ordinary life to continue as far as possible (7–9). School closures were one of few strictly enforced non-pharmaceutical interventions. However, their duration were short compared to most other OECD countries. As shown in Fig. S1, schools closed on March 16 and reopened eight weeks later, on May 11. While students initially attended classes every other day, in-person schooling returned to normal activity from June 8. Arguably, the Netherlands was unusually well prepared for remote learning: the country leads the world in broadband penetration (10, 11) with more than 90% of households enjoying broadband access even among the poorest quartile (12). Adding to this advantage, the response of national and local governments was swift: in March 2020, the Ministry of Education devoted 2.5 million euros to online learning devices for students in need (13), and this scheme was extended with another 3.8 million in June (14), with similar initiatives at a local level.

Despite these efforts, anecdotal evidence suggests that primary school teachers had limited prior experience of or preparation for distance learning. In contrast to older students who can be expected to shoulder some of the responsibility for their study themselves, primary school study is more dependent on continuous instruction from a teacher. Being deprived of classroom instruction meant that the responsibility for structuring the school day and creating a supportive work environment largely fell on parents. Many teachers created instruction packets with physical assignments and handouts that parents had to collect from schools. There is limited data on how much instruction actually took place online, and how many hours of effective school work students were able to achieve. However, evidence from Germany suggests that students reduced their study time by as much as half (15). Survey evidence from the Netherlands also indicates that there were considerable disparities in help with schoolwork and learning resources (16), and high levels of dissatisfaction with remote learning (7). This evidence mirrors that from several other countries, showing important disparities in children's conditions for learning from home (17–20).

2. Data sources

Three features of the Dutch education system make this study possible. The first is the student monitoring system, LVS, which provides our test score data. This system comprises a series of mandatory tests that are taken twice a year throughout a child's primary school education (age 6–12). The second feature is the weighted system for school funding, which until recently obliged schools to collect information on the family background of all students. Third is the fact that some schools rely on third-party service providers to curate data and provide analytical insights. It is not uncommon that such providers generate anonymized datasets for research purposes. We teamed up with the Mirror Foundation (https://www.mirrorfoundation.org/), an independent research foundation associated with one such service provider, who gave us access to a fully anonymized dataset of students' test scores. In the following we describe the student monitoring system, the student background data, and our data partner.

2.1. Student monitoring system (LVS). The measures of student performance that we use are gathered from the student monitoring system or *leerlingvolgsysteem* (LVS), which is a distinguishing feature of Dutch primary education (21). The LVS is one of several components introduced to uphold quality and accountability despite the country's high degree of school autonomy (2). Diagnostic tests are administered to all students twice a year, normally in the middle of the school year in January/February and at the end of the school year in June. By continuously assessing students and tracking their performance longitudinally, the system helps educators tailor their instruction to the needs of a particular cohort and identify students in demand of extra support. The LVS was first developed by the National Institute for Educational Measurement, CITO, in the 1990s. CITO was originally founded as a non-profit organization in the 1960s, but is today a commercial enterprise with several international branches. In the Netherlands, CITO

testing services are developed and sold on a "semicommercial" basis (21), which means that the Ministry of Education serves as the main funder of CITO and appoints its board director. Schools decide on whether to purchase their service using education funds that are public. Since 2014, it is mandatory for all primary schools to use an LVS, with CITO being the leading provider holding a large majority of the market share.

2.2. Student background data. Data on student background are collected by schools as part of the national system of weighted student funding or *gewichtenregeling*. Primary education in the Netherlands is operated as a voucher system, where funding is provided to schools by the Ministry of Education on a per-student basis (2, 5). Since 1985, an additional contribution toward each student depends on their social background in an effort to reduce social inequality and raise bottom performance. The amount of funding that a school gets is proportional to the socioeconomic composition of the student body, with schools with a higher proportion of disadvantaged students receiving more funds per student. To support this system, schools are legally required to collect data on parental background when a student first starts school or transfers between schools. The number of indicators used to determine school funding has changed throughout the history of the system, but between 2006 and 2019, parental education was the sole indicator (22, 23). In 2019, responsibility for determining funding weights was transferred to the central government, using a wider set of indicators and administrative data stored by Statistics Netherlands. As this information is only available at a school level, our main analysis uses the individual-level data on parental education collected by schools. We use the newer indicator of student disadvantage derived from administrative data in propensity-score weighted and entropy-balanced analyses in Section 7.4, as well as in school-level analyses in Section 7.9.

2.3. Data partner (Mirror Foundation). To access test scores and student background data, we entered a partnership with the Mirror Foundation (https://www.mirrorfoundation.org/), an independent non-profit research foundation set up to support educational research initiatives. The Mirror Foundation enabled us to access a fully anonymized dataset of 15% of primary schools in the Netherlands. The schools are users of a data analytics platform that provides school boards with timely insights based on LVS and other data that are kept by the schools. All schools in the Netherlands are mandated to use a digital interface for student monitoring, and some also subscribe to services offering more extensive functionality and independent analysis—as is the case for the schools in our sample. The dataset was generated by anonymizing existing school records from the schools' LVS and done at the schools' instruction, whereby the latter act as 'data controller' in the definition of the EU's General Data Protection Regulation (GDPR). Anonymization was done with the explicit and stated objective of supporting academic research. All analysis was carried out in accordance with the GDPR and at no point did the authors have access to data that would allow the identification of individuals. The study received ethical approval from the Central University Research Ethics Committee at the University of Oxford.

3. Variables

3.1. Outcomes.

3.1.1. Curricular tests. Achievement is measured via the LVS system using performance on standardized tests developed by CITO. Tests are taken across three main subject areas: Maths, Spelling, and Reading, the first two of which are mandatory. Each test lasts up to one hour per subject. Maths comprises abstract problems involving the four arithmetic operations—addition, subtraction, multiplication, division—as well as applied problems based on concrete tasks. The applied tasks evaluate the student's facility with concepts such as time or currency. In Spelling, a series of words is presented verbally and the student demonstrates that he or she has mastered the spelling rules by writing the words down correctly. Reading assesses the student's ability to understand written texts, including both factual and literary content. The student is presented with a series of texts and at the end of each, he or she gets to answer a set of multiple-choice questions. All tests are psychometrically validated by CITO and translated to national performance benchmarks expressed as percentile scores for a given grade and test (24–26). However, as the translation keys provided by the test producer are actually based on smaller samples than that at our disposal, we further re-norm the distribution within our sample. That is, we pool results across all study years and impose a uniform distribution separately by subject, grade, and testing occasion: mid-year vs end-of-year. Our main outcome is a composite score that takes the average of non-missing percentile scores across the three subject areas. We also display performance on each separate test and, in supplementary analyses in Section 7.1, require a student to have valid scores in all three subjects. The reliability of these tests is excellent, as we discuss in Section 5.3 below.

3.1.2. Learning readiness. As an alternative outcome we also assess students' performance on a test of learning readiness known as the *drieminutentoets* ("3-minute test") (27). This test is similar in design to the international Test of Word

Reading Efficiency (28). It consists of three cards of which the first two presents 150 monosyllabic words of increasing difficulty, and the third presents 120 words with 2–4 syllables each. The task is to read as many words as possible out loud during an allotted time of one minute per card. A score is calculated by counting the number of successfully read words. These tests are likely to require a range of skills including reading ability, cognitive processing and verbal fluency. Crucially, however, they do not require any comprehension of the words involved and their aim is not to test the retention of curricular content. Unlike the other diagnostic tests, they do not count towards a child's wider school assessment and it is common to advise parents and children against preparing for them. For example, *Ouders van Nu* (Parents Today), a popular magazine and online information portal, writes: "You do not have to prepare your child for the 3-minute test. It is better not to pay too much attention to it, because it can cause your child to fear failure. Moreover, the test can give a distorted picture if your child has practiced at home" (29). While the content is divorced from the taught curriculum, these tests are taken in conjunction with the regular assessments and under similar circumstances. Therefore, if observed learning loss was mainly due to "day of exam" influences such as increased stress, distractions, or unfamiliar testing environments, we would expect similar setbacks in the pandemic year as on other tests. We standardize test scores in the same way as for the other tests, by imposing a uniform distribution within school grade and testing occasion, pooled across all years.

3.2. Covariates.

3.2.1. School grade. Schooling in the Netherlands is mandatory from age 5, but the first three grades feature limited didactic material and are comparable to kindergarten (2). We therefore follow students from grade 4 and until the penultimate grade of primary schooling, grade 7. The final grade 8 is dedicated to transitioning to secondary education and is shorter than the other grades. Also, the standard end-of-year test is in fact the school leaver's test, which was suspended in 2020. The designation of grades differs from international standards, where the ages we study would correspond to grades 1–4 of elementary school. To avoid confusion with international standards we choose to label grades by the modal age of students in each grade. Given that tests are taken in the latter half of the school year, these are ages 8, 9, 10, and 11.

3.2.2. Parental education. Information on parental education is collected from parents by schools as part of the weighted student funding system (5). The classification is therefore the one designated by the Ministry of Education to determine school funding weights. The variable takes on three values: *high* if at least one parent has a degree above lower secondary education; *low* if both parents have a degree above primary education but neither has one above lower secondary; and *lowest* if at least one parent has no degree above primary education and neither has a degree above lower secondary. The three groups make up, respectively, 92%, 4%, and 4% of the student body and our sample (Fig. S2). The school funding weights based on surveys of parental education were replaced by a new system based on administrative data in 2019 (Section 2.2). Nevertheless, the earlier information collected by schools remains available and we rely on it in our main analysis for two reasons. First, the new funding weights are only made available at a school level and therefore do not allow us to distinguish the socioeconomic background of individual students. Secondly, survey data on education are likely to be superior in some respects, especially for immigrant parents whose credentials often do not register in official statistics. We provide further discussion on the strengths and weaknesses of this variable in Section 5.3 and 5.4 below.

3.2.3. Prior performance. To assess prior performance, we take all available tests from the previous year and calculate a percentile score similarly to our main outcome measures. We then create a categorical variable by calculating a student's average rank across all non-missing values and splitting the variable into three equal-sized groups. By basing this information on data collected in the previous year, we avoid the mechanical correlation that would obtain if prior performance had been measured at baseline in the same year as we assess student progress. Doing so is known to introduce regression to the mean which can lead to various statistical artifacts (30). In Section 5.3 and 6, we show that prior performance is the strongest predictor of current performance. In Section 5.3, we show that our strategy of measuring prior performance in the previous year successfully breaks the mechanical correlation with subsequent achievement trajectories.

3.2.4. Immigrant background. In the Netherlands today, immigrant minorities make up a significant share of the student body (2). Unfortunately we lack an individual-level indicator of immigrant background. Instead, we measure the proportion of non-Western inhabitants in a school's neighborhood using administrative data. A person is defined as having a non-Western background if they or at least one of their parents were born in Turkey or countries in Africa, Latin America and Asia, except former Dutch colonies and Japan. Although this measure reflects the composition of the neighborhood rather than the student body, the two are likely to be correlated given that residential proximity is one of the most important determinants of school choice in the Netherlands (31, 32). We use this measure in

propensity-score weighted and entropy-balanced analyses in Section 7.4, as well as in school-level analyses in Section 7.9. The absence of an individual-level measure of immigrant background raises the possibility that differential effects of the pandemic by parental education may be driven by immigrant minorities. We discuss this issue and why we do not believe it is a grave concern in Section 5.4 below.

3.2.5. School disadvantage. In recent years, there has been increasing debate about the reliance on parental education as the sole indicator of socioeconomic disadvantage and determinant of school funding weights in the Dutch system (22, 23). Following a prolonged investigation, the practice was therefore replaced in 2019 by one where the Ministry of Education determines school funding with the help of administrative data held by Statistics Netherlands (33). The factors considered in the new measure include the educational level of both the mother and the father as before; but also the country of origin of the parents, the duration of the mother's residence in the Netherlands, and whether parents have taken part in debt restructuring (*schuldsanering*) (34). We prefer the earlier parental education measure as it is available at an individual level. However, we use the new school funding weights in propensity-score weighted and entropy-balanced analyses in Section 7.4, as well as in school-level analyses in Section 7.9.

3.2.6. School denomination. A main policy justification for the high degree of decentralization in Dutch education is the notion that parents should have the right to choose a school for their children that corresponds to their values (1, 3, 4). Consequently, the majority of schools are run by private school boards, and a large proportion of them are faith-based (mostly Christian). We therefore include school denomination as an additional adjustment variable in our matched analyses. Here we distinguish between three categories: public schools, Christian schools, and other schools. Christian schools include schools of Protestant, Catholic, and Reformist denomination. The "other" category includes faith-based schools of non-Christian denomination (e.g., Jewish, Muslim, Hindu), but is predominantly made up of schools based on a particular pedagogy, such as Freinet, Montessori, Pestalozzi, Reggio Emilia, or Waldorf education.

3.2.7. Sex and sibling identification. This information is collected by the schools in conjunction with parental education and is available from school records.

4. Analytical strategy

4.1. Pre-analysis plan. We pre-registered our hypotheses and study design at the Open Science Framework (35). In our pre-analysis plan we described the sample inclusion criteria, key variables, and hypotheses to be tested. In particular, the pre-analysis plan outlines the student monitoring system, the difference-in-differences setup, the school grades that we choose to include, the operationalization of parental education and past performance, and possible strategies to deal with attrition. Moreover, we proposed 5 hypotheses based on a reading of previous literature on learning loss due to temporary school closures or during summer recess:

H1: Students are learning less during lockdown than in a typical year.

H2: Learning loss is greater among students from less-educated homes.

H3: Learning loss is greater among low-performing students.

H4: Learning loss is greater among boys than girls.

H5: Learning loss is greater in Maths than in Reading or Spelling.

Among the pre-registered hypotheses, H1 and H2 receive support. Contrary to H3 and H4, we found no marked variation by prior performance or gender. H5 was not borne out either, but in supplementary analyses in Section 7.13 it emerges that results for Maths do appear to be somewhat more robust to specification than other subjects.

In our pre-analysis plan we also committed to making any deviations from protocol explicit in our final publication, and we do so here. Deviations are mainly due to one of two reasons: either the data contained information that made it possible to expand our analysis (e.g., with school and family fixed effects), or we added components to our model to ensure that identifying assumptions were satisifed (e.g., terms for year trend and date tested). In addition to the steps outlined in our pre-analysis plan, we extended our study design as follows:

- We had originally proposed to analyze test scores in the three subjects Maths, Spelling, and Reading. As results across these subjects were similar, we decided to summarize them using a composite score in our main analysis (Section 3.1), which was not part of the pre-analysis plan. We still display additional results for the separate subjects as originally intended.

- We had originally envisioned analyzing test scores on an absolute scale such that learning progress and not just changes in relative rank could be quantified. After consultation with the test producer (CITO), it became apparent that raw scores on the various tests were not possible to compare on the same scale. We therefore created the rank transformed variables that form our outcome (Section 3.1).
- To test the validity of our identification strategy, we ran placebo tests where we estimated our treatment effect across all years prior to 2020 (Section 7.2), as well as robustness analyses omitting single counterfactual years (Section 7.3). It then became apparent that we needed to adjust for the secular time trend and the number of days between tests to satisfy the parallel trends assumption underlying our difference-in-differences analysis.
- To adjust for attrition in the treatment year, we adopted a larger number of strategies than we had originally envisioned in our pre-registration. In particular, in matched analyses we were able to include a larger number of covariates than originally described (Section 7.4). Moreover, school and family fixed effects (Section 7.6–7.7) were not part of our original pre-analysis plan but emerged as the possibility became apparent after having accessed the data, as did the idea of analyzing schools with near-complete retention (Section 7.5).
- The "3-minute tests" of learning readiness were not part of our pre-analysis plan (Section 3.1 and 7.8). We were aware of these tests at the time of pre-registration but excluded them from our plan precisely because learning loss would be less likely to manifest here. In early presentations of our work, however, the question of "day of exam" effects came up and it occurred to us that these tests could be used to analyze this issue.

4.2. Identification strategy. Estimating the effect of school closures on student achievement raises several challenges. A naive approach would be to compare average national test scores following school closures to average national test scores in a previous year. However, this ignores the considerable fluctuation in performance that can occur due to changes in student composition or other factors from one year to the next. It is therefore vital that achievement measures are collected both before schools closed and after they reopened, so that progress in this period can be compared to progress during the same period in previous years. Still, if not all students return to school following reopenings, differences in the composition of test takers from the mid-year to the end-of-year test may bias estimates. In our analysis, we only include students who take both the mid-year test, before schools closed, and the end-of-year test, after schools reopened. This is, in effect, a differences-in-differences design (36):

$$\Delta y_i = \alpha + \delta T_i + \epsilon_{ij}, \quad [1]$$

where $\Delta y_i = y_i^{end} - y_i^{mid}$ is an individual student's relative movement in the achievement ranking from the mid-year to the end-of-year test, T_i is an indicator for the treatment year 2020, and ϵ_{ij} is an i.i.d. error term clustered at the school level. The coefficient δ thus captures overall learning loss due to the pandemic.

This specification deals with the fact that the composition of test takers may differ between the mid-year and end-of-year test by ensuring that only students present at both occasions contribute to the estimation. However, it does not deal with factors other than the pandemic that may influence achievement growth from one year to the next, neither with the fact that the composition of test-takers in the treatment year may differ from that in comparison years. To adjust for global factors differing between years, we therefore include a further vector of control variables $\mathbf{Z}_i$:

$$\Delta y_i = \alpha + \mathbf{Z}_i'\gamma + \delta T_i + \epsilon_{ij}. \quad [2]$$

In our baseline specification, $\mathbf{Z}_i$ includes a linear trend for the year of testing and a variable capturing the number of days between the two tests. Both these factors are important. Prior to the pandemic, the rate of progress between the two tests increased incrementally (Fig. S3) and it is arguable that the same trend would have continued unabated in absence of the pandemic. Moreover, end-of-year tests occurred on average later in the year during 2020 compared to previous years. Since it is well documented that test scores tend to improve with instruction time (37, 38), a credible counterfactual would have to take this difference in timing into account. In supplementary analyses, we show how the results differ if we do not account for time trends or testing date.

To deal with differences in the composition of students between treatment and comparison years, we pursue several strategies. The first is simply to include a set of student characteristics $\mathbf{X}_i$. We first use this setup including one variable at a time to assess heterogeneity in the treatment effect, interacting each student characteristic X_i with the treatment indicator T_i:

$$\Delta y_i = \alpha + \mathbf{Z}_i'\gamma + \beta X_i + \delta_0 T_i + \delta_1 T_i X_i + \epsilon_{ij}, \quad [3]$$

where X_i is one of: parental education, student sex, or prior performance. We also estimate separate models for each school grade. In the next step, we add all student covariates jointly as control variables:

$$\Delta y_i = \alpha + \mathbf{Z}_i'\gamma + \mathbf{X}_i'\beta + \delta T_i + \epsilon_{ij}, \quad [4]$$

where $\mathbf{X}_i$ is a vector containing parental education, student sex, and prior performance. Our dataset includes not only student covariates but also school characteristics, and potential interactions between variables. A flexible way to adjust for high-dimensional variation is through weighting schemes that ensure that characteristics are balanced between comparison and treatment group. Rosenbaum and Rubin (39) show how a large set of potential confounders can be reduced to a single propensity score, capturing the conditional probability of treatment. This approach proceeds in two steps: first by estimating the probability of treatment conditional on all observed covariates, and second by estimating the main outcome equation while balancing on the propensity score. The propensity score $\hat{p}(\mathbf{X}_i)$ is estimated from a logistic regression of the treatment indicator on the set of covariates. It is possible to incorporate it in several ways but we use it to construct a set of regression weights (40):

$$\mathbf{E}[\Delta y(\widehat{0) \mid T} = 1] = \frac{\sum_{\{i|T=0\}} \Delta y_i v_i}{\sum_{\{i|T=0\}} v_i}, \tag{5}$$

where the weight v_i of each observation is related to the propensity score through the equation $v_i = \frac{\hat{p}(\mathbf{X}_i)}{1-\hat{p}(\mathbf{X}_i)}$. While this approach ensures that covariates are balanced asymptotically, this may not always hold in practice. An alternative approach is therefore proposed by Hainmueller (41), that uses maximum entropy weights to balance comparison and treatment groups directly on the observed covariates. Building on the above example, this approach estimates the counterfactual in absence of treatment as follows:

$$\mathbf{E}[\Delta y(\widehat{0) \mid T} = 1] = \frac{\sum_{\{i|T=0\}} \Delta y_i w_i}{\sum_{\{i|T=0\}} w_i}, \tag{6}$$

where w_i is a weight chosen to minimize the entropy distance metric, $\log(w_i/q_i)$:

$$\min_{w_i} H(w) = \sum_{\{i|T=0\}} w_i \log(w_i/q_i), \tag{7}$$

with $q_i = 1/n_0$ denoting a base weight, and implemented subject to a set of R balancing constraints:

$$\sum_{\{i|T=0\}} w_i c_{ri}(\mathbf{X}_i) = m_r, \quad r \in 1, \ldots, R. \tag{8}$$

A further set of normalizing constraints ensure that weights w_i are non-negative for all i where $T = 0$, and that they sum to unity: $\sum_{\{i|T=0\}} w_i = 1$. In both the propensity score and maximum entropy balanced analyses, we adjust for a large set of covariates including interaction terms between all individual variables as well as school disadvantage, ethnic composition, and school denomination (Section 7.4). Across these models, we adjust for compositional differences using balancing weights while including the vector $\mathbf{Z}_i$ for testing year and date as standard regression controls.

All these three approaches—regression adjustment, propensity score weighting, and maximum entropy balancing—represent different ways of achieving balance on observed covariates but are vulnerable to unobserved sources of heterogeneity. In additional analyses, we make use of the fact that students are nested within schools and families to estimate fixed-effects designs (42). This allows us to adjust for any time-invariant confounding at the school or family level, whether due to observed or unobserved sources of heterogeneity. The fixed-effects design can be written:

$$\Delta y_i = \sum_{j=1}^{J} \alpha_j J_{ij} + \mathbf{Z}_i'\gamma + \mathbf{X}_i'\beta + \delta T_i + \epsilon_{ij}, \tag{9}$$

where $J_{ij} = \mathbf{1}_{J_i=j}$ is a binary indicator equal to one if unit i belongs to cluster j, and zero otherwise. We implement two versions of this model: one where the J_{ij} group identifiers are school level indicators grouping all students within the same school, and one where they are family indicators taking the same value for siblings. Finally, we estimate a version of the school-level model where we allow the estimated learning loss to vary by school, by introducing a school-specific intercept α_j and treatment coefficient δ_j (43):

$$\Delta y_i = \alpha_j + \mathbf{Z}_i'\gamma + \mathbf{X}_i'\beta + \delta_j T_i + \epsilon_{ij}, \tag{10}$$

$$\alpha_j \sim N(\mu_0, \sigma_0^2), \quad \delta_j \sim N(\mu_1, \sigma_1^2) \tag{11}$$

This model is useful because it lets us explore how schools differ in the impact suffered from the pandemic, and how this depends on school-level characteristics such as school disadvantage and ethnic composition (Section 7.9). We estimate all models in the R statistical computing environment using packages listed in Section 4.4 below.

4.3. Effect size conversions.

4.3.1. Percentiles and standardized effects. Our effect sizes are expressed on the scale of percentiles. In educational research it is common to use standard-deviation based metrics such as Cohen's d (44):

$$d = \frac{\bar{x}_1 - \bar{x}_2}{\sigma_p}, \quad [12]$$

where $\bar{x}_1 - \bar{x}_2$ is the difference in means between treatment and comparison groups and σ_p is the pooled standard deviation. To convert between treatment effects on the percentile scale and standardized effects, we rely on the lesser known U_3 metric also proposed by Cohen (45), which describes the overlap between two distributions. Specifically, U_3 is defined as the proportion of the comparison group exceeded by the upper half of cases in the treatment group. Conversion between U_3 and d can be done with the following equation:

$$d = \Phi^{-1}(U_3), \quad [13]$$

where Φ^{-1} is the inverse cumulative standard normal distribution. While this conversion applies to the normal case, U_3 is defined such that it is invariant to any rank-preserving transformation. Hence, we can apply the same conversion to our percentile scores under the assumption that they came from an underlying normal distribution.

For two identical distributions with no difference in means, the upper half of cases in the treatment group will exceed exactly half of the cases in the comparison group. In this case, $U_3 = 0.50$ and $d = \Phi^{-1}(0.50) = 0$. A difference of -3 percentiles in the treatment group vs the comparison group implies that $U_3 = 0.50 - 0.03 = 0.47$. Hence, the standardized effect size equivalent becomes $d = \Phi^{-1}(0.47) = -0.075$. More generally, with "small" or "medium" effect sizes in the range $d \in [-0.5, 0.5]$, Cohen's U_3 implies a conversion factor of 0.025 standard deviations per percentile.

4.3.2. Benchmarks for annual progress. Another way to quantify treatment effects is as a proportion of gains made in a normal year. In doing so, it is common to rely on age-specific benchmarks derived from standardized tests. For example, the World Bank's simulations of COVID-19 induced learning loss assume a progress rate of 0.40 *SD* per year based on students in the Programme for International Student Assessment (46). In the US, Bloom, Hill, and coauthors (47) report annual gains for our age range of 8–11 years based on nationally normed tests in Maths and Reading. Their reported annual gains range from 0.60 *SD* at age 8 to 0.32 *SD* at age 11 (average 0.42 *SD*) in Reading and from 0.89 *SD* at age 8 to 0.41 *SD* at age 11 (average 0.60 *SD*) in Maths.

To derive appropriate benchmarks for our population, we estimate weekly test score gains in our data using tests taken in the comparison years 2017–2019. Specifically, we estimate the following regression equation:

$$\Delta y_i = \sum_{k=1}^{K} \alpha_k K_i + \lambda W_i + \epsilon_{ij}, \quad [14]$$

where Δy_i is the difference score defined earlier, K_i is a set of indicators for the three years 2017, 2018, 2019, and W_i is a term capturing the number of weeks (i.e., $\frac{days}{7}$) elapsed between the mid-year and end-of-year test. In Table S1 we provide estimates of the parameter λ, capturing weekly learning progress in percentile scores, for the whole sample and for different subgroups. In contrast to US studies, progress in our data does not appear to be very dependent on age but is mostly in the range of 0.3–0.4 percentiles per week. Gains in Reading are smaller, likely due to the non-mandatory character of these tests (Section 3.1 and 5.2). Anecdotally, when testing is not mandatory, schools may choose to give preference to weaker students in administering tests which could bias the estimates. We therefore focus on progress in Maths and Spelling which is more representative of the population.

A progress of 0.3–0.4 percentiles per week implies an annual gain of 12–16 percentiles when multiplied by the 40 weeks of a school year. Alternatively, assuming (generously) that children learn at the same rate throughout the 52 weeks in the year, the rate becomes 16–21 percentiles per year. Applying the inverse cumulative standard normal transformation described in Eq. [13] gives us the following benchmarks for annual gains of 12, 16, and 21 percentiles: 0.31 *SD*, 0.41 *SD*, 0.55 *SD*. For our preferred treatment effect estimate of 0.075 *SD* and the middle estimate of 0.41 *SD* annual progress, this translates into 0.075/0.41 = 18.29% of a school year. Alternatively, we may take the estimated treatment effect on the original percentile scale (3 percentiles) and divide it by an annual estimated gain of 16 percentiles to get 3/16=18.75% of a school year. A different way to arrive at the same conclusion is to take the estimated treatment effect of 3 percentiles and divide it directly by the weekly estimated gain of 0.4 percentiles which gives us an effect equivalent to 3/0.4=7.5 weeks, or 18.75% of a 40-week school year.

4.4. Statistical software. Analyses were done in `R` (version 4.0.3). Code underlying all our analyses can be found on https://github.com/MarkDVerhagen/Learning_Loss_COVID-19. We used the `estimatr` package to cluster standard errors at the school level, as well as to include school and family fixed effects. We used the `lme4` package for the multilevel analyses. To adjust our sample using matching and weighting techniques, we relied on the `WeightIt` and `cobalt` packages. We are thankful to the `R` community, in particular to the `tidyverse`, `broom`, and `lubridate` data wrangling libraries, and the `data.table` library that helped us greatly speed up data processing. All computations were done on a machine running Mac OSX 10.17.7. A full list of libraries used can be found on the aforementioned GitHub page.

- `Estimatr`: https://cran.r-project.org/web/packages/estimatr/estimatr.pdf
- `lme4`: https://cran.r-project.org/web/packages/lme4/lme4.pdf
- `WeightIt`: https://cran.r-project.org/web/packages/WeightIt/WeightIt.pdf
- `Cobalt`: https://cran.r-project.org/web/packages/cobalt/cobalt.pdf
- `Tidyverse`: https://cran.r-project.org/web/packages/tidyverse/tidyverse.pdf
- `broom`: https://cran.r-project.org/web/packages/broom/broom.pdf
- `lubridate`: https://cran.r-project.org/web/packages/lubridate/lubridate.pdf
- `data.table`: https://cran.r-project.org/web/packages/data.table/data.table.pdf

5. Quality control

5.1. Representativeness. We obtained access to the data through the Mirror Foundation, and an educational analytics provider with which the foundation collaborates (Section 2.3). Selection into the sample is thus mediated through a school's use of the educational analytics service. There might be concerns whether this selection is non-random, and hence how well our sample represents the universe of schools in the Netherlands. In Fig. S2 we evaluate this question by inspecting the distribution of observable characteristics in our sample and the population. The sample distribution mirrors the population on most observables: school size, denomination, urbanity, parental education, and school disadvantage, with a few exceptions. There is some over-representation of mid-sized schools (101–200 students) and sampled schools are located in slightly more ethnically diverse neighborhoods on average. Importantly, the relative representation of school type (e.g. public or Christian) is near identical to that in the population, as is the distribution of parental education within schools. Schools in our sample are also close to the population distribution on the newer composite indicator of school disadvantage (Section 2.2 and 3.2).

This leaves the possibility that our sample is selected on unobserved characteristics. In particular, it is possible that adopters of the analytics service are especially invested in digital infrastructure and data-based accountability. Although all schools are mandated to use a digital interface for student monitoring, many existing solutions are simple data management tools with limited analytical functionality. To the extent that the schools in our sample are more invested in digital infrastructure, they may have been better equipped to cope with online learning which could lead us to underestimate the impact of pandemic school closures. Another consideration is ability to pay, given that the platform service is offered on a paid subscription basis. However, the cost of the service is minor relative to a school budget: 1,500 euro annually, which corresponds to 3% of a single teacher's salary (50,000 euro) or less than 0.1% of a typical school budget (2 million euro). As the main determinant of school funding until 2019 was the parental education of the student body, the fact that this does not differ markedly between the sample and the population (Fig. S2) corroborates that economic considerations are not a major determinant of service uptake.

5.2. Missing data. We define our analytical sample as all observations in the relevant grades in our database for which there is valid information on sex. Table S2, top panel, shows missing data on covariates by year and grade: sex, parental education, prior performance, school ID, school denomination, school disadvantage, neighborhood ethnic composition, and family ID. There is no missing data on parental education, which is information that schools have to collect by law. About 6% lack performance data from the previous year. This proportion is similar across all grades and years, albeit slightly large in earlier grades. All students are nested within a school, and 96% could be linked to contextual school variables (all but 48 schools). There is a high proportion of missing data on the family identifier that we use for fixed-effects models (42%). This reflects the fact that data are missing for all students who do not have a sibling in the same school. Our sample in family fixed-effects models becomes even smaller, as it is not enough that a student has a sibling in the school but there also needs to be within-family variation in treatment status.

Table S2, bottom panel, shows missing data on test scores by grade and year. Test score data are missing for a significant share of the sample, with missing values being higher in Reading than in either Maths or Spelling. This is due to the regulation of the student monitoring system, which only requires schools to report student achievement in Maths and Spelling (Section 3.1). Consequently, some schools are choosing to test students in Reading only at the mid-year test (most common in the youngest grade) or only at the end-of-year test (more common in the later grades). In the treatment year 2020, there is also a significant amount of missing data on the end-of-year test following students' return from school closures. This missingness will bias our estimates if it is selected on the outcome variable, that is, if only those students who tend to over- or underperform relative to the middle-of-year test are returning to school. Reassuringly, Table S3 shows that the missing data is almost perfectly balanced by prior performance: the composition of top, middle, and bottom performers is similar in comparison and treatment years.

5.3. Measurement error.

5.3.1. Parental education. Most of the right-hand side variables in our analysis—year, grade, testing date, sex—are likely to be measured with little error, but parental education is potentially of concern. (We discuss prior performance together with outcome variables in the next section.) Discussions of measurement error typically start from the "classical" assumptions that error is simply a white-noise term uncorrelated with the true values of the variable of interest and with the regression residual (48, 49). Such error leads to bias toward the null when it occurs in a predictor variable. For categorical variables, error is necessarily *mean-reverting*, that is, negatively correlated with true values. In this case, the classical assumptions cannot hold and are usually replaced by the assumption that any error is *non-differential*, meaning that misclassification does not depend on the regression outcome. The consequences here are similar, in that any misclassification will bias regression estimates toward the null (50, 51).

Parental education is likely to have relatively high reliability in our data considering that it is reported by parents themselves, rather than by students which is known to cause problems (49, 52, 53). Data collected by questionnaire is also superior to administrative register data in some respects, in particular, in that incorrectly matched records and underreporting of foreign credentials can be avoided (54–56). However, the definition of the categories means that we are only able to separate out the very bottom of the educational distribution, and we are thereby likely to miss important sources of variation in parental education in Dutch society. Here we are constrained by the official definition of school funding weights (Section 2.2 and 3.2), which unfortunately prevents us from coding our data differently. Nevertheless, the uneven size of the categories should be mentioned as a limitation.

5.3.2. Test scores. The tests we use have been developed by the Dutch National Institute for Educational Measurement, CITO (Section 2 and 3.1), and have gone through extensive psychometric validation. The reliability of these tests is in most cases around 0.90 (24–26). Indeed, in Table S4, we use our data to show that the correlation of the composite achievement score with tests taken over the previous year exceed 0.80 in both treatment and comparison years. The scope for measurement error in test scores to influence our analysis is therefore limited. Nevertheless, it merits discussion what influence any remaining error might have on our estimates. As test scores enter both into our outcome measures and in analyzing potential heterogeneity by prior performance, we discuss each of these in turn.

In contrast to predictor variables, classical measurement error is of limited consequence when it occurs in outcome variables—except that it increases sampling variance. Measurement error in an outcome might still bias estimates if it is mean-reverting, that is, the error term is negatively correlated with true values (48). In this case, the consequences are similar to classical measurement error in a predictor: estimates are biased toward the null. As with categorical variables, mean-reverting error often occurs in bounded variables due to floor and ceiling effects. A percentile score is bounded, so in principle, any error here will be mean-reverting. The outcome of our analysis, however, is the difference between two percentiles. Unlike the underlying scores, this variable is approximately normally distributed and well within the range of theoretical bounds $[-99, 99]$ (main manuscript, Fig. 2). We therefore believe that the classical assumptions are a valid approximation, and error in our outcomes is unlikely to substantially bias estimates.

The use of test scores on the right-hand side of a regression raises particular issues due to the correlation of test scores over time. In particular, regression to the mean entails that students in the bottom will always tend to improve while those in the top will deteriorate, on average. If achievement is measured with error, it is hard to distinguish actual improvement or deterioration from such artifacts (30). To reduce the risk of errors, we use all of a student's test scores in the last year when measuring prior performance (Section 3.2). In Table S4 we show that prior performance is a reliable predictor of current performance: correlations with subsequent performance on the mid-year and end-of-year test exceed 0.80 in both comparison and treatment years. At the same time, by using data from the prior year we avoid a mechanical correlation with subsequent change in performance: the correlation between prior performance and the change score that is our outcome is less than 0.05 in absolute size in both comparison and treatment years. We therefore believe there is limited risk that regression to the mean might bias our results.

5.4. Confounding. A main concern in our data is that we are not able to observe immigrant background of individual students. This raises the question of to what extent ethnic minority status is proxied for by our measure of parental education (Section 2.2 and 3.2). Given that the categorization distinguishes only among the very lowest levels of education, an important question is whether a large proportion of those classified as less educated in our sample are non-Western immigrants. One potential reason for such overrepresentation is if foreign credentials are not observed. This is a common problem in administrative register data, but is less likely in our data which was collected by parental questionnaires. However, non-Western immigrants are still likely to be overrepresented in the bottom groups if only because most non-Western countries have lower average levels of schooling than the Netherlands.

To assess the overlap, in Table S5 we present aggregate population data on the joint distribution of parental education and immigrant background among school children in 2018–2019 from Statistics Netherlands. These data confirm that immigrant parents are overrepresented in the "lowest educated" category (88% vs 27% in the total population) but much less so in the "low educated" category (35% vs 27% in the total population). Thus, an absence of large differences in treatment effect between the "low" and "lowest" educated groups indicates that the greater vulnerability of children from less-educated homes is not primarily driven by immigrant background. Note that these figures are based on population registers, which may exaggerate the overlap between low education and immigrant origin relative to survey data for the reasons discussed above.

6. Descriptive statistics

Table S3 shows summary statistics for the full sample broken up by comparison and treatment groups. Because of the large sample size, most differences are statistically significant even when they are quantitatively unimportant. For example, the proportion non-Western immigrants in the school's neighborhood is 0.17 in both comparison and treatment groups but nevertheless different (at the third decimal point) at a level that passes the 0.1% significance threshold. Substantive differences between comparison and treatment groups are thus minor, with two exceptions: students in the highest grade (age 11) are underrepresented in the comparison group but not in the treatment group. Table S3 shows that students in this grade are more likely to skip the end-of-year test in a normal year, with data missing nearly completely in the first year 2017. Moreover, the duration between the mid-year and end-of-year test is on average 9 days longer in the treatment year 2020, as is also visible in Fig. 1 of the main manuscript. In our main analysis we include days between tests as a control variable (Section 4.2), but we show results excluding this covariate in Section 7.12.

Table S6 shows the average performance by test, year, and student characteristics. Boys perform better in Maths, but worse in Reading and Spelling, while both genders perform similarly on tests of Learning readiness. Along parental education, there are differences up to 20 percentiles between the high and lowest educated groups. Unsurprisingly, the most important predictor of performance is prior performance with the top, middle, and bottom performers separated by on average about 20–25 percentiles each. There are no marked differences in the rate of progress across subgroups, that is, students belonging to various groups tend to retain their relative placement in the achievement distribution between the mid-year and end-of-year tests. There is a consistent trend in years prior to treatment, where relative progress between mid-year and end-of-year tests increased by on average about a half percentile point per year. This is in turn driven by a decline apparent in both tests, but more so in the mid-year than in the end-of-year test. Fig. S3 presents the trend for the difference between the two tests, showing a clear break in the treatment year 2020. In our main analysis, we assume that progress would have continued at the same pace in absence of the pandemic. In additional analyses in Section 7.11, we relax this assumption and present results comparing 2020 only to the previous year 2019, assuming a flat trend.

7. Additional results

7.1. Regression tables. In Table S7–S11, we display regression results underlying Fig. 3 in the main manuscript, as well as additional analyses by subject and subgroup. Table S7 displays the main effect reported in main manuscript Fig. 3, and separate results by subject domain. Table S8 shows results by parental education for the composite score as reported in main text Fig. 3, and for separate subjects. Table S9 does the same for student sex and Table S10 does so for prior performance. Table S11 displays separate analyses by grade. The results are similar across all grades, but slightly weaker for the highest grade (age 11). This is likely related to the large amount of missing data in this grade (Section 5.2 and Table S2). In Table S12, we report additional regression results simultaneously controlling for all individual-level covariates: sex, parental education, prior performance. This does little to change the treatment effects, which is unsurprising given that treatment status is largely unrelated to student observables (Section 6 and Table S3). In Table S13 we restrict the sample to only those students with a valid score in all three subjects, again with similar results. This last set of results is presented visually in Fig. S4.

7.2. Placebo analysis. In Fig. S5 we perform a placebo analysis on non-treated years. We do so by keeping the specification identical to our main analysis but excluding the actual treatment year and, in turn, assigning treatment status to each of the three comparison years. Doing so reveals few significant effects, and those that are so by chance are mostly in the opposite direction of the results reported in the main manuscript. Our identification strategy thus appears robust to false positives, and if anything, is likely to underestimate the treatment effect somewhat given the small bias towards a positive treatment effect in two of three comparison years. Crucially, however, the pooled effect is not significantly different from zero in any year. In Section 7.13 and Fig. S21, we further show (using 2019 as the placebo year) that the placebo effect remains non-significant throughout specifications, except for analyses that discard with our control for a linear trend in year (cf. Fig. S3). This is a leading reason why we choose to include a control for year in analyses presented throughout the main manuscript (Section 4.2).

7.3. Year exclusions. To confirm that our results are not driven by any one comparison year, we re-estimate our main specification dropping comparison years one at a time. Results from these analyses are reported in Fig. S6. Estimates are less precisely estimated but of a similar magnitude and significantly different from zero throughout. Dropping the year immediately preceding treatment, 2019, reduces both the precision and the magnitude of the estimated treatment effects somewhat. This is driven by the absence of a clear trend in Maths achievement between 2017 and 2018, as shown in Fig. S3. The size of the estimated learning loss in Reading and Spelling remain undiminished. Moreover, the qualitative results remain unchanged across all these year exclusions. Specifically, the difference in effect size between students from high- and low-educated homes remains of a similar magnitude and is significant at the 0.1% level throughout these analyses.

7.4. Covariate balancing. In Table S12, we report regression results including individual-level control variables. To adjust for a larger set of observables, including school characteristics and potential higher-order interactions, in Fig S7–S8 we further implement propensity score weighting and balancing using maximum entropy weights (Section 4.2). In these analyses we include the same individual-level covariates as earlier—sex, parental education, prior performance—but also two- and three-way interactions between them, a student's school grade, and school-level covariates: school denomination, school disadvantage, and neighborhood ethnic composition. Fig. S7 shows that both the entropy balancing and the propensity score weighting method achieve a sample that is balanced on the relevant characteristics. Fig. S8 displays our main results using each weighting method. Regardless of weighting schemes, both estimates of learning loss are highly similar and correspond closely to our main specification as reported in Fig. 3 of the main manuscript.

7.5. Near-complete schools. Our re-weighted analyses in Section 7.4 adjust extensively for observed covariates. Still, given the high loss to follow-up apparent in Table S2, selection on unobservables may nevertheless remain. As one way to address this issue, we repeat our baseline analysis while restricting the sample to only those schools where at least 75% of students were tested across both testing occasions in the treatment year (mid-year vs end-of-year). Table S14 reports the pooled treatment effect using this restriction, while Table S15 displays differential impacts by parental education. Fig. S9 presents the results for these and other subgroups visually. All results remains significant at the 0.1% level and close in magnitude to our original analysis. The overall treatment effect and disparities by parental education are, if anything, slightly larger than in our baseline analysis. This difference in effect size with respect to our baseline analysis is not statistically significant. Nevertheless, it bears noting that the direction of this difference is the opposite from what we would expect if results were driven by selective loss to follow-up.

7.6. School fixed effects. Another way to address selective loss to follow-up is by introducing school-level fixed effects (Section 4.2). This design discards all variation between schools which might have biased our results if, for example, schools that perform worse in previous years are over-represented in the treatment year. Table S16 shows results adding school fixed effects, while Table S17 does so for the interaction by parental education. Again, the estimated treatment effect is significant at the 0.1% level and remains similar in magnitude to our estimates reported in the main text. As in the analysis of near-complete schools in Section 7.5, both the overall treatment effect and disparities by parental education grow somewhat relative to the baseline set of results. Again, however, differences with respect to the baseline model are not statistically significant. In Fig. S10 we display the results for these and other subgroups visually. This plot confirms that our other results remain similar, and all qualitative conclusions remain unchanged.

7.7. Family fixed effects. Using a similar logic as in Section 7.6 above, we estimate fixed-effects models that discard all variation between families by introducing a separate intercept for each group of siblings identified in our data (Section 4.2). The results are displayed in Table S18–S19 and visually in Fig. S11. In many ways, the family fixed-effects design is our most powerful way to adjust for differential attrition between comparison and treatment years. It

removes any sources of heterogeneity that make siblings more similar than randomly chosen individuals, whether due to observed or unobserved factors. The tradeoff is that we have to restrict the sample to treated students where either they or a sibling is observed in a comparison year, which reduces our sample size by roughly 70%. This makes the estimates less stable, especially in the analyses by grade (Fig. S11). Nevertheless, the results remain qualitatively similar, and differences by parental education are larger in this specification than in any other. This could be due either to the removal of family-level heterogeneity or to the different sample used in this analysis. To address this question, in Table S20–S21 we re-estimate our baseline specification but using the same subsample as for the family fixed effects. Doing so reveals results of a similar magnitude, suggesting that differences with respect to our main set of results are primarily due to the different sample.

7.8. Learning readiness. As an alternative outcome, we assess students' performance on short 3-minute tests in oral reading fluency that are meant to test their learning readiness (Section 3.1). These tests are not designed to assess any part of the taught curriculum, but are taken in conjunction with the other tests and under similar circumstances. If our main estimates of learning loss reflect the cumulative impact of knowledge learned, we would expect these effects to be small or zero. In contrast, if our estimates of learning loss mainly reflect "day of exam" effects due to stress exposure, testing conditions, or lack of familiarity with the school setting, we would expect similarly large losses on both kinds of test. Fig. S12, top panel, reveals that the treatment effect on this outcome is on average 62% smaller than for our main outcome. Fig. S12, bottom panel, shows that this is not the case in a non-treatment year, where estimated null effects on the both tests are instead closely similar. We therefore conclude that "day of exam" effects are unlikely to be the main explanation for our results.

7.9. School variability. While school closures were deployed nationwide, the circumstances surrounding online learning were largely a matter for individual schools to handle. It is therefore likely that the response differed widely at the school level. Fig. S13–S14 report estimates from a mixed-effects model that lets the estimated learning loss differ between schools (Section 4.2). The results reveal considerable variation, with some schools seeing a learning slide of 10 percentiles or more, and others recording no losses or even small gains. In both cases, we plot the predicted school-level treatment effects against school-level socioeconomic disadvantage and the share of non-Western immigrants in the school neighborhood. The socioeconomic disadvantage scores are a composite that incorporates the education level, country of origin, duration of residence, and economic hardship of all parents in the school (Section 3.2). Losses are larger in schools with a high proportion of disadvantaged students and of immigrant background (Fig. S13), and this holds further when adjusting for individual-level covariates (Fig. S14).

7.10. Three-way interactions. In our main analysis, we find little evidence that the impact of the pandemic differs along any other dimension than parental education. Here we ask whether the greater vulnerability among students from less-educated homes is especially pronounced in some groups. For example, a commonly held hypothesis is that boys may display greater vulnerability to socioeconomic disadvantage (57–59). However, Table S22 reveals that the differential impact by parental education in our sample does not differ significantly by gender. Table S23 further breaks down the parental education differential in learning loss by prior performance. The absence of differential effects by prior performance is surprising in light of the fact that it correlates with parental education. Given this correlation, we would expect that a larger learning loss among children from less-educated homes translates into a corresponding difference by prior performance. Our analysis in Table S23 resolves this puzzle by showing that losses are especially concentrated among students from less-educated homes who performed well in the previous year.

7.11. Trend assumption. Fig. S3 shows that the rate of progress between the two tests increased incrementally in the years prior to the pandemic. By controlling for a linear trend in year (Section 4.2), our main analysis assumes a counterfactual where the trend apparent in pre-treatment years would have continued unabated in absence of the pandemic. In Table S24–S25 and Fig. S15 we replace this assumption by comparing the treatment year only to the previous year, 2019, and assuming a flat trend. We put less trust in this specification than that of our main analysis because, as Fig. S21 shows, it produces a spurious positive effect when applied to placebo years. This leads us to think that it will underestimate learning loss due to the pandemic. Indeed, Table S24 shows that the estimated treatment effect shrinks somewhat using this strategy, by about 21%. Nevertheless, the absolute differences in learning loss by parental education remain of a similar magnitude (Table S25). Fig. S15 also shows that learning losses in Maths appear undiminished, while the reduction is driven by Spelling and Reading. Other results remain qualitatively unchanged.

7.12. Days between tests. As shown in the main manuscript, Fig. 1, one salient difference between comparison and treatment years is that testing was delayed due to the pandemic school closures. Table S3 reveals that the end-of-year

tests occurred on average 9 days later relative to the mid-year test in the treatment year. In our main analysis we choose to adjust for this fact by including a linear control term for the number of days between tests (Section 4.2). Table S1 shows that instruction time in a normal year has a positive effect on students' test scores. It is therefore possible that part of our estimated learning loss is attributable to the choice of including this variable as a control term. We address this question in Table S26–S27 and Fig. S16, where we estimate models identical to our baseline specification but excluding the number of days between tests. Doing so, the estimated treatment effect shrinks by about 12% (Table S26). The absolute differences in learning loss by parental education remain undiminished (Table S27). Fig. S16 shows that our other results likewise remain qualitatively unchanged.

7.13. Specification curve. In the foregoing, we have tested various departures from our preferred specification by varying one model component at a time. A more exhaustive way to assess the robustness of results is to run all possible specifications that arise from combinations of analytical choices. Here we do so using a specification curve analysis (60). The underlying idea is to: 1) list the different analytical options that a researcher encounters in specifying the model, 2) define a model space based on all possible or reasonable combinations of these options, and 3) examine how the parameter of interest varies across the complete model space. We let the specification vary according to the following criteria:

- Individual controls: Whether to adjust for sex, parental education, prior performance, and school grade and different combinations thereof.
- Interactions: Whether the model includes interaction terms, including potential higher-order interactions, and different combinations thereof.
- Sample period: Whether to use all three comparison years (assuming a linear trend) or only compare the treatment year to the previous year (assuming the trend is flat).
- Fixed effects: Whether to include fixed effects at the school level, at the family level, or no fixed effects.
- Days between tests: Whether to adjust for the time elapsed between testing dates or not.

Fig. S17 displays more than 2,000 alternative treatment effect estimates for our main outcome, one for each model that arises from all combinations of the choices above. Estimated learning loss is broadly within the range from 2 to 3.5 percentiles. The most consequential choice turns out to be the inclusion of a linear trend in year. Excluding this term reduces the size of estimates as we have already showed in Section 7.11. Nevertheless, we prefer our main specification which includes this term, for reasons that appear in Fig. S21: applying the same set of specifications to a placebo year produces a spurious positive effect, which is not the case with our preferred specification (Section 7.2). The next most important pattern is that estimates grow in size when fixed effects at the school level, and (particularly) at the family level, are included. However, the larger effects in within-family analyses are in part attributable to the fact a smaller and selected portion of the sample is used (Section 7.7). Fig. S18–S20 repeats the specification curve exercise for each of the tree subjects: Maths, Spelling, and Reading. Results in Maths emerge as particularly robust to specification, while those in Spelling are the most sensitive. Conclusions about the model components which have most influence are broadly similar across these outcomes.

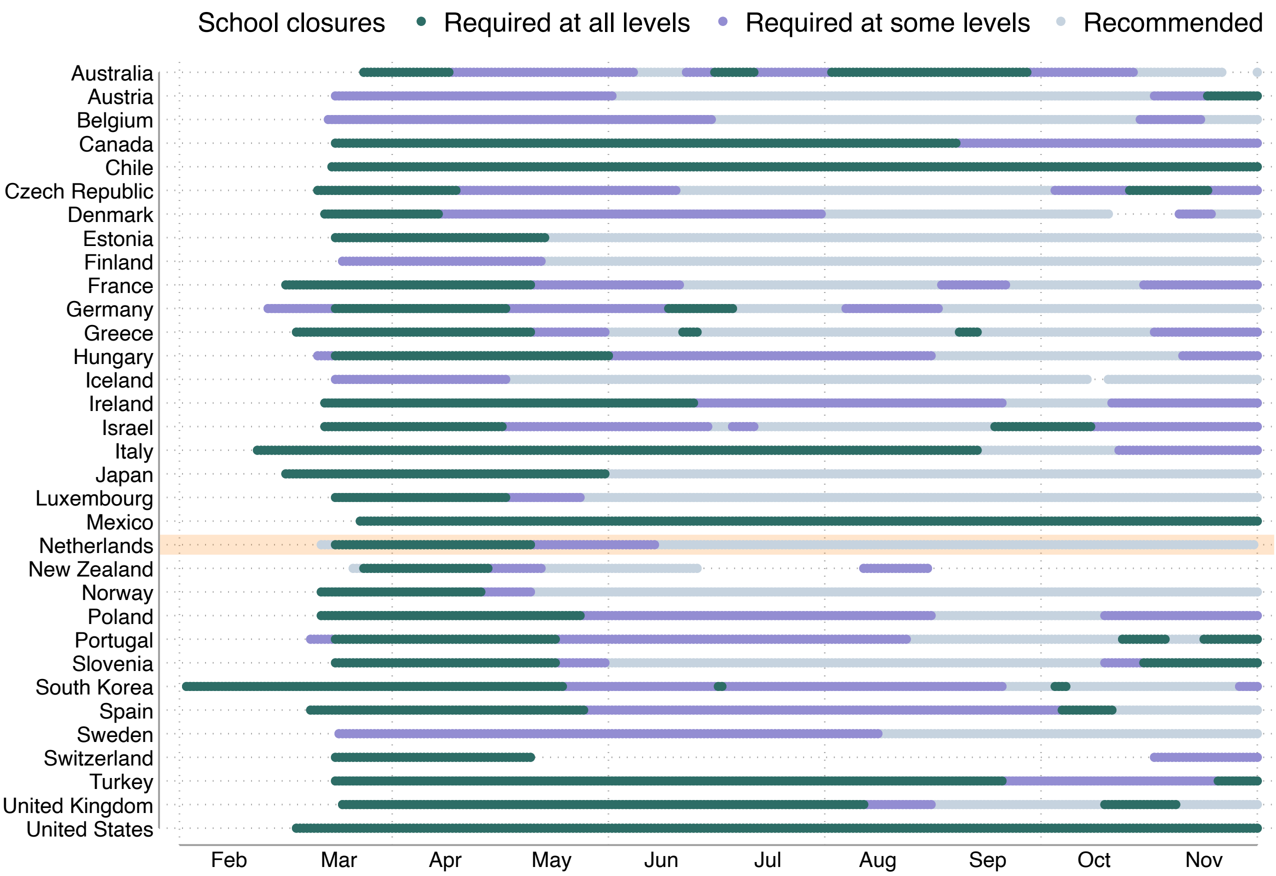

Fig. S1. School closures in the OECD. The graph shows the onset and duration of school closures in 33 OECD countries through November 2020, with the Netherlands marked in orange. Source: Oxford COVID-19 Government Response Tracker (https://covidtracker.bsg.ox.ac.uk/).

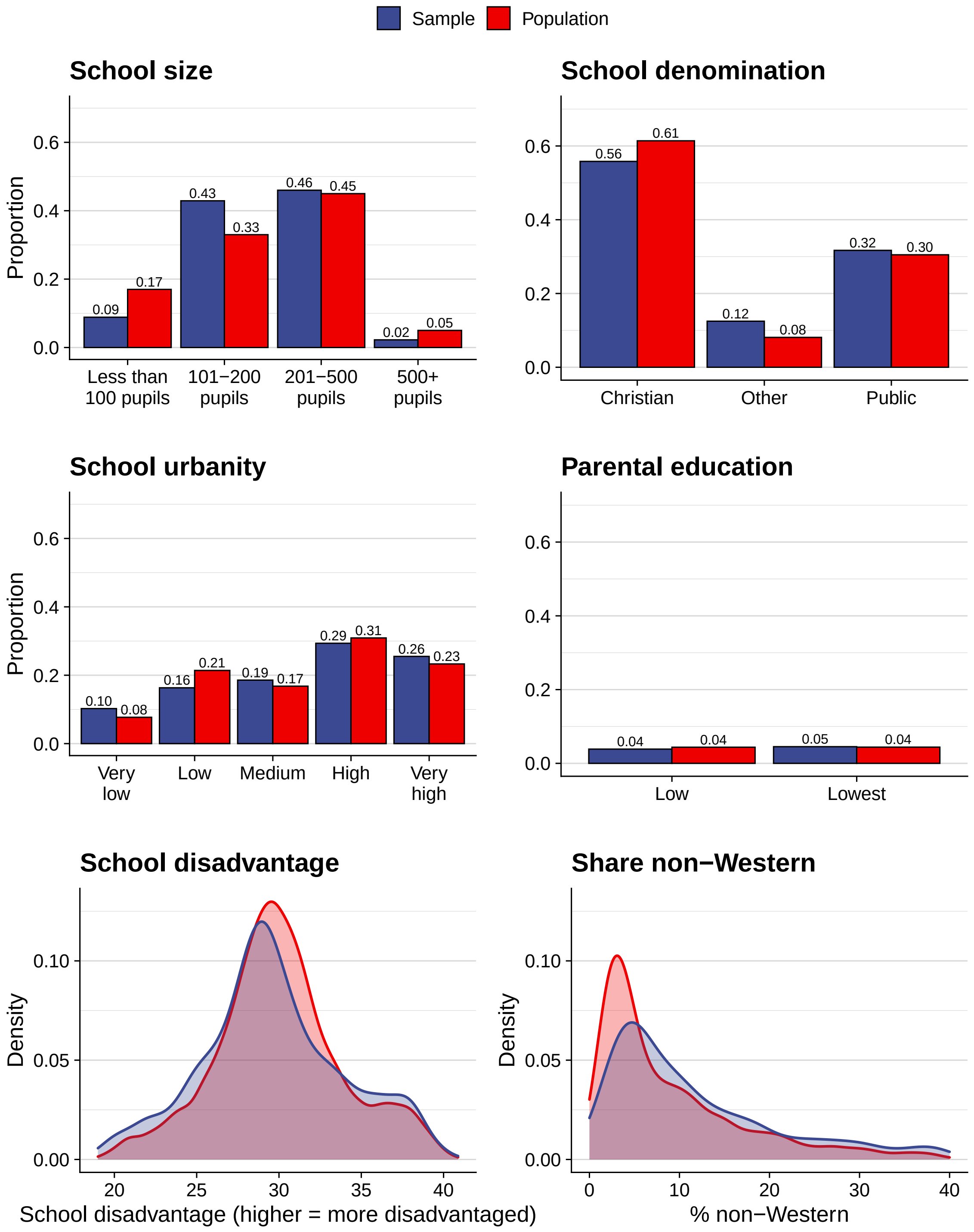

Fig. S2. Representativity of the sample. The graph compares the distribution of school characteristics in our sample, shown in blue, with that of the universe of primary schools in the Netherlands, shown in red. Source: Onderwijsinspectie (https://www.onderwijsinspectie.nl/trends-en-ontwikkelingen/onderwijsdata), CBS Statline (https://www.cbs.nl/nl-nl/dossier/nederland-regionaal/wijk-en-buurtstatistieken/kerncijfers-wijken-en-buurten-2004-2019).

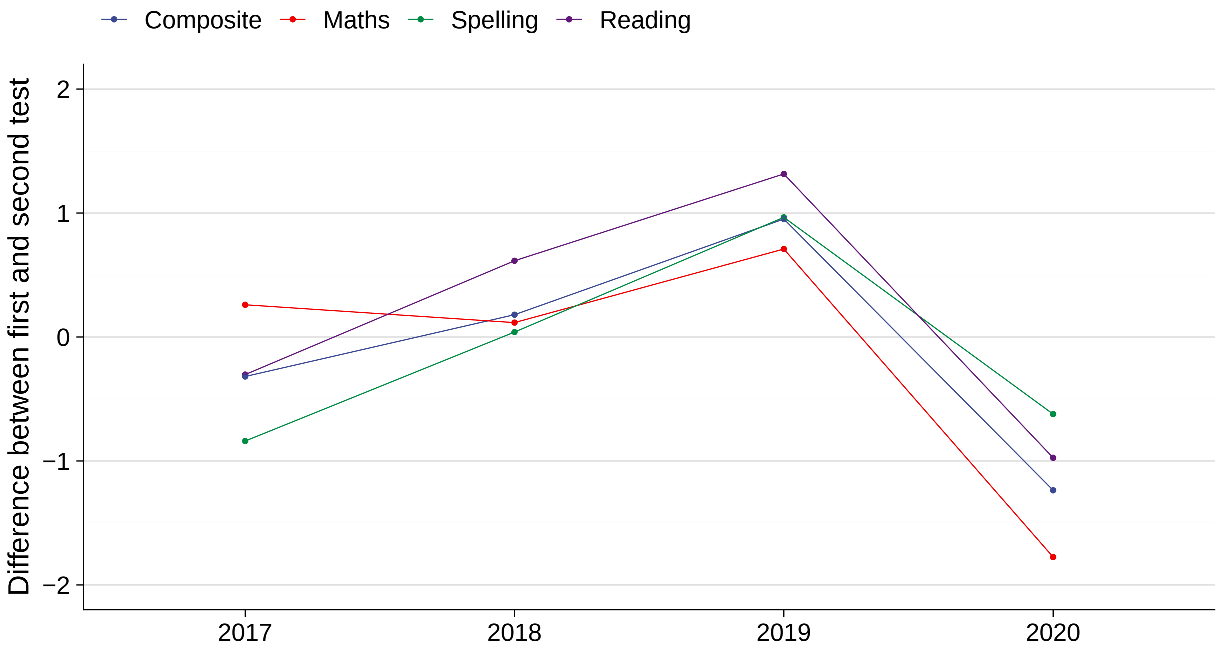

Fig. S3. Trends in student progress. The graph shows trends in the difference between mid-year and end-of-year test scores by subject and year.

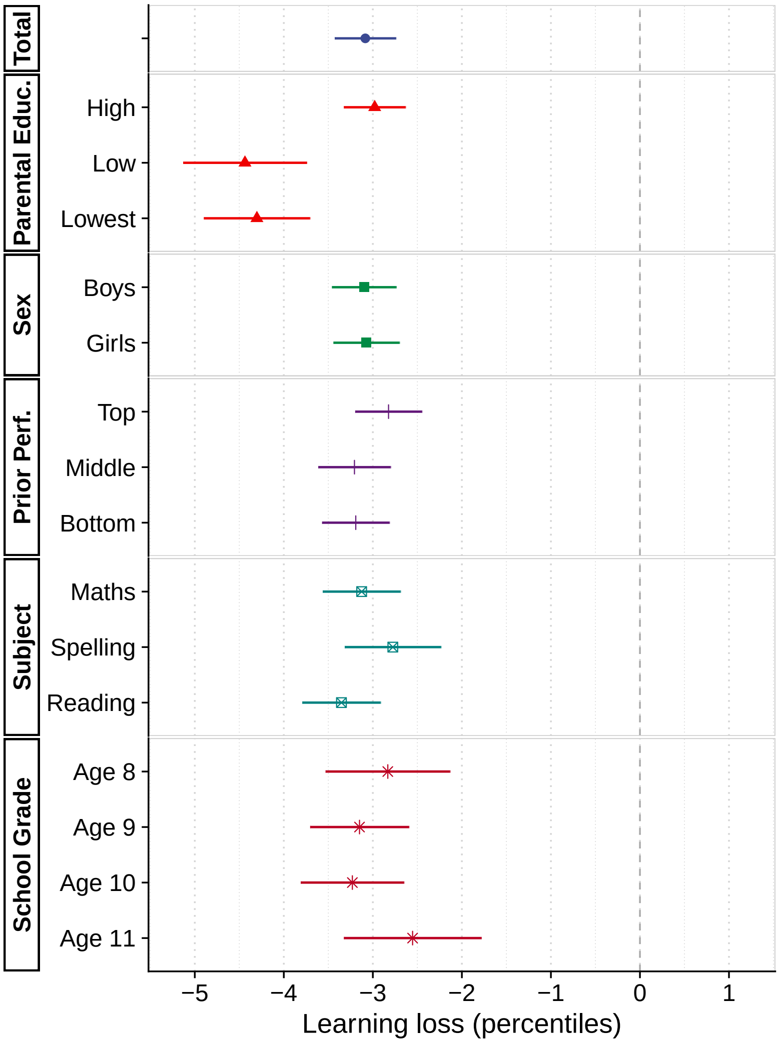

Fig. S4. Results with complete subject scores. The graph shows results from a specification identical to our main analysis except the sample is restricted to students with complete scores in all subjects.

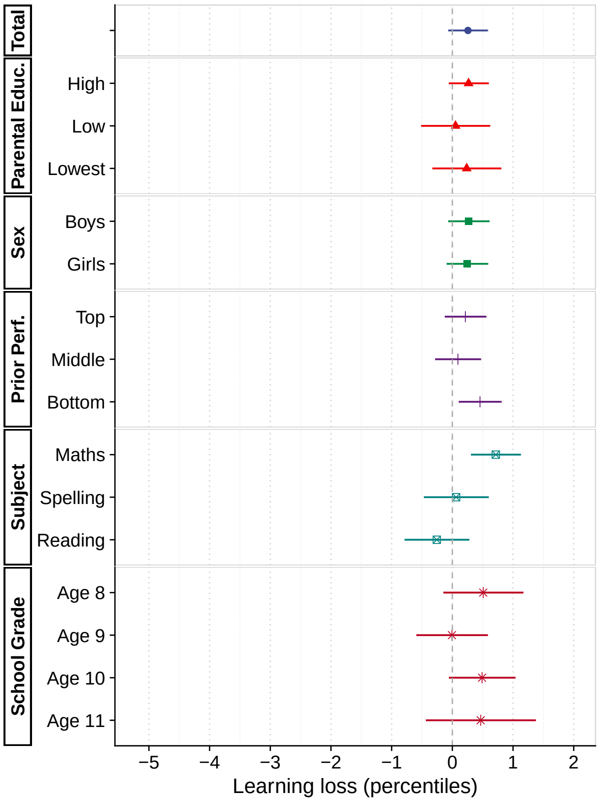

(a) 2017 as treated

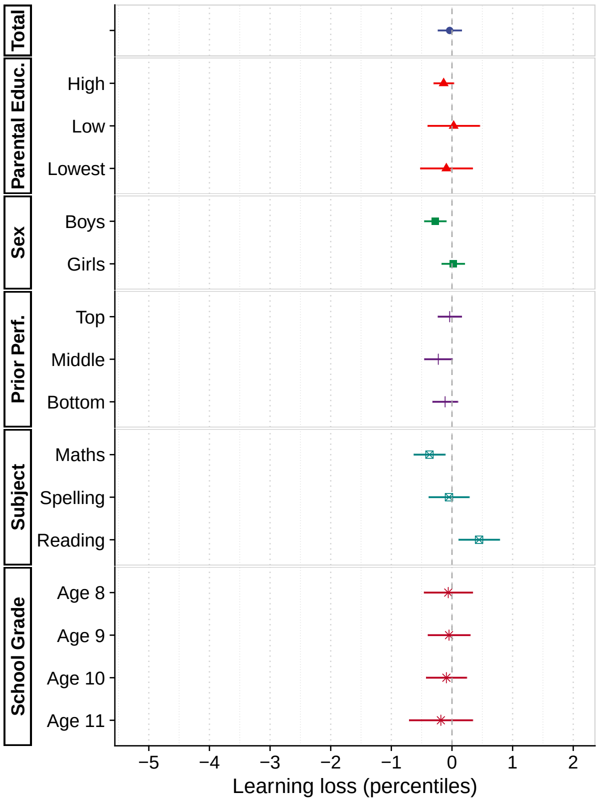

(b) 2018 as treated

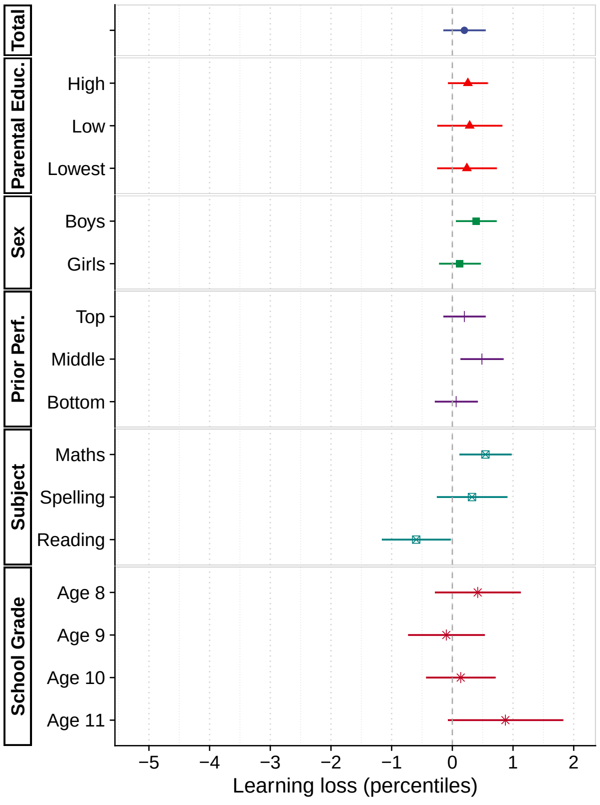

(c) 2019 as treated

Fig. S5. Placebo effects for non-treated years. The graphs show results using our main specification but excluding the actual treatment year and instead assigning treatment status to each comparison year.

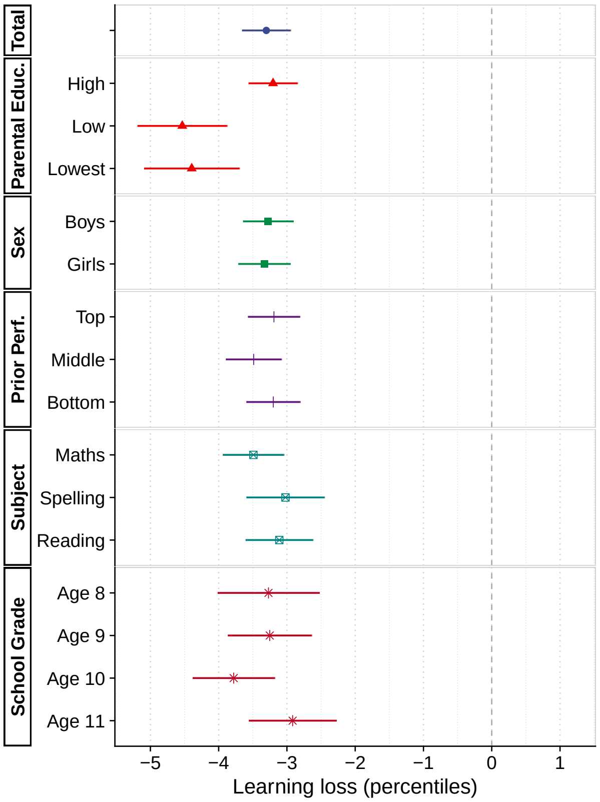

(a) 2017 excluded

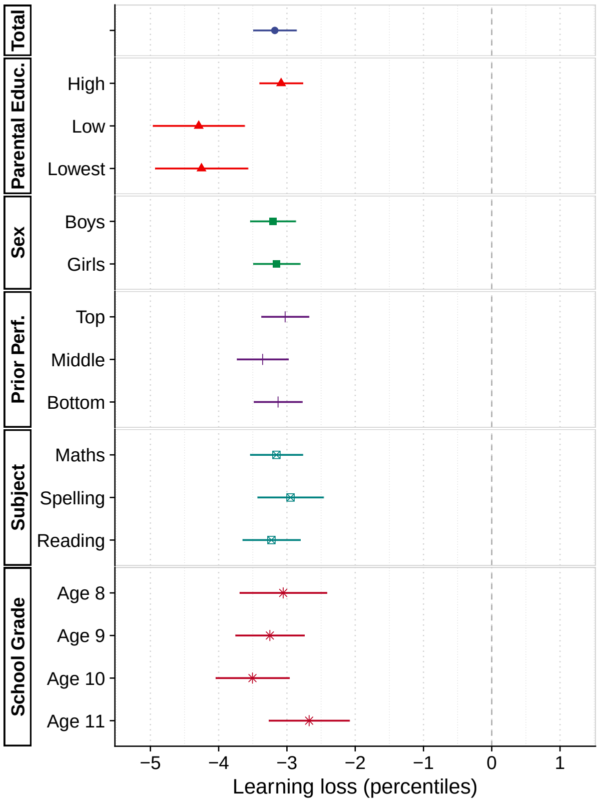

(b) 2018 excluded

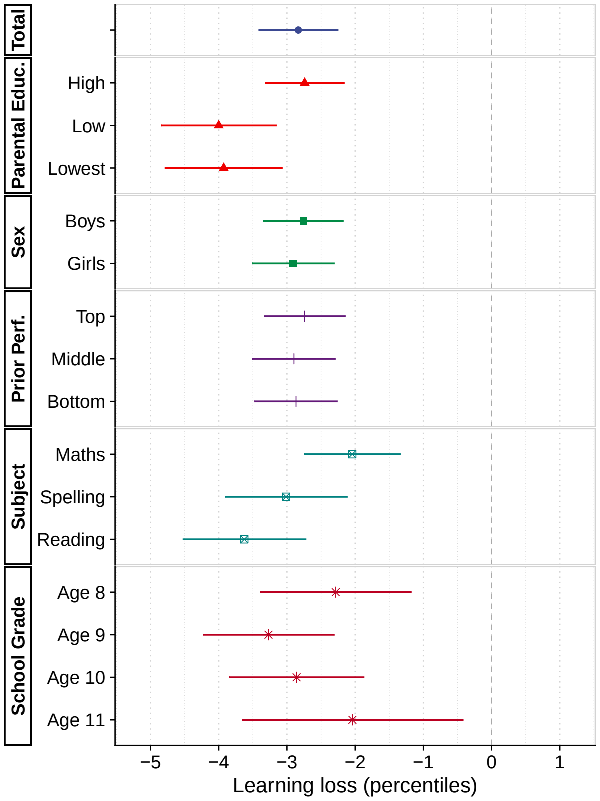

(c) 2019 excluded

Fig. S6. Robustness dropping comparison years. The graphs show results using our main specification but in turn excluding each comparison year from the analysis.

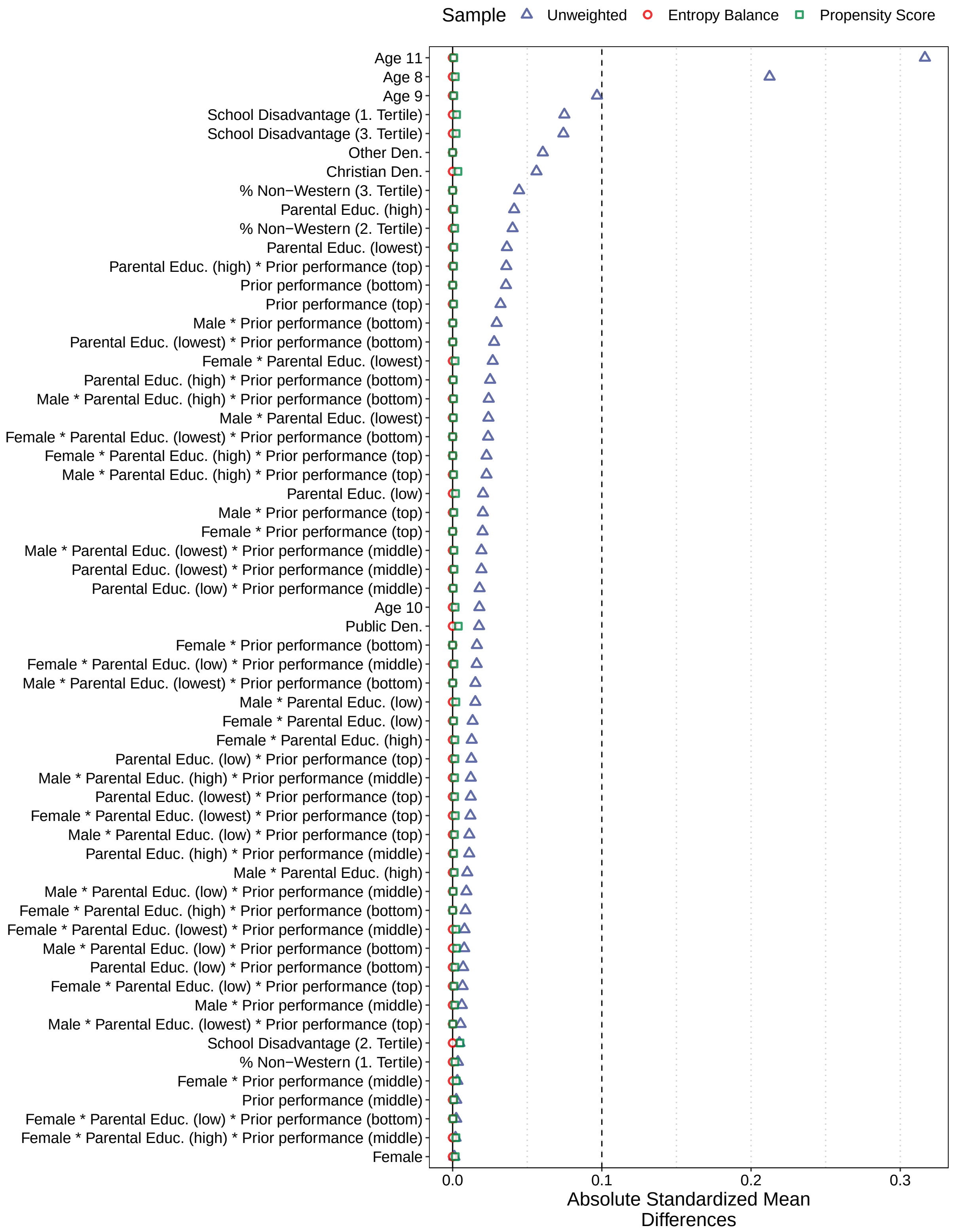

Fig. S7. Balancing plot for weighted comparisons. The graph shows absolute standardized mean differences on balancing covariates between treatment and comparison years before adjustment and after reweighting on maximum entropy weights and the estimated propensity of treatment.

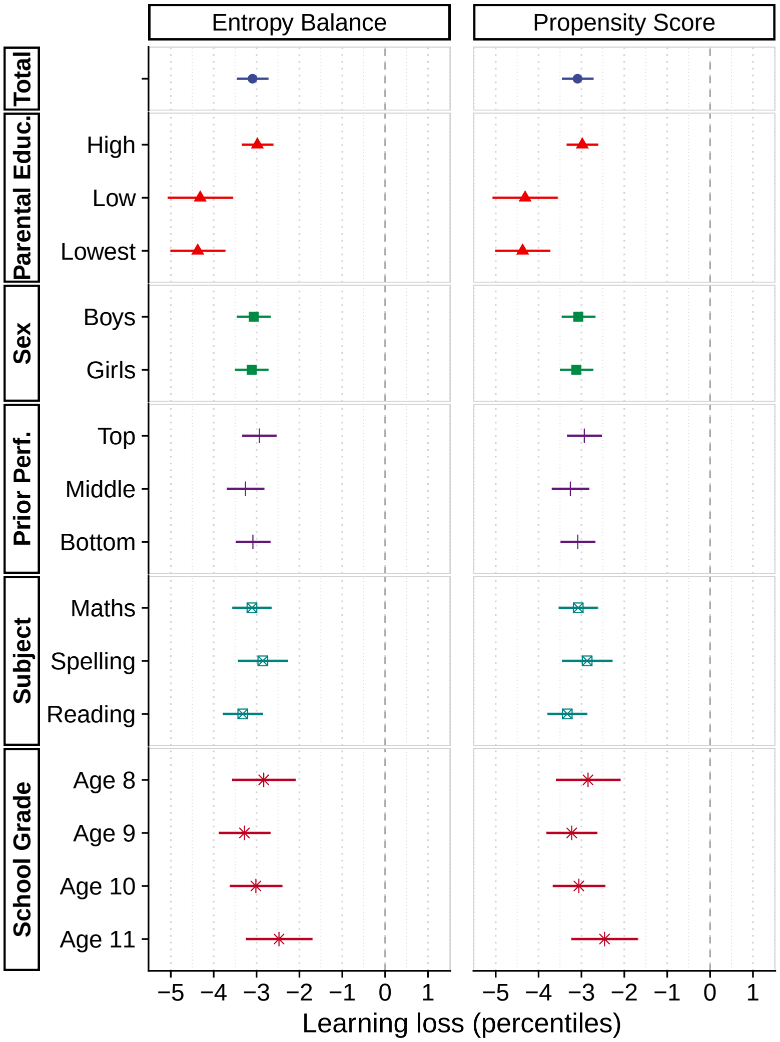

Fig. S8. Results with covariate balancing. The graph shows results using our main specification while balancing treatment and comparison years on maximum entropy weights (“E-Balance,” left) and the estimated propensity of treatment (“P-Score,” right).

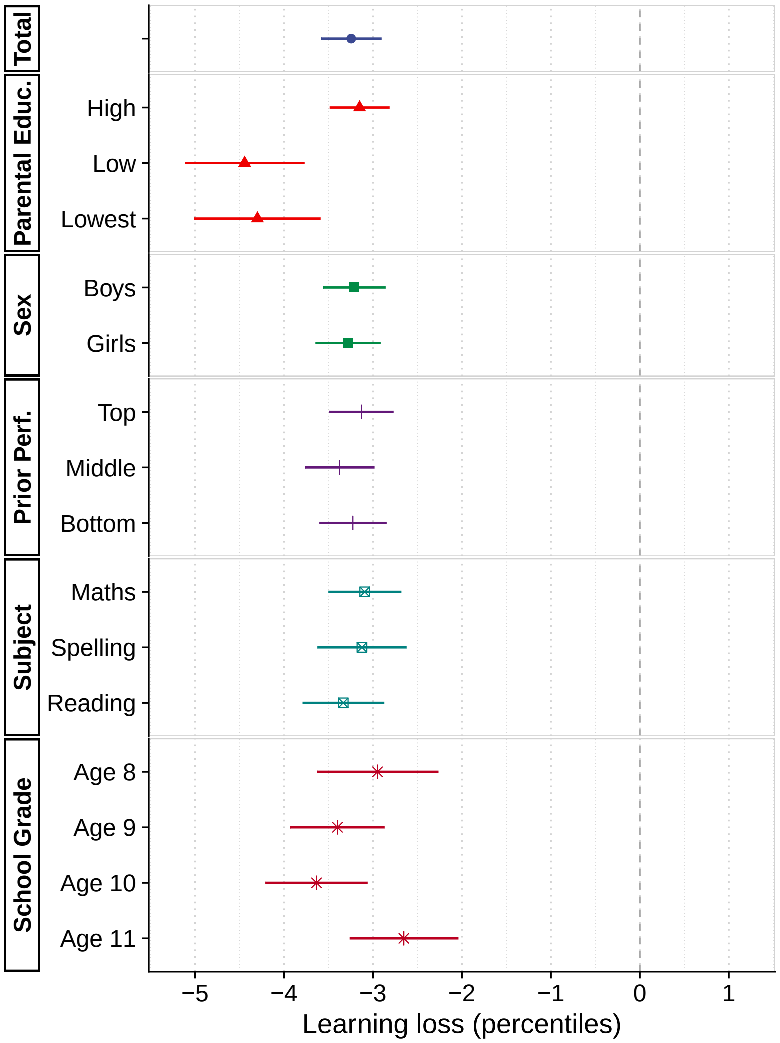

Fig. S9. Results for near-complete schools. The graph shows results from a specification identical to our main analysis except the sample is restricted to schools where at least 75% of students returned to take end-of-year tests following school closures.

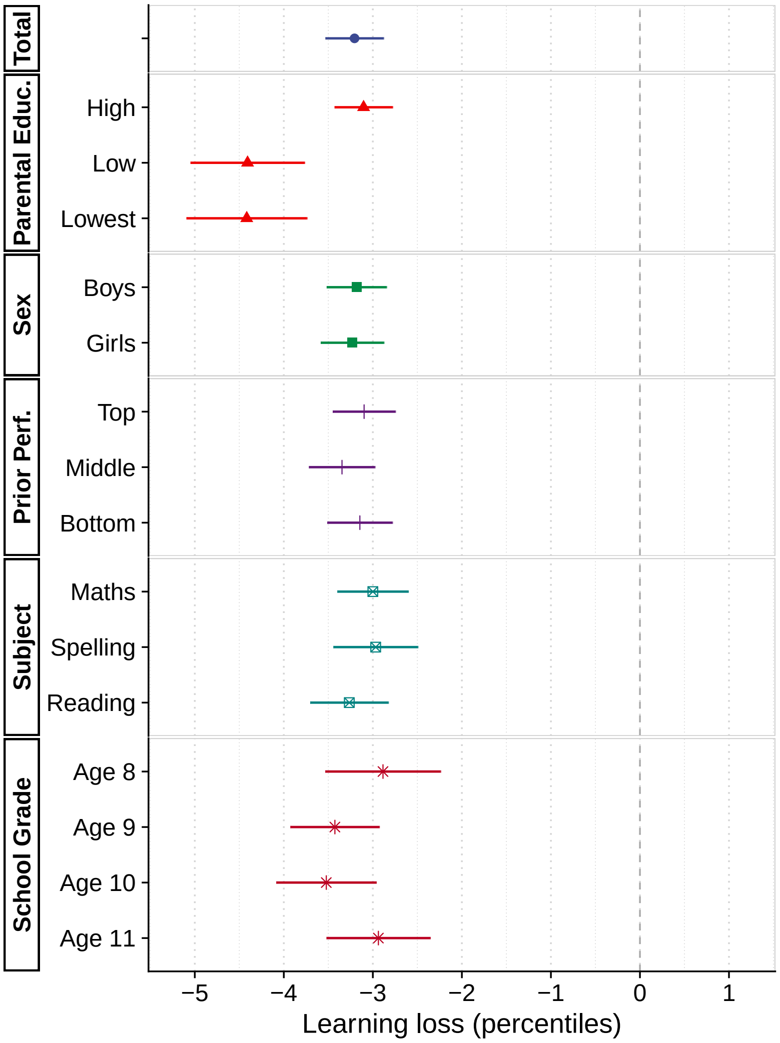

Fig. S10. School fixed effects. The graph shows results combining our difference-in-differences with school fixed effects. This analysis discards all variation between schools by introducing a separate intercept for each school, thus adjusting for any heterogeneity across schools.

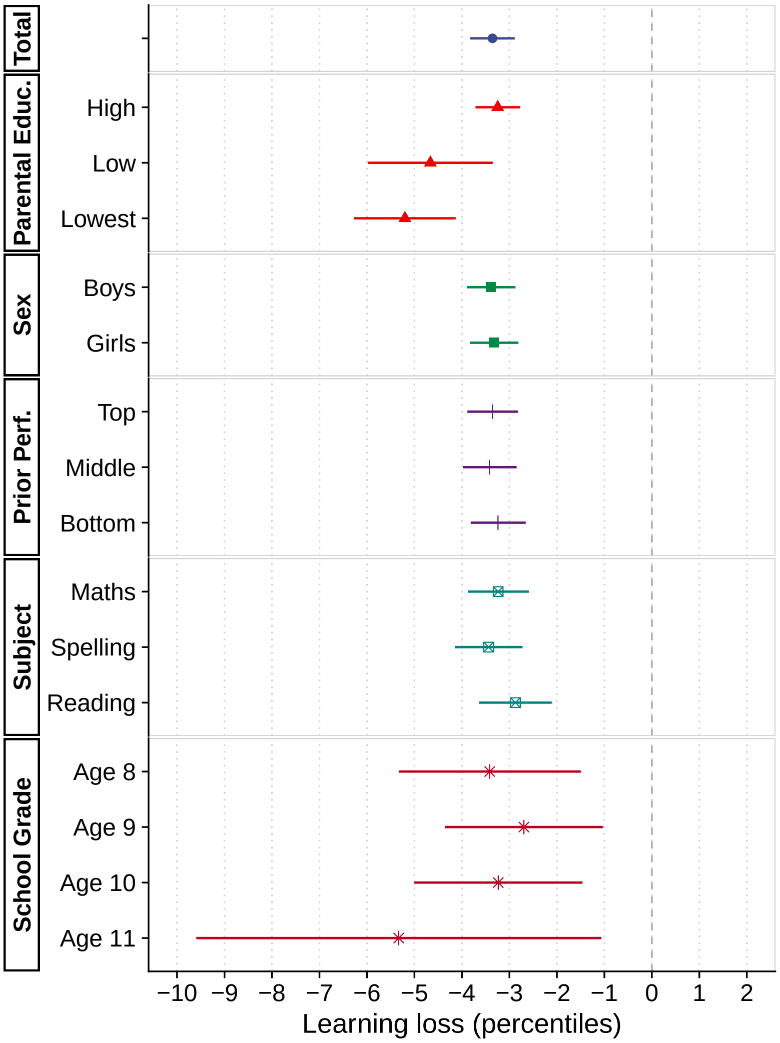

Fig. S11. Family fixed effects. The graph shows results combining our difference-in-differences with family fixed effects. This analysis discards all variation between families by introducing a separate intercept for each sibling group, thus adjusting for any heterogeneity across families.

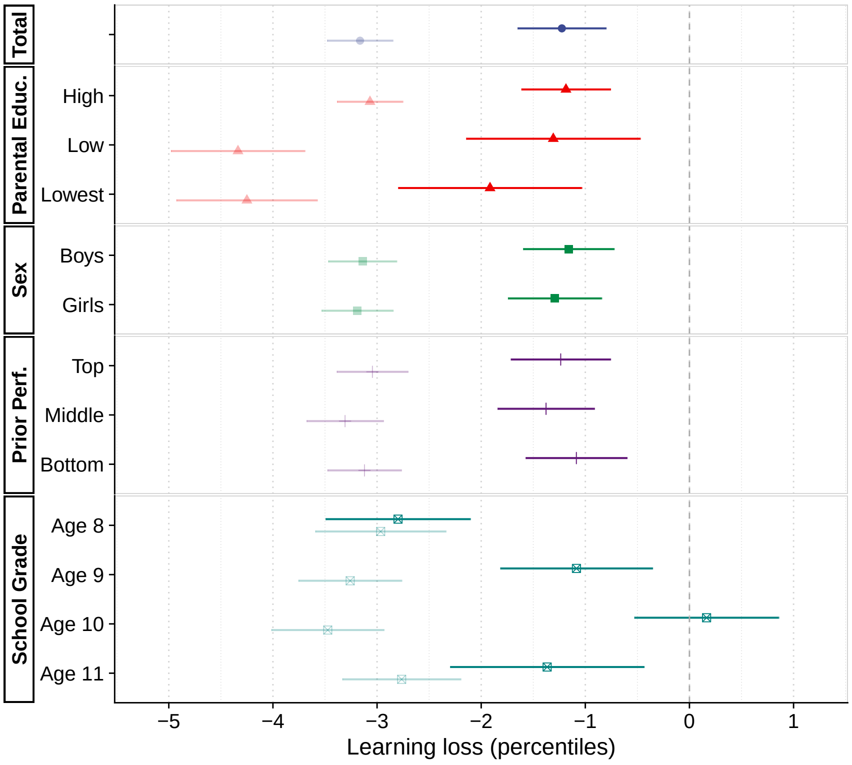

(a) Difference in treatment year

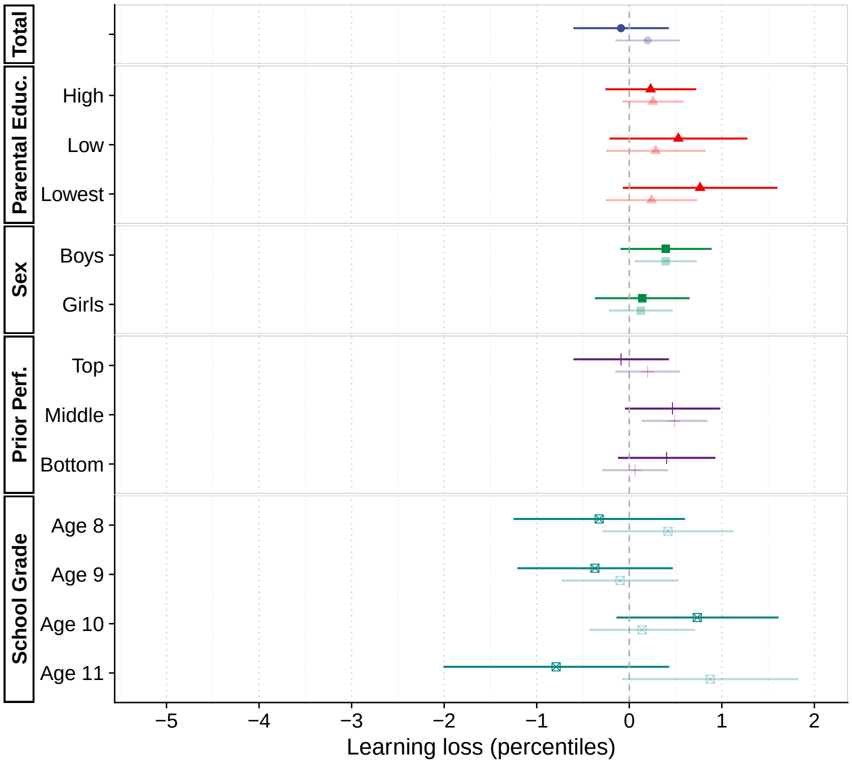

(b) Difference in placebo year (2019)

Fig. S12. Results for learning readiness. The graph contrasts results on learning readiness, in solid colors, with our composite achievement score, in transparent colors. The top panel shows estimated treatment effects for 2020, the bottom panel shows placebo results for 2019. The pooled treatment effect for learning readiness in 2020 is 62% smaller than that of our main analysis, arguably due to the fact that these tests do not assess curricular content.

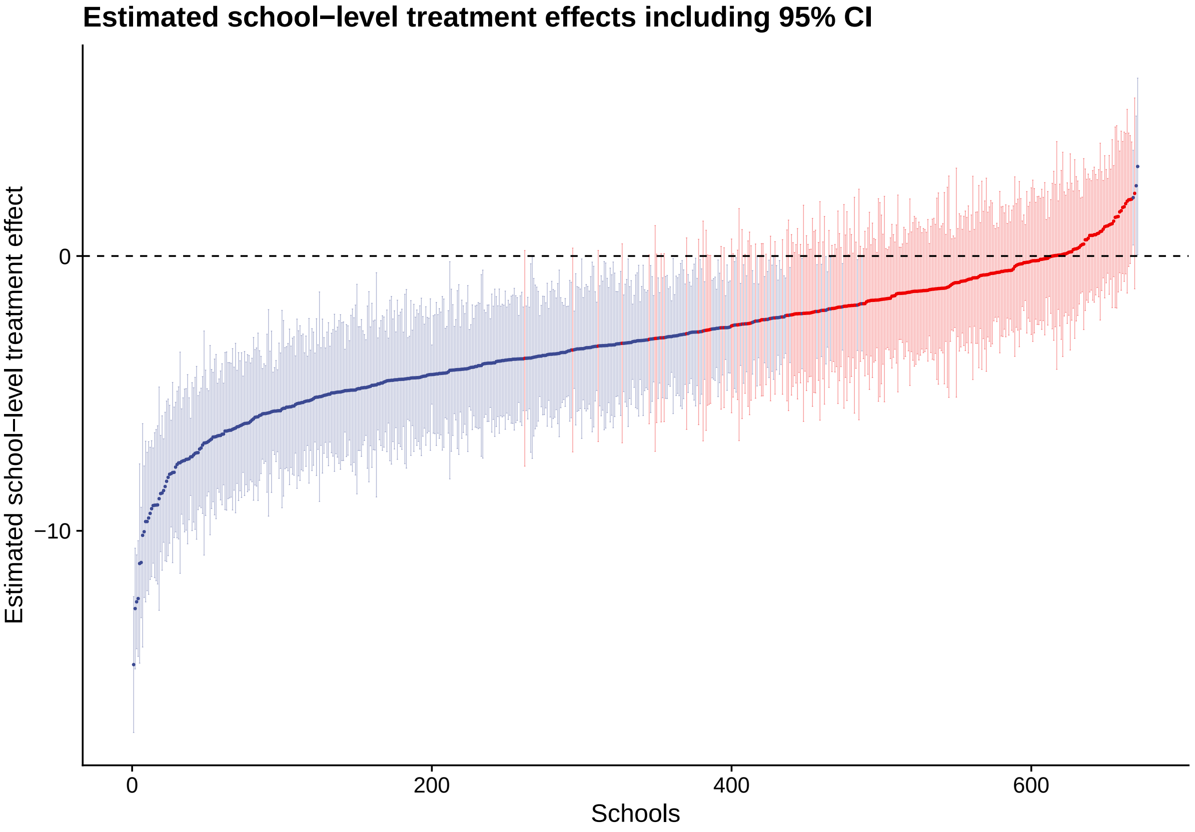

(a) School-level treatment effects

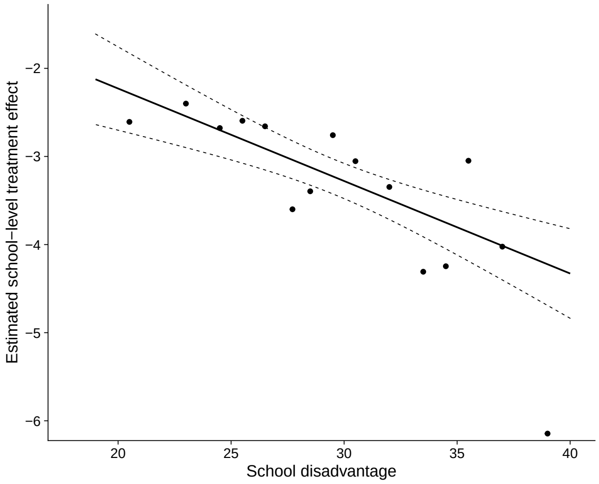

(b) School effect by SES

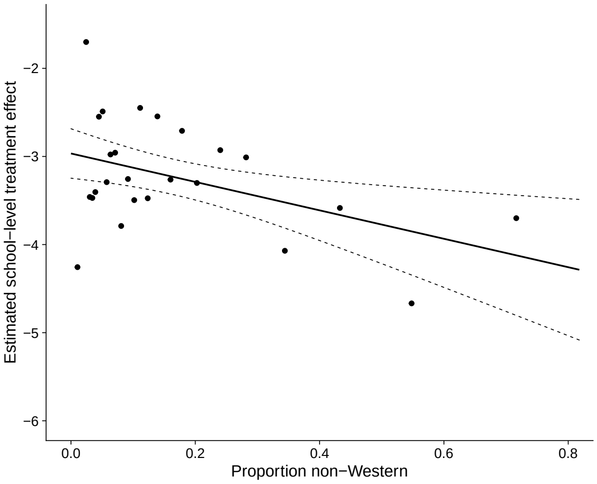

(c) School effect by proportion immigrants

Fig. S13. School-level effects. The top panel shows estimates of learning loss by school from a linear mixed model allowing learning loss to differ across schools. The bottom panels plot the predicted effects against school-level covariates: socioeconomic disadvantage and proportion non-Western immigrant background.

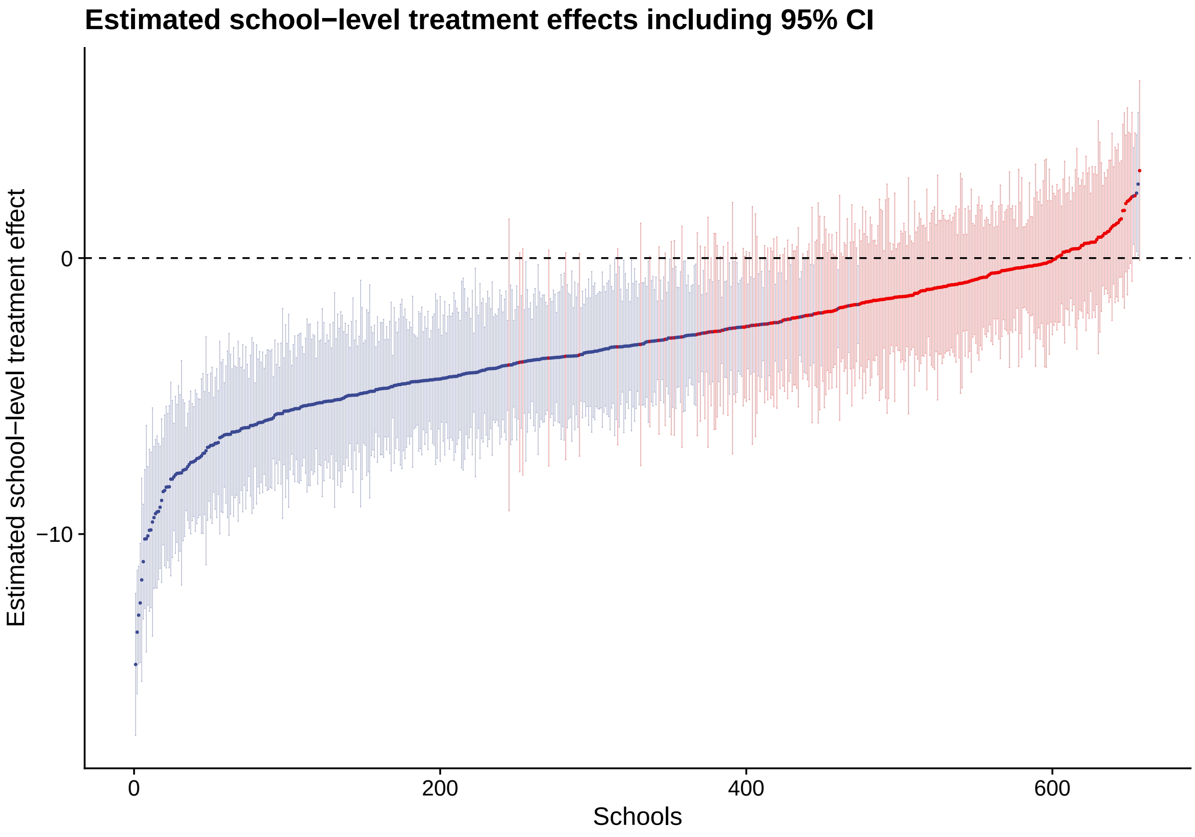

(a) School-level treatment effects

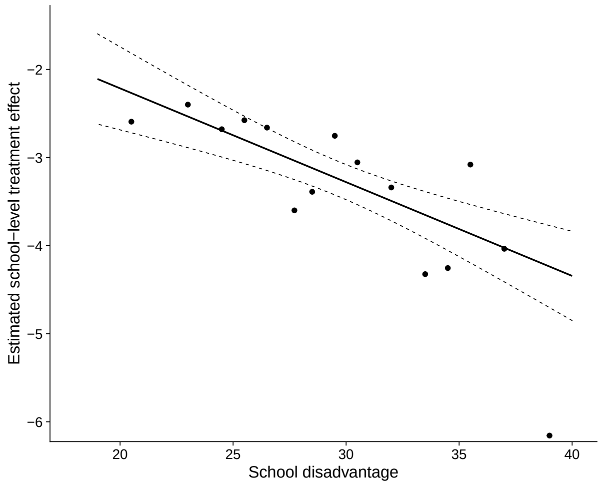

(b) School effect by SES

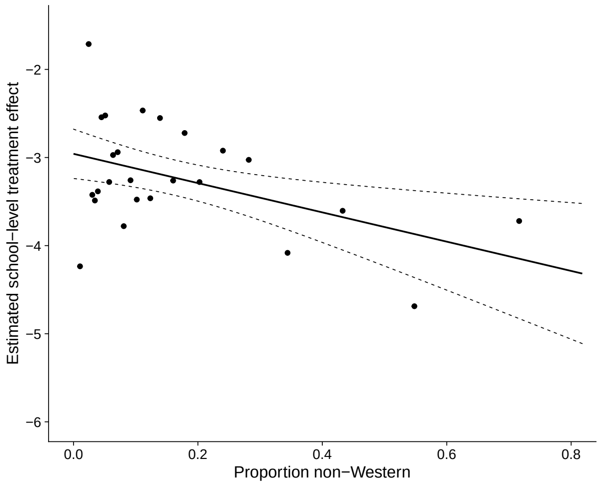

(c) School effect by proportion immigrants

Fig. S14. School-level effects with controls. The top panel shows estimates of learning loss by school, the bottom panels plot predicted effects against school-level covariates. These results are identical to those in Fig. S13 except school-level effects are adjusted for individual-level covariates: sex, parental education, and prior performance.

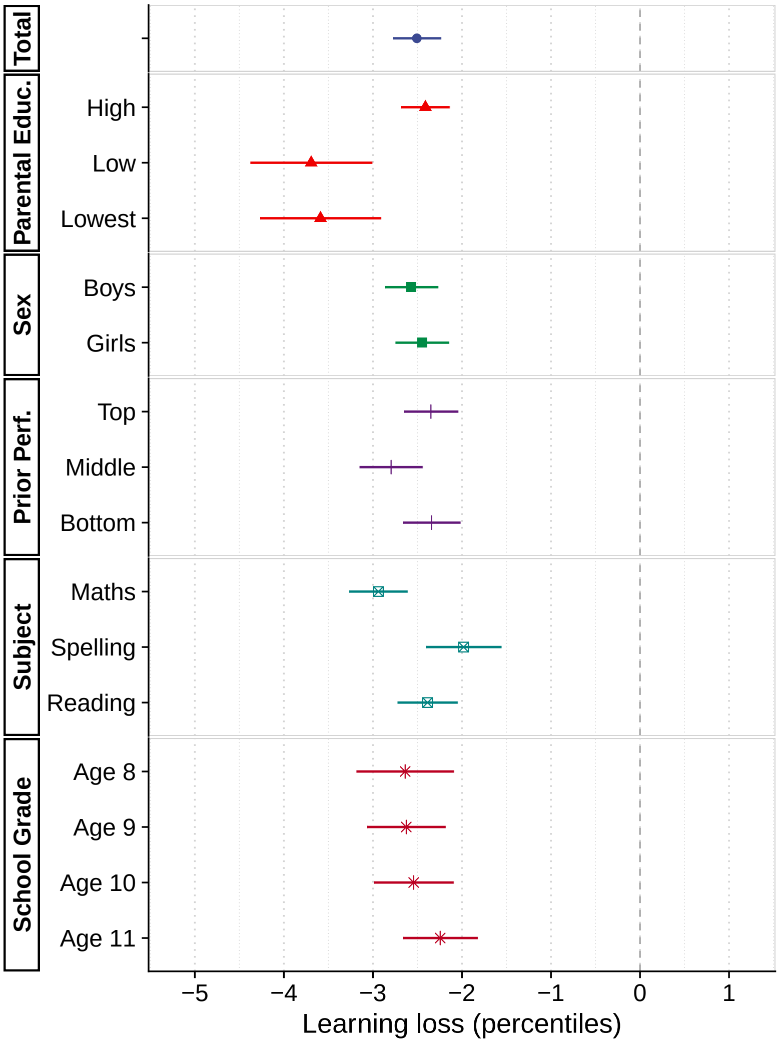

Fig. S15. Robustness to trend assumption. The graph shows results excluding the modeling of a linear time trend and using 2019 as a single comparison year.

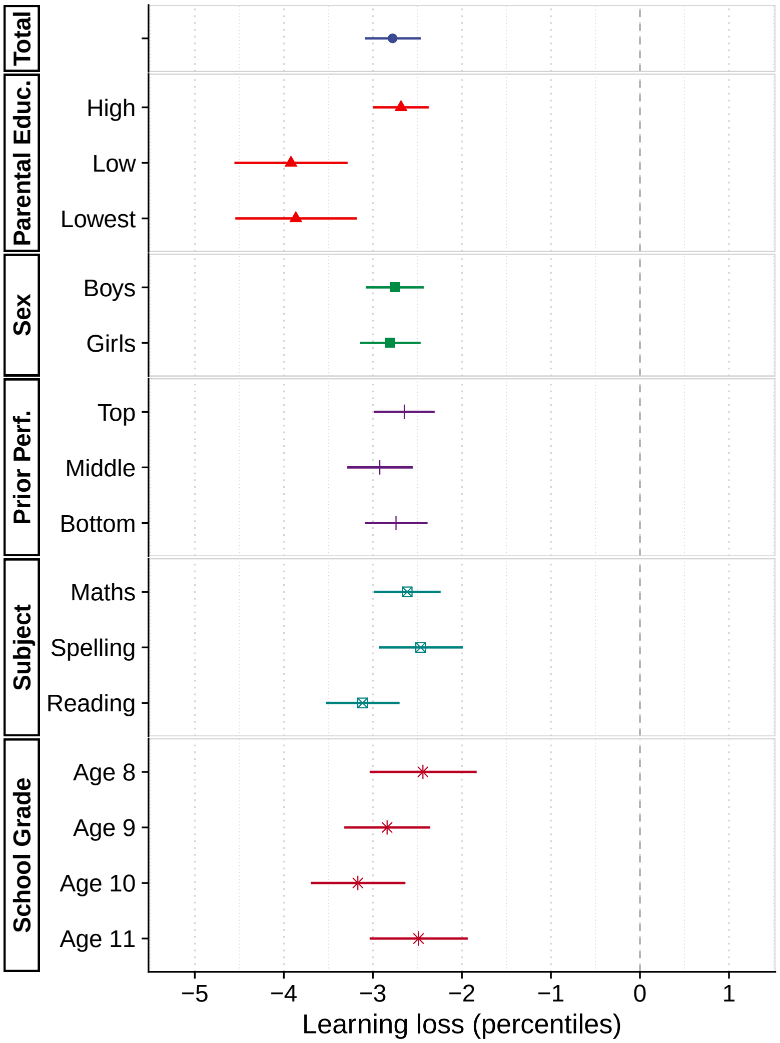

Fig. S16. Robustness excluding testing date. The graph shows results from a specification identical to our main analysis except that the number of days between tests is excluded from the covariates.

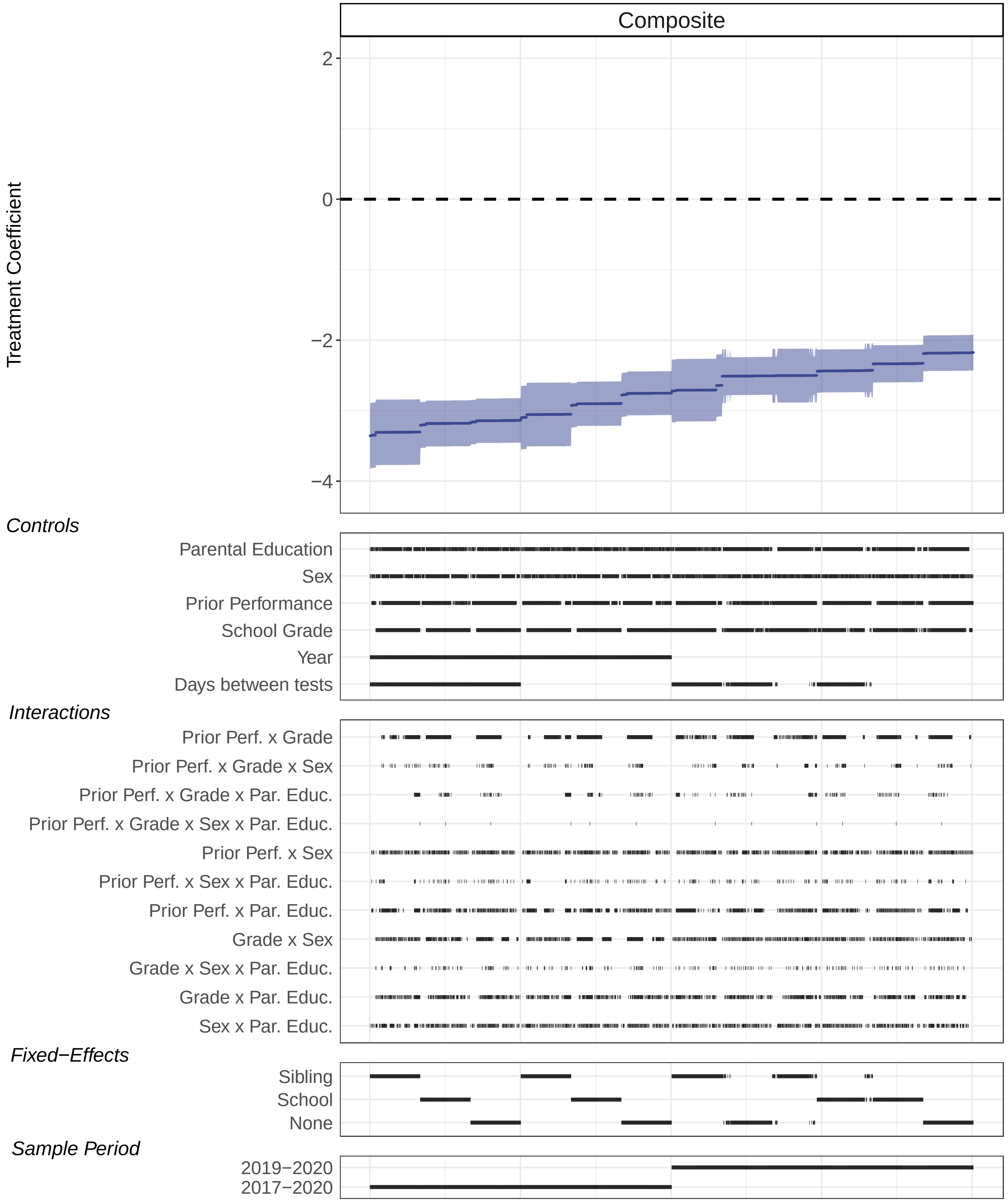

Fig. S17. Specification curve: Composite. The graph shows the estimated coefficient for learning loss in the treatment year for our Composite score across a large number of model specifications. Dots indicate the inclusion of a modelling choice into the specification. Specifications are ordered by effect size magnitude.

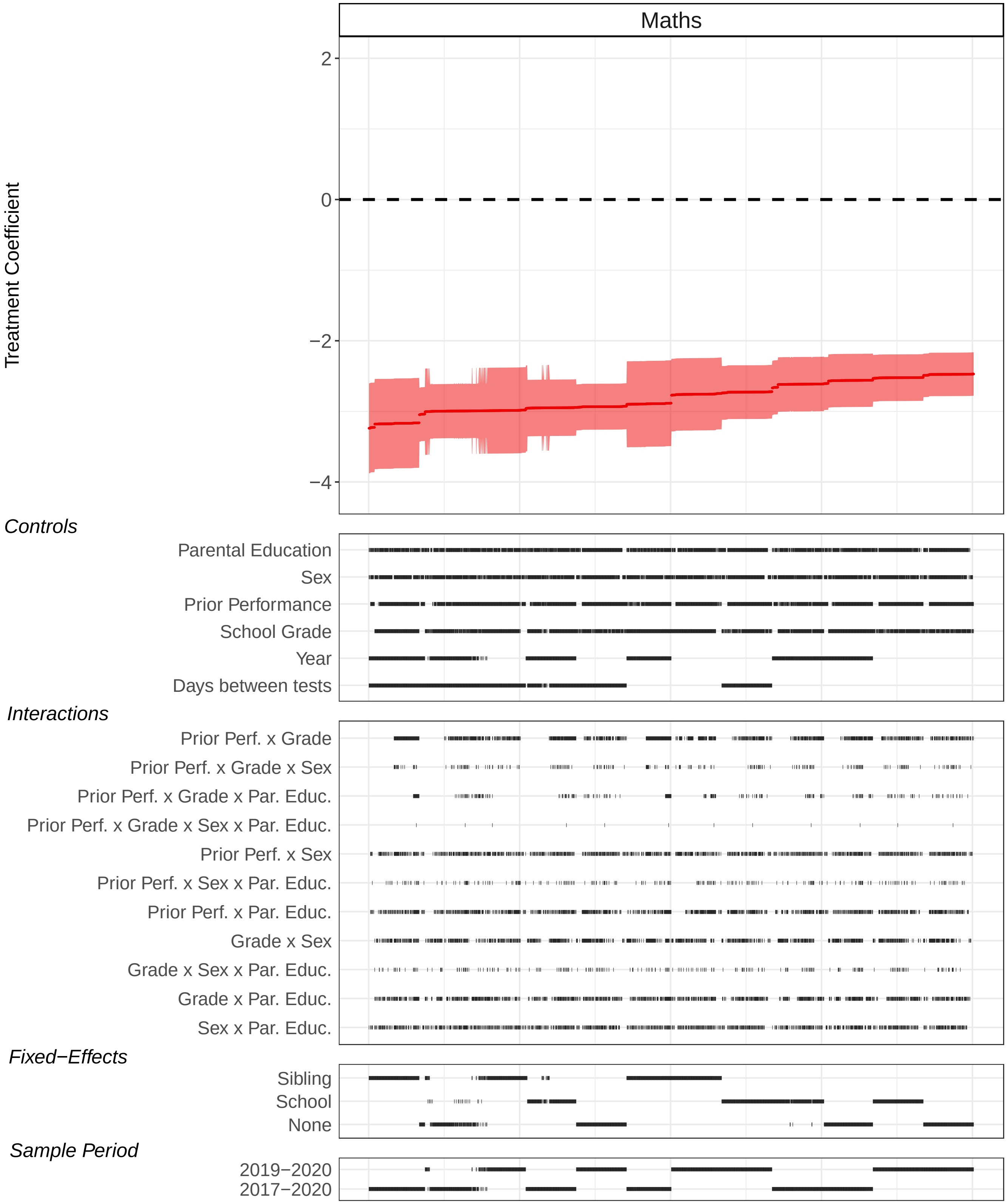

Fig. S18. Specification curve: Maths. The graph shows the estimated coefficient for learning loss in the treatment year for Maths across a large number of model specifications. Dots indicate the inclusion of a modelling choice into the specification. Specifications are ordered by effect size magnitude.

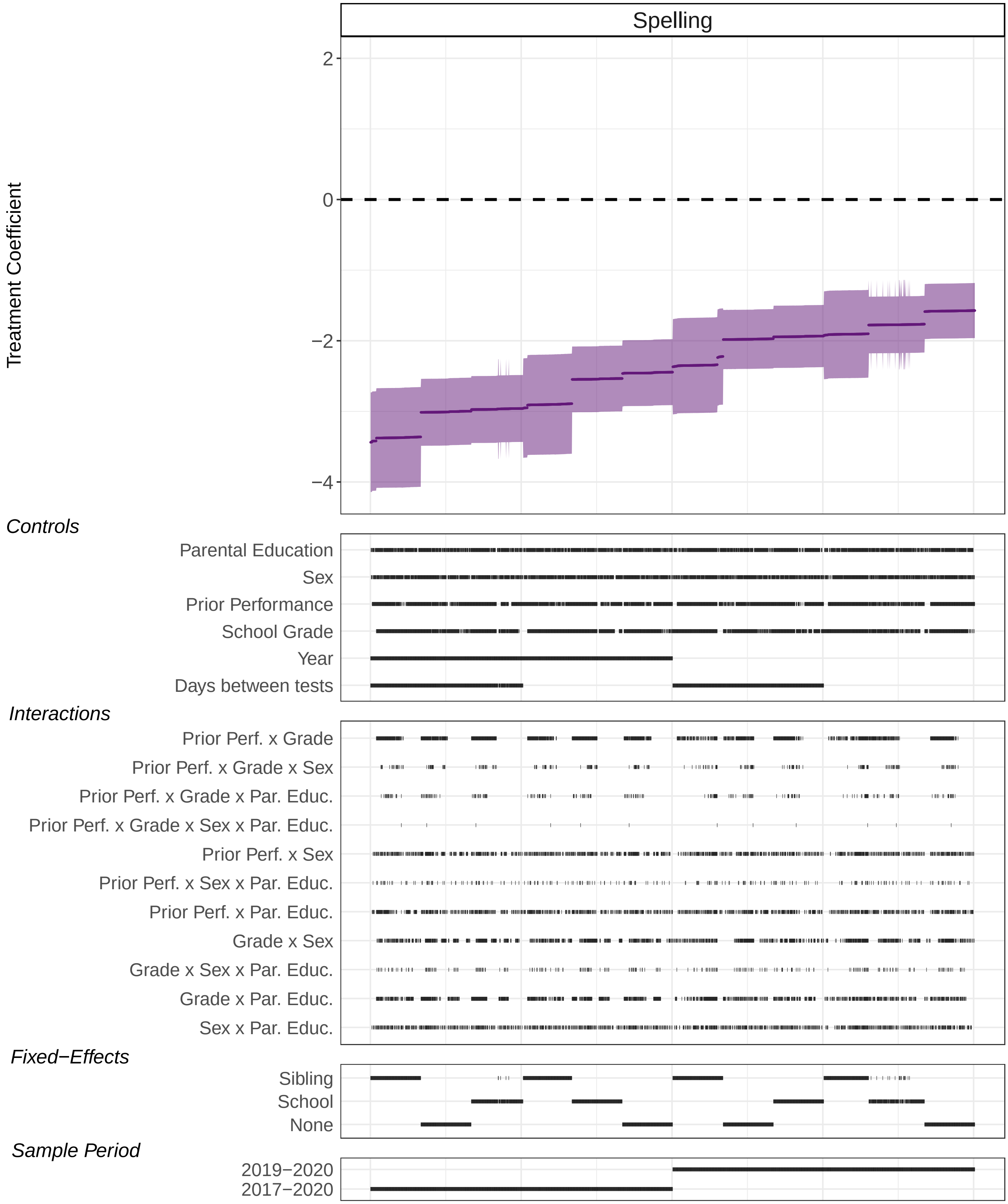

Fig. S19. Specification curve: Spelling. The graph shows the estimated coefficient for learning loss in the treatment year for Spelling across a large number of model specifications. Dots indicate the inclusion of a modelling choice into the specification. Specifications are ordered by effect size magnitude.

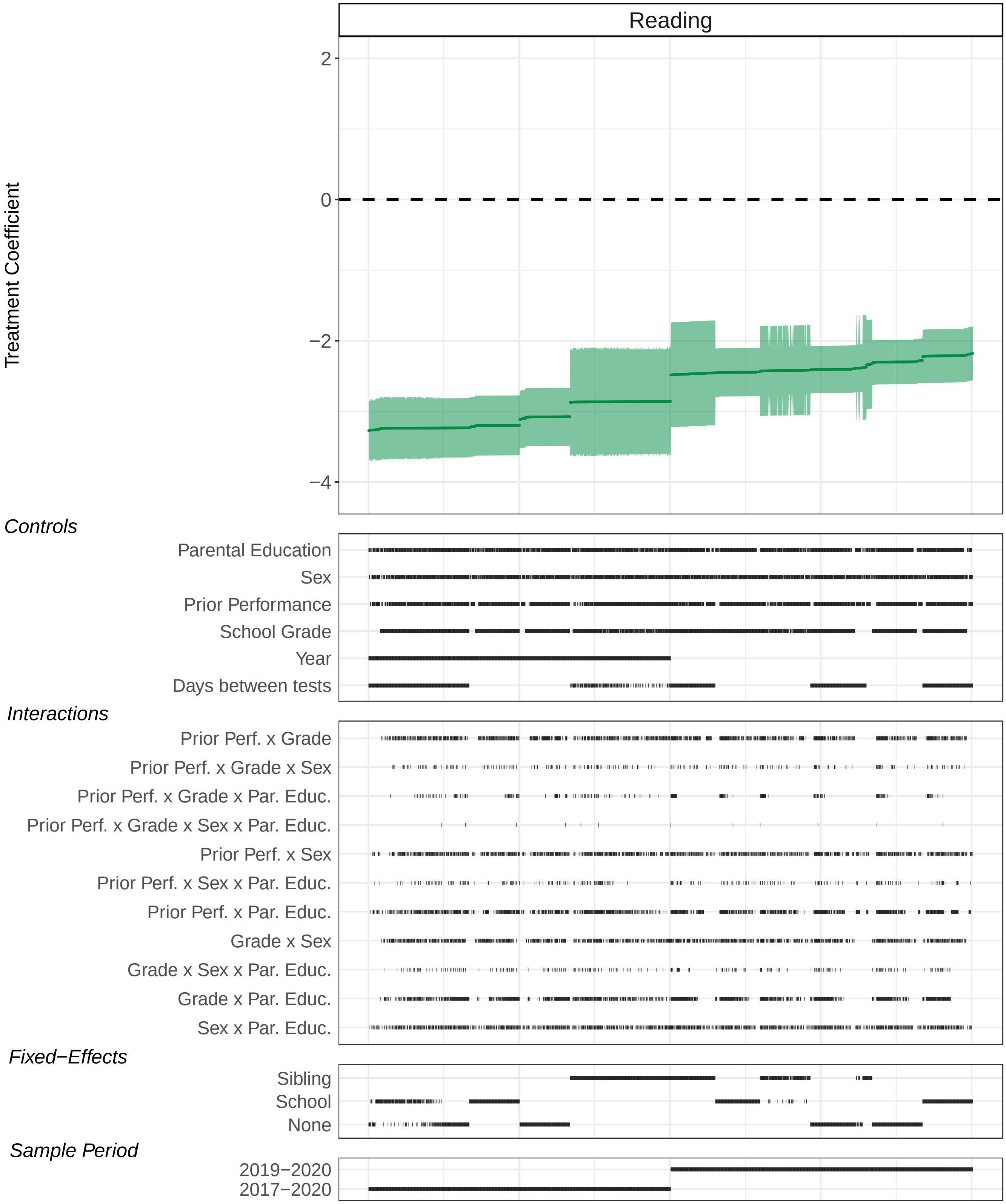

Fig. S20. Specification curve: Reading. The graph shows the estimated coefficient for learning loss in the treatment year for Reading across a large number of model specifications. Dots indicate the inclusion of a modelling choice into the specification. Specifications are ordered by effect size magnitude.

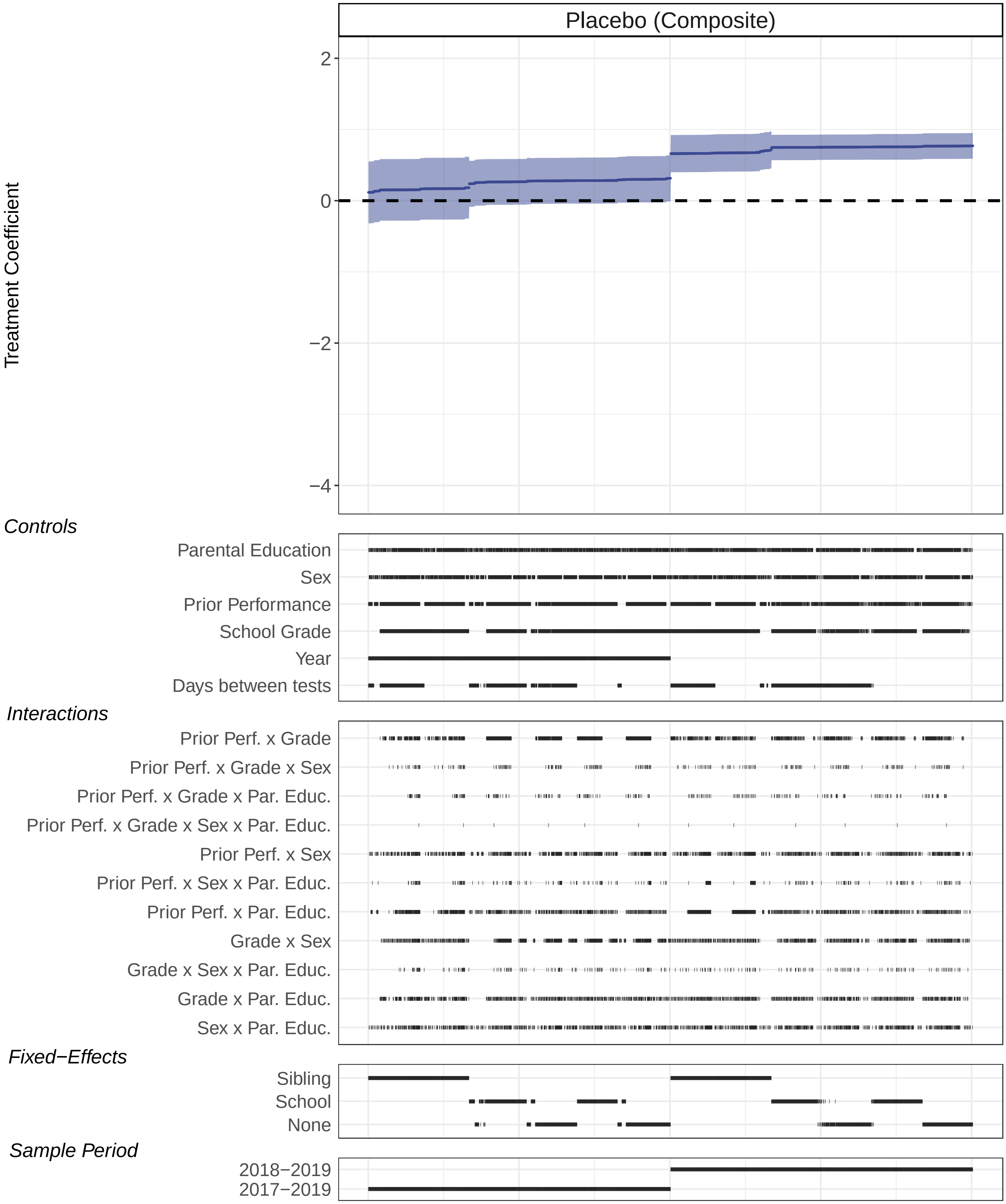

Fig. S21. Specification curve: Placebo. The graph shows the estimated coefficient for learning loss for our Composite score across a large number of model specifications using 2019 as the treatment year. Dots indicate the inclusion of a modelling choice into the specification. Specifications are ordered by effect size magnitude.

Table S1. Estimated weekly learning gains

	Composite	Maths	Reading	Spelling
All	0.32	0.34	0.16	0.42
Boys	0.30	0.32	0.17	0.37
Girls	0.34	0.36	0.16	0.47
Parental Education				
High	0.32	0.34	0.17	0.43
Low	0.33	0.31	n.s.	0.41
Lowest	0.24	0.36	n.s.	0.27
Prior Performance				
Top	0.33	0.33	0.24	0.41
Middle	0.37	0.39	0.16	0.51
Bottom	0.26	0.31	n.s.	0.35
School Grade				
Age 8	0.39	0.35	n.s.	0.63
Age 9	0.33	0.40	0.22	0.38
Age 10	0.27	0.32	0.18	0.27
Age 11	0.27	0.28	n.s.	0.41
Num. obs.	289189	286515	217875	284499

Weekly learning gains in percentiles per week, 2017–2019 sample only.
n.s.= not significantly different from zero ($p \geq 0.05$).

Table S2. Percent missing data

	2017				2018				2019				2020			
	Age 8	Age 9	Age 10	Age 11	Age 8	Age 9	Age 10	Age 11	Age 8	Age 9	Age 10	Age 11	Age 8	Age 9	Age 10	Age 11
Sex	0%	0%	0%	0%	0%	0%	0%	0%	0%	0%	0%	0%	0%	0%	0%	0%
Parental education	0%	0%	0%	0%	0%	0%	0%	0%	0%	0%	0%	0%	0%	0%	0%	0%
Prior performance	6%	6%	5%	6%	7%	6%	5%	5%	6%	6%	5%	5%	6%	6%	5%	4%
School ID	0%	0%	0%	0%	0%	0%	0%	0%	0%	0%	0%	0%	0%	0%	0%	0%
School denomination	4%	4%	4%	6%	4%	4%	4%	4%	4%	4%	4%	4%	4%	4%	4%	4%
School disadvantage	4%	4%	4%	6%	4%	4%	4%	4%	4%	4%	4%	4%	4%	4%	4%	4%
Proportion non-Western	4%	4%	4%	6%	4%	4%	4%	4%	4%	4%	4%	4%	4%	4%	4%	4%
Sibling ID	40%	45%	47%	93%	36%	40%	45%	47%	40%	37%	40%	45%	45%	40%	38%	41%
Composite																
Mid-year	2%	2%	1%	1%	2%	2%	2%	1%	2%	2%	1%	1%	2%	2%	2%	2%
End-of-year	1%	1%	1%	3%	0%	0%	1%	3%	2%	1%	1%	3%	35%	35%	35%	34%
Difference	2%	2%	2%	4%	2%	2%	2%	4%	3%	3%	3%	4%	36%	36%	36%	35%
Maths																
Mid-year	2%	2%	2%	2%	2%	2%	2%	2%	2%	2%	2%	2%	3%	3%	3%	2%
End-of-year	1%	1%	1%	4%	1%	1%	1%	5%	2%	2%	2%	4%	38%	38%	38%	36%
Difference	3%	3%	3%	5%	2%	3%	3%	6%	3%	3%	4%	6%	39%	39%	39%	37%
Reading																
Mid-year	14%	3%	2%	5%	15%	3%	3%	3%	18%	3%	3%	3%	22%	3%	3%	3%
End-of-year	5%	30%	60%	99%	5%	12%	23%	53%	6%	8%	12%	22%	49%	47%	47%	44%
Difference	15%	31%	60%	99%	16%	14%	25%	54%	20%	10%	13%	24%	57%	48%	48%	45%
Spelling																
Mid-year	2%	2%	5%	2%	2%	2%	2%	4%	2%	2%	2%	3%	2%	2%	2%	3%
End-of-year	1%	1%	4%	4%	1%	1%	1%	7%	2%	2%	2%	6%	45%	45%	45%	45%
Difference	3%	3%	5%	5%	2%	3%	3%	9%	4%	4%	4%	8%	45%	46%	45%	46%
Learning readiness																
Mid-year	3%	5%	7%	7%	4%	6%	8%	10%	3%	6%	8%	11%	4%	6%	10%	14%
End-of-year	2%	4%	6%	7%	2%	5%	8%	11%	3%	6%	9%	13%	35%	44%	49%	54%
Difference	4%	7%	9%	10%	5%	8%	10%	13%	5%	9%	12%	16%	36%	46%	51%	56%
Num. obs.	26834	27368	26196	5536	26859	27932	28385	27017	28609	27993	28953	29212	28061	28191	27770	28516

Table S3. Summary statistics

	Control			Treated			
Variable	N	Mean	SD	N	Mean	SD	*p*-value
Δ Composite	289189	0.28	10.99	69190	–1.28	11.83	$<$0.001 (F)
Δ Maths	286515	0.36	15.00	66114	–1.79	15.21	$<$0.001 (F)
Δ Reading	217875	0.66	18.96	54487	–1.01	18.94	$<$0.001 (F)
Δ Spelling	284499	0.05	17.27	58627	–0.71	17.07	$<$0.001 (F)
Days between tests	289189	135.99	10.74	69190	145.16	9.96	$<$0.001 (F)
Parental Education	289189			69190			$<$0.001 (χ^2)
High		0.91			0.92		
Low		0.04			0.04		
Lowest		0.05			0.04		
Sex	289189			69190			0.727 (χ^2)
Boys		0.50			0.50		
Girls		0.50			0.50		
Prior Performance	289189			69190			$<$0.001 (χ^2)
Top		0.33			0.34		
Middle		0.34			0.34		
Bottom		0.33			0.32		
School Grade	289189			69190			$<$0.001 (χ^2)
Age 8		0.26			0.25		
Age 9		0.27			0.25		
Age 10		0.27			0.25		
Age 11		0.20			0.26		
School Disadvantage	278153	29.03	4.44	66090	28.71	4.52	$<$0.001 (F)
Proportion non-Western	278153	0.17	0.17	66090	0.17	0.17	$<$0.001 (F)
School Denomination	278153			66090			$<$0.001 (χ^2)
Christian		0.57			0.54		
Public		0.32			0.33		
Other		0.12			0.13		

Table S4. Correlation between prior and current performance

	Prior perf.	Mid-year test	End-of-year	Difference	Num. obs.
Prior performance	1.00				
Mid-year test	0.86	1.00			
End-of-year test	0.83	0.89	1.00		
Difference	–0.04	–0.20	0.24	1.00	358379
Control years					
Prior performance	1.00				
Mid-year test	0.85	1.00			
End-of-year test	0.83	0.89	1.00		
Difference	–0.04	–0.20	0.23	1.00	289189
Treatment year					
Prior performance	1.00				
Mid-year test	0.87	1.00			
End-of-year test	0.82	0.88	1.00		
Difference	–0.03	–0.18	0.25	1.00	69190

Correlation (Pearson's *r*) between composite performance in previous year and outcomes in study year: composite mid-year, end-of-year, and difference scores.

Table S5. Immigrant status by parental education

Parental education	Native (%)	Immigrant (%)	Total
High	986 756 (77)	302 927 (23)	1 289 683
Low	36 758 (65)	19 601 (35)	56 359
Lowest	6 551 (12)	49 254 (88)	55 805
Total	1 030 065 (73)	371 782 (27)	1 401 847

Population data from Statistics Netherlands, 2018–2019 school year.
Source: CBS Statline (https://opendata.cbs.nl/statline).

Table S6. Performance by test, year, and student characteristics

	Composite			Maths			Reading			Spelling			Learning readiness		
	Mid-year	End-year	Difference	Mid-year	End-year	Difference	Mid-year	End-year	Difference	Mid-year	End-year	Difference	Mid-year	End-year	Difference
**2017**															
All	52.56	52.08	-0.32	52.30	52.50	0.26	53.30	52.94	-0.30	52.39	51.48	-0.84	54.28	53.65	-0.44
Boys	52.45	52.27	-0.44	57.32	57.24	-0.03	49.77	49.66	0.24	50.34	49.17	-1.10	54.11	53.21	-0.69
Girls	52.67	51.90	-0.20	47.29	47.77	0.54	56.81	56.23	-0.85	54.43	53.79	-0.58	54.45	54.08	-0.19
Parental education															
High	54.23	53.69	-0.31	54.10	54.33	0.28	55.37	54.91	-0.21	53.51	52.54	-0.91	54.91	54.41	-0.33
Low	39.23	38.95	-0.66	37.82	37.24	-0.59	37.77	38.00	-0.75	42.31	41.77	-0.53	46.74	45.26	-1.21
Lowest	35.74	36.29	-0.18	34.10	34.52	0.56	31.40	29.75	-1.69	42.10	42.02	0.10	50.15	48.09	-1.58
Prior performance															
Top	75.57	74.61	-0.90	75.75	75.68	-0.05	75.90	74.03	-0.60	75.25	73.42	-1.84	71.98	71.18	-0.74
Middle	53.22	52.59	-0.46	52.93	53.15	0.21	54.28	52.99	-0.48	52.51	51.43	-1.08	53.98	53.53	-0.35
Bottom	29.97	30.28	0.29	29.02	29.67	0.58	30.66	31.04	0.08	30.41	30.67	0.21	37.90	37.59	-0.26
**2018**															
All	51.79	51.79	0.18	51.88	51.84	0.12	52.08	52.19	0.61	51.73	51.63	0.04	52.83	52.85	0.35
Boys	51.98	51.98	-0.06	56.97	56.54	-0.27	48.96	49.21	0.88	50.11	49.53	-0.45	52.94	52.72	0.09
Girls	51.61	51.60	0.42	46.81	47.15	0.50	55.19	55.17	0.35	53.35	53.73	0.53	52.71	52.98	0.60
Parental education															
High	53.35	53.31	0.18	53.54	53.51	0.10	54.03	54.10	0.68	52.78	52.65	-0.01	53.45	53.55	0.39
Low	37.84	38.11	0.04	36.41	36.55	0.06	35.63	36.38	0.13	41.57	41.53	0.16	45.20	44.94	0.07
Lowest	35.20	35.82	0.38	34.41	34.48	0.42	30.46	29.73	-0.22	41.28	41.80	0.93	48.00	47.16	-0.28
Prior performance															
Top	75.21	74.83	-0.32	75.78	75.42	-0.35	75.21	74.81	0.49	74.75	73.94	-0.79	70.48	70.34	-0.02
Middle	52.25	52.26	0.08	52.37	52.45	0.08	52.78	52.47	0.49	51.71	51.65	-0.07	52.51	52.64	0.27
Bottom	28.64	29.27	0.64	27.80	28.40	0.59	28.83	29.35	0.78	29.45	30.13	0.71	36.24	36.78	0.70
**2019**															
All	50.89	51.75	0.95	50.88	51.53	0.71	50.99	51.92	1.32	51.18	52.10	0.97	51.39	52.51	1.37
Boys	51.14	52.06	0.91	56.12	56.51	0.43	47.70	49.07	1.85	49.77	50.42	0.68	51.51	52.58	1.26
Girls	50.64	51.44	1.00	45.60	46.53	0.99	54.31	54.77	0.78	52.60	53.78	1.25	51.26	52.44	1.48
Parental education															
High	52.41	53.27	0.95	52.47	53.10	0.69	52.87	53.82	1.38	52.25	53.16	0.94	52.04	53.21	1.41
Low	36.48	37.17	0.70	35.29	36.08	0.71	34.30	34.85	0.73	40.18	40.85	0.82	43.60	44.43	1.02
Lowest	34.07	35.11	1.18	33.51	34.47	1.18	29.17	29.27	0.56	39.94	41.24	1.65	45.64	46.30	0.86
Prior performance															
Top	74.94	75.19	0.35	75.33	75.44	0.14	74.96	74.98	0.77	74.67	74.98	0.32	69.60	70.15	0.66
Middle	51.40	52.40	1.07	51.39	52.25	0.86	51.62	52.48	1.35	51.26	52.38	1.14	51.37	52.75	1.56
Bottom	27.35	28.69	1.28	26.53	27.69	1.09	27.23	28.82	1.71	28.53	29.74	1.16	34.31	35.95	1.76
**2020**															
All	50.81	49.64	-1.24	50.71	49.26	-1.78	50.56	49.90	-0.97	51.60	51.12	-0.62	50.71	48.89	1.08
Boys	51.32	50.37	-1.35	56.28	54.57	-2.10	47.46	47.25	-0.49	50.44	49.50	-1.00	51.08	49.16	0.92
Girls	50.30	48.90	-1.12	45.08	43.93	-1.45	53.69	52.57	-1.46	52.78	52.74	-0.24	50.33	48.61	1.24
Parental education															
High	52.21	51.13	-1.14	52.18	50.85	-1.67	52.29	51.72	-0.85	52.61	52.15	-0.55	51.34	49.48	1.15
Low	35.82	32.82	-2.72	34.31	30.64	-3.10	32.75	30.60	-2.62	40.51	38.90	-2.19	42.31	41.37	0.57
Lowest	33.73	30.50	-2.10	33.29	29.36	-3.00	28.86	26.38	-2.19	39.38	37.32	-0.98	44.73	43.26	0.11
Prior performance															
Top	74.78	73.51	-1.66	75.10	73.17	-2.41	74.55	73.80	-1.16	74.88	74.00	-0.91	68.83	67.75	0.63
Middle	50.99	49.61	-1.41	50.96	49.15	-1.91	50.60	49.63	-1.15	51.57	50.46	-0.78	50.64	48.99	0.94
Bottom	26.61	25.53	-0.74	25.55	24.39	-0.96	26.24	25.53	-0.70	28.27	27.60	-0.39	33.59	32.96	1.47

Table S7. Main effects by subject

	Composite	Maths	Reading	Spelling
Treatment	-3.16^{***}	-3.04^{***}	-3.27^{***}	-3.01^{***}
	(0.16)	(0.19)	(0.21)	(0.24)
Year (std.)	0.69^{***}	0.26^{***}	0.86^{***}	0.96^{***}
	(0.06)	(0.07)	(0.09)	(0.09)
Days between tests (std.)	0.48^{***}	0.54^{***}	0.20^{**}	0.65^{***}
	(0.05)	(0.05)	(0.07)	(0.09)
(Intercept)	0.68^{***}	0.57^{***}	1.02^{***}	0.60^{***}
	(0.07)	(0.07)	(0.08)	(0.12)
R^2	0.01	0.00	0.00	0.00
Adj. R^2	0.01	0.00	0.00	0.00
Num. obs.	358379	352629	272362	343126
RMSE	11.14	15.03	18.95	17.21
N Clusters	937	937	930	936

$^{***}p < 0.001$; $^{**}p < 0.01$; $^{*}p < 0.05$

Table S8. Results by parental education and subject

	Composite	Maths	Reading	Spelling
Treatment	-3.07^{***}	-2.93^{***}	-3.21^{***}	-2.90^{***}
	(0.16)	(0.19)	(0.22)	(0.24)
Treat x Par. Educ. (low)	-1.27^{***}	-1.10^{**}	-1.14^{*}	-1.73^{***}
	(0.29)	(0.39)	(0.48)	(0.45)
Treat x Par. Educ. (lowest)	-1.18^{***}	-1.79^{***}	-0.37	-1.29^{*}
	(0.33)	(0.42)	(0.43)	(0.51)
Parental Educ. (low)	-0.26^{*}	-0.30	-0.61^{**}	0.14
	(0.12)	(0.16)	(0.21)	(0.20)
Parental Educ. (lowest)	0.22	0.42^{*}	-1.02^{***}	0.92^{**}
	(0.16)	(0.17)	(0.20)	(0.29)
Year (std.)	0.69^{***}	0.26^{***}	0.86^{***}	0.97^{***}
	(0.06)	(0.07)	(0.09)	(0.09)
Days between tests (std.)	0.48^{***}	0.54^{***}	0.20^{**}	0.65^{***}
	(0.05)	(0.05)	(0.07)	(0.09)
(Intercept)	0.68^{***}	0.56^{***}	1.09^{***}	0.55^{***}
	(0.07)	(0.07)	(0.08)	(0.12)
R^2	0.01	0.00	0.00	0.00
Adj. R^2	0.01	0.00	0.00	0.00
Num. obs.	358379	352629	272362	343126
RMSE	11.14	15.03	18.95	17.21
N Clusters	937	937	930	936

$^{***}p < 0.001$; $^{**}p < 0.01$; $^{*}p < 0.05$

Table S9. Results by student sex and subject

	Composite	Maths	Reading	Spelling
Treatment	-3.14^{***}	-3.05^{***}	-3.24^{***}	-3.04^{***}
	(0.17)	(0.20)	(0.23)	(0.26)
Treat x Female	-0.05	0.01	-0.05	0.05
	(0.12)	(0.15)	(0.19)	(0.18)
Female	0.26^{***}	0.65^{***}	-0.91^{***}	0.70^{***}
	(0.04)	(0.06)	(0.08)	(0.07)
Year (std.)	0.69^{***}	0.26^{***}	0.86^{***}	0.96^{***}
	(0.06)	(0.07)	(0.09)	(0.09)
Days between tests (std.)	0.48^{***}	0.54^{***}	0.20^{**}	0.65^{***}
	(0.05)	(0.05)	(0.07)	(0.09)
(Intercept)	0.54^{***}	0.24^{**}	1.47^{***}	0.26^{*}
	(0.07)	(0.08)	(0.09)	(0.12)
R^2	0.01	0.00	0.00	0.00
Adj. R^2	0.01	0.00	0.00	0.00
Num. obs.	358379	352629	272362	343126
RMSE	11.14	15.02	18.94	17.21
N Clusters	937	937	930	936

$^{***}p < 0.001$; $^{**}p < 0.01$; $^{*}p < 0.05$

Table S10. Results by prior performance and subject

	Composite	Maths	Reading	Spelling
Treatment	-3.04^{***}	-3.24^{***}	-3.12^{***}	-2.51^{***}
	(0.17)	(0.21)	(0.24)	(0.28)
Treat x Prior Perf. (middle)	-0.26^{*}	0.02	-0.27	-0.62^{**}
	(0.13)	(0.18)	(0.23)	(0.21)
Treat x Prior Perf. (bottom)	-0.08	0.61^{**}	-0.19	-0.87^{***}
	(0.16)	(0.21)	(0.24)	(0.24)
Prior Perf. (middle)	0.53^{***}	0.50^{***}	0.27^{*}	0.78^{***}
	(0.06)	(0.08)	(0.11)	(0.10)
Prior Perf. (bottom)	1.02^{***}	0.87^{***}	0.65^{***}	1.42^{***}
	(0.07)	(0.09)	(0.11)	(0.11)
Year (std.)	0.69^{***}	0.26^{***}	0.86^{***}	0.96^{***}
	(0.06)	(0.07)	(0.09)	(0.09)
Days between tests (std.)	0.49^{***}	0.55^{***}	0.20^{**}	0.65^{***}
	(0.05)	(0.05)	(0.07)	(0.09)
(Intercept)	0.16^{*}	0.11	0.71^{***}	-0.12
	(0.07)	(0.08)	(0.10)	(0.13)
R^2	0.01	0.01	0.00	0.00
Adj. R^2	0.01	0.01	0.00	0.00
Num. obs.	358379	352629	272362	343126
RMSE	11.13	15.02	18.95	17.20
N Clusters	937	937	930	936

$^{***}p < 0.001$; $^{**}p < 0.01$; $^{*}p < 0.05$

Table S11. Results by grade and subject

	Composite	Maths	Reading	Spelling
**Age 8**				
Treatment	-2.97^{***}	-2.52^{***}	-2.29^{***}	-3.75^{***}
	(0.32)	(0.40)	(0.43)	(0.51)
Year (std.)	0.42^{***}	0.22	0.57^{***}	0.58^{**}
	(0.11)	(0.15)	(0.16)	(0.18)
Days between tests (std.)	0.62^{***}	0.64^{***}	0.15	0.99^{***}
	(0.10)	(0.12)	(0.14)	(0.19)
(Intercept)	0.42^{***}	0.22	0.80^{***}	0.39
	(0.13)	(0.16)	(0.17)	(0.23)
R^2	0.01	0.00	0.00	0.00
Adj. R^2	0.01	0.00	0.00	0.00
Num. obs.	93439	92180	76397	90403
RMSE	12.45	17.23	20.92	19.89
N Clusters	935	935	870	934
**Age 9**				
Treatment	-3.26^{***}	-2.93^{***}	-3.90^{***}	-2.83^{***}
	(0.25)	(0.34)	(0.41)	(0.38)
Year (std.)	0.67^{***}	0.40^{***}	0.85^{***}	0.75^{***}
	(0.09)	(0.12)	(0.17)	(0.14)
Days between tests (std.)	0.52^{***}	0.60^{***}	0.39^{**}	0.61^{***}
	(0.07)	(0.09)	(0.12)	(0.12)
(Intercept)	0.65^{***}	0.41^{***}	0.82^{***}	0.79^{***}
	(0.09)	(0.11)	(0.15)	(0.16)
R^2	0.01	0.00	0.00	0.00
Adj. R^2	0.01	0.00	0.00	0.00
Num. obs.	94747	93417	79016	91567
RMSE	10.84	15.17	19.37	15.90
N Clusters	936	936	912	935
**Age 10**				
Treatment	-3.47^{***}	-3.74^{***}	-3.37^{***}	-3.02^{***}
	(0.28)	(0.32)	(0.40)	(0.44)
Year (std.)	0.99^{***}	0.40^{***}	1.13^{***}	1.42^{***}
	(0.10)	(0.11)	(0.16)	(0.17)
Days between tests (std.)	0.40^{***}	0.49^{***}	0.23^{*}	0.39^{**}
	(0.07)	(0.08)	(0.10)	(0.15)
(Intercept)	0.88^{***}	0.82^{***}	1.22^{***}	0.76^{***}
	(0.10)	(0.11)	(0.13)	(0.20)
R^2	0.01	0.01	0.00	0.00
Adj. R^2	0.01	0.01	0.00	0.00
Num. obs.	95364	93769	68412	91315
RMSE	10.41	13.79	16.90	16.40
N Clusters	936	936	887	931
**Age 11**				
Treatment	-2.76^{***}	-2.01^{***}	-2.97^{***}	-3.03^{***}
	(0.29)	(0.34)	(0.51)	(0.48)
Year (std.)	0.51^{**}	-0.82^{***}	0.47	1.60^{***}
	(0.15)	(0.17)	(0.33)	(0.26)
Days between tests (std.)	0.38^{***}	0.46^{***}	-0.03	0.57^{***}
	(0.09)	(0.10)	(0.15)	(0.15)
(Intercept)	0.77^{***}	0.88^{***}	1.33^{***}	0.43^{*}
	(0.11)	(0.12)	(0.16)	(0.18)
R^2	0.01	0.01	0.00	0.00
Adj. R^2	0.01	0.01	0.00	0.00
Num. obs.	74829	73263	48537	69841
RMSE	10.66	13.27	17.64	16.08
N Clusters	931	925	834	920

$^{***}p < 0.001$; $^{**}p < 0.01$; $^{*}p < 0.05$

Table S12. Main effects with controls

	Composite	Maths	Reading	Spelling
Treatment	-3.14^{***}	-3.00^{***}	-3.24^{***}	-3.00^{***}
	(0.16)	(0.20)	(0.21)	(0.24)
Female	0.26^{***}	0.65^{***}	-0.91^{***}	0.71^{***}
	(0.04)	(0.06)	(0.07)	(0.07)
Parental Educ. (low)	-0.74^{***}	-0.76^{***}	-0.99^{***}	-0.43^{*}
	(0.11)	(0.15)	(0.19)	(0.18)
Parental Educ. (lowest)	-0.31^{*}	-0.21	-1.32^{***}	0.35
	(0.13)	(0.15)	(0.19)	(0.26)
Prior Perf. (middle)	0.50^{***}	0.51^{***}	0.26^{**}	0.67^{***}
	(0.05)	(0.07)	(0.10)	(0.09)
Prior Perf. (bottom)	1.05^{***}	1.01^{***}	0.75^{***}	1.27^{***}
	(0.06)	(0.07)	(0.10)	(0.10)
Year (std.)	0.67^{***}	0.22^{**}	0.82^{***}	0.96^{***}
	(0.06)	(0.07)	(0.09)	(0.09)
Days between tests (std.)	0.49^{***}	0.55^{***}	0.20^{**}	0.66^{***}
	(0.05)	(0.05)	(0.07)	(0.09)
(Intercept)	-0.42	-1.28^{***}	0.70^{*}	-0.40
	(0.23)	(0.31)	(0.35)	(0.40)
R^2	0.01	0.01	0.00	0.00
Adj. R^2	0.01	0.01	0.00	0.00
Num. obs.	358379	352629	272362	343126
RMSE	11.13	15.02	18.94	17.20
N Clusters	937	937	930	936

$^{***}p < 0.001$; $^{**}p < 0.01$; $^{*}p < 0.05$

Table S13. Main effects, complete subject scores only

	Composite	Maths	Reading	Spelling
Treatment	-3.08^{***}	-3.13^{***}	-3.35^{***}	-2.78^{***}
	(0.18)	(0.22)	(0.22)	(0.28)
Year (std.)	0.68^{***}	0.42^{***}	0.89^{***}	0.73^{***}
	(0.07)	(0.09)	(0.09)	(0.11)
Days between tests (std.)	0.50^{***}	0.56^{***}	0.25^{***}	0.69^{***}
	(0.05)	(0.06)	(0.07)	(0.10)
(Intercept)	0.80^{***}	0.53^{***}	1.03^{***}	0.84^{***}
	(0.07)	(0.08)	(0.08)	(0.13)
R^2	0.01	0.00	0.00	0.00
Adj. R^2	0.01	0.00	0.00	0.00
Num. obs.	259189	259189	259189	259189
RMSE	10.64	15.12	18.95	17.21
N Clusters	929	929	929	929

$^{***}p < 0.001$; $^{**}p < 0.01$; $^{*}p < 0.05$

Table S14. Main effects in near-complete schools

	Composite	Maths	Reading	Spelling
Treatment	-3.24^{***}	-3.09^{***}	-3.33^{***}	-3.12^{***}
	(0.17)	(0.21)	(0.23)	(0.26)
Year (std.)	0.71^{***}	0.28^{***}	0.86^{***}	1.02^{***}
	(0.07)	(0.08)	(0.10)	(0.10)
Days between tests (std.)	0.48^{***}	0.54^{***}	0.22^{**}	0.62^{***}
	(0.06)	(0.06)	(0.08)	(0.10)
(Intercept)	0.72^{***}	0.59^{***}	1.07^{***}	0.66^{***}
	(0.07)	(0.08)	(0.09)	(0.13)
R^2	0.01	0.00	0.00	0.00
Adj. R^2	0.01	0.00	0.00	0.00
Num. obs.	302587	297285	231224	288247
RMSE	11.16	15.02	18.93	17.19
N Clusters	746	746	742	745

$^{***}p < 0.001$; $^{**}p < 0.01$; $^{*}p < 0.05$

Table S15. Social inequality in near-complete schools

	Composite	Maths	Reading	Spelling
Treatment	-3.15^{***}	-2.98^{***}	-3.28^{***}	-3.01^{***}
	(0.17)	(0.21)	(0.24)	(0.26)
Treat x Par. Educ. (low)	-1.29^{***}	-1.05^{**}	-1.12^{*}	-1.83^{***}
	(0.29)	(0.40)	(0.50)	(0.46)
Treat x Par. Educ. (lowest)	-1.15^{***}	-1.74^{***}	-0.32	-1.31^{*}
	(0.34)	(0.43)	(0.44)	(0.53)
Parental Educ. (low)	-0.24	-0.35	-0.66^{**}	0.22
	(0.13)	(0.18)	(0.24)	(0.22)
Parental Educ. (lowest)	0.20	0.37^{*}	-1.05^{***}	0.97^{**}
	(0.18)	(0.18)	(0.23)	(0.34)
Year (std.)	0.71^{***}	0.28^{***}	0.85^{***}	1.03^{***}
	(0.07)	(0.08)	(0.10)	(0.10)
Days between tests (std.)	0.48^{***}	0.54^{***}	0.22^{**}	0.62^{***}
	(0.06)	(0.06)	(0.08)	(0.10)
(Intercept)	0.73^{***}	0.58^{***}	1.15^{***}	0.60^{***}
	(0.07)	(0.08)	(0.09)	(0.13)
R^2	0.01	0.01	0.00	0.00
Adj. R^2	0.01	0.00	0.00	0.00
Num. obs.	302587	297285	231224	288247
RMSE	11.16	15.02	18.93	17.19
N Clusters	746	746	742	745

$^{***}p < 0.001$; $^{**}p < 0.01$; $^{*}p < 0.05$

Table S16. Main effects with school fixed effects

	Composite	Maths	Reading	Spelling
Treatment	-3.21^{***}	-3.00^{***}	-3.26^{***}	-2.97^{***}
	(0.17)	(0.20)	(0.22)	(0.24)
Year (std.)	0.69^{***}	0.27^{***}	0.85^{***}	0.96^{***}
	(0.06)	(0.07)	(0.09)	(0.09)
Days between tests (std.)	0.37^{***}	0.44^{***}	0.05	0.53^{***}
	(0.06)	(0.07)	(0.09)	(0.09)
R^2	0.03	0.02	0.01	0.03
Adj. R^2	0.02	0.01	0.01	0.03
Num. obs.	358379	352629	272362	343126
RMSE	11.04	14.96	18.87	16.98
N Clusters	937	937	930	936

$^{***}p < 0.001$; $^{**}p < 0.01$; $^{*}p < 0.05$

Table S17. Social inequality with school fixed effects

	Composite	Maths	Reading	Spelling
Treatment	-3.10^{***}	-2.89^{***}	-3.21^{***}	-2.83^{***}
	(0.17)	(0.20)	(0.23)	(0.24)
Treat x Par. Educ. (low)	-1.30^{***}	-1.05^{**}	-0.94^{*}	-1.94^{***}
	(0.28)	(0.39)	(0.48)	(0.45)
Treat x Par. Educ. (lowest)	-1.31^{***}	-1.74^{***}	-0.45	-1.68^{***}
	(0.32)	(0.41)	(0.43)	(0.48)
Parental Educ. (low)	-0.09	-0.34^{*}	-0.23	0.31
	(0.10)	(0.15)	(0.20)	(0.17)
Parental Educ. (lowest)	0.23^{*}	0.16	-0.49^{*}	0.80^{***}
	(0.11)	(0.15)	(0.20)	(0.17)
Year (std.)	0.69^{***}	0.27^{***}	0.85^{***}	0.97^{***}
	(0.06)	(0.07)	(0.09)	(0.09)
Days between tests (std.)	0.37^{***}	0.44^{***}	0.05	0.53^{***}
	(0.06)	(0.07)	(0.09)	(0.09)
R^2	0.03	0.02	0.01	0.03
Adj. R^2	0.02	0.01	0.01	0.03
Num. obs.	358379	352629	272362	343126
RMSE	11.04	14.96	18.87	16.98
N Clusters	937	937	930	936

$^{***}p < 0.001$; $^{**}p < 0.01$; $^{*}p < 0.05$

Table S18. Main effects with sibling fixed effects

	Composite	Maths	Reading	Spelling
Treatment	-3.36^{***}	-3.24^{***}	-2.87^{***}	-3.44^{***}
	(0.24)	(0.32)	(0.39)	(0.36)
Year (std.)	0.75^{***}	0.34^{*}	0.71^{***}	1.20^{***}
	(0.10)	(0.14)	(0.17)	(0.16)
Days between tests (std.)	0.35^{***}	0.39^{**}	0.00	0.64^{***}
	(0.10)	(0.14)	(0.15)	(0.16)
Num. Groups (Family)	18522	18522	18196	18520
R^2	0.22	0.21	0.25	0.24
Adj. R^2	0.04	0.02	0.02	0.05
Num. obs.	97764	96065	77592	92962
RMSE	11.01	15.08	18.79	16.84
N Clusters	731	731	715	730

$^{***}p < 0.001$; $^{**}p < 0.01$; $^{*}p < 0.05$

Table S19. Social inequality with sibling fixed effects

	Composite	Maths	Reading	Spelling
Treatment	-3.25^{***}	-3.14^{***}	-2.85^{***}	-3.25^{***}
	(0.24)	(0.32)	(0.39)	(0.36)
Treat x Par. Educ. (low)	-1.42^{*}	-0.71	-0.61	-2.90^{**}
	(0.63)	(0.89)	(1.08)	(0.97)
Treat x Par. Educ. (lowest)	-1.95^{***}	-2.24^{**}	-0.04	-3.38^{***}
	(0.52)	(0.81)	(0.94)	(0.94)
Parental Educ. (low)	1.07	0.35	0.80	1.33
	(0.67)	(0.87)	(1.52)	(1.24)
Parental Educ. (lowest)	0.95	0.32	0.73	2.11
	(0.82)	(1.19)	(1.68)	(1.41)
Year (std.)	0.75^{***}	0.34^{*}	0.71^{***}	1.20^{***}
	(0.10)	(0.14)	(0.17)	(0.16)
Days between tests (std.)	0.35^{***}	0.39^{**}	0.00	0.64^{***}
	(0.10)	(0.14)	(0.15)	(0.16)
Num. Groups (Family)	18522	18522	18196	18520
R^2	0.22	0.21	0.25	0.24
Adj. R^2	0.04	0.02	0.02	0.05
Num. obs.	97764	96065	77592	92962
RMSE	11.01	15.08	18.79	16.83
N Clusters	731	731	715	730

$^{***}p < 0.001$; $^{**}p < 0.01$; $^{*}p < 0.05$

Table S20. Main effects, OLS in sibling subsample

	Composite	Maths	Reading	Spelling
Treatment	-3.38^{***}	-3.27^{***}	-2.99^{***}	-3.57^{***}
	(0.13)	(0.18)	(0.24)	(0.21)
Year (std.)	0.74^{***}	0.30^{***}	0.75^{***}	1.19^{***}
	(0.06)	(0.08)	(0.11)	(0.09)
Days between tests (std.)	0.44^{***}	0.52^{***}	0.14	0.59^{***}
	(0.04)	(0.05)	(0.07)	(0.06)
(Intercept)	0.84^{***}	0.63^{***}	1.26^{***}	0.79^{***}
	(0.05)	(0.07)	(0.09)	(0.08)
R^2	0.01	0.01	0.00	0.00
Adj. R^2	0.01	0.01	0.00	0.00
Num. obs.	97764	96065	77592	92962

$^{***}p < 0.001$; $^{**}p < 0.01$; $^{*}p < 0.05$

Table S21. Social inequality, OLS in sibling subsample

	Composite	Maths	Reading	Spelling
Treatment	-3.26^{***}	-3.17^{***}	-2.94^{***}	-3.38^{***}
	(0.13)	(0.18)	(0.25)	(0.21)
Treat x Par. Educ. (low)	-1.78^{***}	-0.89	-1.23	-3.28^{***}
	(0.47)	(0.65)	(0.89)	(0.78)
Treat x Par. Educ. (lowest)	-1.91^{***}	-2.25^{***}	-0.36	-2.92^{***}
	(0.43)	(0.59)	(0.82)	(0.72)
Parental Educ. (low)	0.17	-0.36	0.27	0.65
	(0.25)	(0.34)	(0.47)	(0.39)
Parental Educ. (lowest)	0.72^{**}	0.42	-0.57	2.13^{***}
	(0.23)	(0.31)	(0.44)	(0.35)
Year (std.)	0.74^{***}	0.30^{***}	0.75^{***}	1.19^{***}
	(0.06)	(0.08)	(0.11)	(0.09)
Days between tests (std.)	0.44^{***}	0.52^{***}	0.13	0.59^{***}
	(0.04)	(0.05)	(0.07)	(0.06)
(Intercept)	0.81^{***}	0.63^{***}	1.27^{***}	0.70^{***}
	(0.05)	(0.07)	(0.09)	(0.08)
R^2	0.01	0.01	0.00	0.00
Adj. R^2	0.01	0.01	0.00	0.00
Num. obs.	97764	96065	77592	92962

$^{***}p < 0.001$; $^{**}p < 0.01$; $^{*}p < 0.05$

Table S22. Social inequality by student sex

	Composite	Maths	Reading	Spelling
Treatment	-3.05^{***}	-2.93^{***}	-3.24^{***}	-2.90^{***}
	(0.17)	(0.20)	(0.24)	(0.26)
Treat x Female	-0.03	-0.01	0.07	-0.00
	(0.12)	(0.16)	(0.20)	(0.18)
Treat x Par. Educ. (low)	-1.19^{**}	-1.01	-0.38	-2.38^{***}
	(0.40)	(0.54)	(0.61)	(0.61)
Treat x Par. Educ. (lowest)	-1.12^{**}	-2.20^{***}	0.24	-1.48^{*}
	(0.40)	(0.56)	(0.61)	(0.69)
Treat x Par. Educ. (low) x Female	-0.15	-0.19	-1.42	1.24
	(0.55)	(0.69)	(0.94)	(0.83)
Treat x Par. Educ. (lowest) x Female	-0.13	0.78	-1.19	0.36
	(0.45)	(0.61)	(0.79)	(0.90)
Female	0.25^{***}	0.67^{***}	-0.97^{***}	0.70^{***}
	(0.05)	(0.06)	(0.08)	(0.08)
Parental Educ. (low)	-0.36^{*}	-0.33	-0.92^{**}	0.05
	(0.16)	(0.23)	(0.31)	(0.26)
Parental Educ. (lowest)	0.21	0.64^{**}	-1.48^{***}	1.01^{**}
	(0.17)	(0.22)	(0.26)	(0.34)
Year (std.)	0.69^{***}	0.26^{***}	0.86^{***}	0.97^{***}
	(0.06)	(0.07)	(0.09)	(0.09)
Days between tests (std.)	0.48^{***}	0.55^{***}	0.20^{**}	0.65^{***}
	(0.05)	(0.05)	(0.07)	(0.09)
(Intercept)	0.55^{***}	0.23^{**}	1.57^{***}	0.21
	(0.07)	(0.08)	(0.09)	(0.12)
R^2	0.01	0.01	0.00	0.00
Adj. R^2	0.01	0.00	0.00	0.00
Num. obs.	358379	352629	272362	343126
RMSE	11.14	15.02	18.94	17.20
N Clusters	937	937	930	936

$^{***}p < 0.001$; $^{**}p < 0.01$; $^{*}p < 0.05$

Table S23. Social inequality by prior performance

	Composite	Maths	Reading	Spelling
Treatment	-2.96^{***}	-3.15^{***}	-3.02^{***}	-2.45^{***}
	(0.17)	(0.21)	(0.25)	(0.28)
Treat x Par. Educ. (low)	-3.57^{***}	-3.13^{**}	-4.99^{***}	-2.43^{*}
	(0.88)	(1.14)	(1.45)	(1.08)
Treat x Par. Educ. (lowest)	-2.42^{*}	-3.35^{*}	-1.85	-1.91
	(1.05)	(1.32)	(1.62)	(1.49)
Treat x Par. Educ. (low) x Prior Perf. (middle)	1.81	0.79	4.00^{*}	0.85
	(1.08)	(1.33)	(1.73)	(1.44)
Treat x Par. Educ. (lowest) x Prior Perf. (middle)	0.77	-0.45	2.16	1.15
	(1.26)	(1.61)	(1.87)	(1.71)
Treat x Par. Educ. (low) x Prior Perf. (bottom)	3.02^{**}	2.77^{*}	4.66^{**}	1.06
	(0.97)	(1.22)	(1.57)	(1.21)
Treat x Par. Educ. (lowest) x Prior Perf. (bottom)	1.60	2.30	1.48	0.79
	(1.10)	(1.39)	(1.67)	(1.64)
Treat x Prior Perf. (middle)	-0.23	0.14	-0.34	-0.61^{**}
	(0.13)	(0.19)	(0.24)	(0.21)
Treat x Prior Perf. (bottom)	-0.05	0.64^{**}	-0.22	-0.75^{**}
	(0.16)	(0.21)	(0.25)	(0.25)
Parental Educ. (low)	-0.50	-0.86^{*}	-1.16^{*}	0.44
	(0.30)	(0.41)	(0.51)	(0.48)
Parental Educ. (lowest)	0.15	0.31	-1.44	1.12^{*}
	(0.40)	(0.54)	(0.75)	(0.55)
Prior Perf. (middle)	0.54^{***}	0.47^{***}	0.30^{**}	0.79^{***}
	(0.06)	(0.08)	(0.11)	(0.10)
Prior Perf. (bottom)	1.07^{***}	0.90^{***}	0.78^{***}	1.43^{***}
	(0.07)	(0.09)	(0.12)	(0.11)
Year (std.)	0.69^{***}	0.26^{***}	0.86^{***}	0.97^{***}
	(0.06)	(0.07)	(0.09)	(0.09)
Days between tests (std.)	0.49^{***}	0.55^{***}	0.20^{**}	0.66^{***}
	(0.05)	(0.05)	(0.07)	(0.09)
(Intercept)	0.17^{*}	0.12	0.75^{***}	-0.15
	(0.07)	(0.08)	(0.10)	(0.13)
R^2	0.01	0.01	0.00	0.00
Adj. R^2	0.01	0.01	0.00	0.00
Num. obs.	358379	352629	272362	343126
RMSE	11.13	15.02	18.94	17.20
N Clusters	937	937	930	936

$^{***}p < 0.001$; $^{**}p < 0.01$; $^{*}p < 0.05$

Table S24. Main effects with single comparison year

	Composite	Maths	Reading	Spelling
Treatment	-2.51^{***}	-2.94^{***}	-2.39^{***}	-1.98^{***}
	(0.14)	(0.17)	(0.17)	(0.22)
Days between tests (std.)	0.40^{***}	0.57^{***}	0.13	0.47^{***}
	(0.07)	(0.07)	(0.09)	(0.11)
(Intercept)	0.96^{***}	0.78^{***}	1.29^{***}	0.94^{***}
	(0.08)	(0.09)	(0.11)	(0.14)
R^2	0.01	0.01	0.00	0.00
Adj. R^2	0.01	0.01	0.00	0.00
Num. obs.	175419	171396	145929	163322
RMSE	11.18	14.95	18.78	17.05
N Clusters	935	935	923	933

$^{***}p < 0.001$; $^{**}p < 0.01$; $^{*}p < 0.05$

Table S25. Social inequality with single comparison year

	Composite	Maths	Reading	Spelling
Treatment	-2.41^{***}	-2.81^{***}	-2.33^{***}	-1.88^{***}
	(0.14)	(0.17)	(0.17)	(0.22)
Treat x Par. Educ. (low)	-1.28^{***}	-1.40^{**}	-1.06^{*}	-1.53^{**}
	(0.33)	(0.43)	(0.54)	(0.49)
Treat x Par. Educ. (lowest)	-1.18^{***}	-1.88^{***}	-0.55	-1.07^{*}
	(0.34)	(0.48)	(0.46)	(0.54)
Parental Educ. (low)	-0.24	-0.00	-0.69^{*}	-0.04
	(0.19)	(0.25)	(0.31)	(0.31)
Parental Educ. (lowest)	0.21	0.50^{*}	-0.84^{**}	0.70
	(0.20)	(0.26)	(0.29)	(0.38)
Days between tests (std.)	0.40^{***}	0.57^{***}	0.13	0.47^{***}
	(0.07)	(0.07)	(0.09)	(0.11)
(Intercept)	0.96^{***}	0.75^{***}	1.36^{***}	0.91^{***}
	(0.08)	(0.09)	(0.11)	(0.14)
R^2	0.01	0.01	0.00	0.00
Adj. R^2	0.01	0.01	0.00	0.00
Num. obs.	175419	171396	145929	163322
RMSE	11.18	14.95	18.78	17.05
N Clusters	935	935	923	933

$^{***}p < 0.001$; $^{**}p < 0.01$; $^{*}p < 0.05$

Table S26. Main effects excluding testing date

	Composite	Maths	Reading	Spelling
Treatment	-2.78^{***}	-2.61^{***}	-3.12^{***}	-2.46^{***}
	(0.16)	(0.19)	(0.21)	(0.24)
Year (std.)	0.69^{***}	0.26^{***}	0.86^{***}	0.97^{***}
	(0.06)	(0.07)	(0.09)	(0.09)
(Intercept)	0.60^{***}	0.48^{***}	0.99^{***}	0.51^{***}
	(0.07)	(0.07)	(0.08)	(0.12)
R^2	0.00	0.00	0.00	0.00
Adj. R^2	0.00	0.00	0.00	0.00
Num. obs.	358379	352629	272362	343126
RMSE	11.15	15.04	18.95	17.22
N Clusters	937	937	930	936

$^{***}p < 0.001$; $^{**}p < 0.01$; $^{*}p < 0.05$

Table S27. Social inequality excluding testing date

	Composite	Maths	Reading	Spelling
Treatment	-2.68^{***}	-2.50^{***}	-3.06^{***}	-2.35^{***}
	(0.16)	(0.19)	(0.21)	(0.24)
Treat x Par. Educ. (low)	-1.24^{***}	-1.07^{**}	-1.13^{*}	-1.67^{***}
	(0.28)	(0.39)	(0.48)	(0.44)
Treat x Par. Educ. (lowest)	-1.18^{***}	-1.79^{***}	-0.38	-1.27^{*}
	(0.33)	(0.43)	(0.43)	(0.51)
Parental Educ. (low)	-0.27^{*}	-0.31	-0.62^{**}	0.13
	(0.12)	(0.17)	(0.21)	(0.20)
Parental Educ. (lowest)	0.20	0.40^{*}	-1.02^{***}	0.90^{**}
	(0.16)	(0.17)	(0.20)	(0.30)
Year (std.)	0.69^{***}	0.26^{***}	0.86^{***}	0.97^{***}
	(0.06)	(0.07)	(0.09)	(0.09)
(Intercept)	0.60^{***}	0.48^{***}	1.06^{***}	0.46^{***}
	(0.07)	(0.07)	(0.08)	(0.12)
R^2	0.00	0.00	0.00	0.00
Adj. R^2	0.00	0.00	0.00	0.00
Num. obs.	358379	352629	272362	343126
RMSE	11.15	15.04	18.95	17.22
N Clusters	937	937	930	936

$^{***}p < 0.001$; $^{**}p < 0.01$; $^{*}p < 0.05$

References

1. J Scheerens, M Ehren, P Sleegers, R de Leeuw, Country background report for the Netherlands (OECD Review on evaluation and assessment frameworks for improving school outcomes) (2012).
2. J Zapata, et al., *Education Policy Outlook: Netherlands*. (Organisation for Economic Co-operation and Development OECD), (2014).
3. HA Patrinos, School choice in the Netherlands (CESifo DICE Report 2/2011, ifo Institute for Economic Research, Munich) (2011).
4. JM Ritzen, J Van Dommelen, FJ De Vijlder, School finance and school choice in the netherlands. *Econ. Educ. Rev.* **16**, 329–335 (1997).
5. HF Ladd, EB Fiske, Weighted student funding in the Netherlands: A model for the US? *J. Policy Analysis Manag.* **30**, 470–498 (2011).
6. A Schleicher, *PISA 2018: Insights and interpretations*. (OECD Publishing), (2018).
7. M de Haas, R Faber, M Hamersma, How COVID-19 and the Dutch 'intelligent lockdown' changed activities, work and travel behaviour: Evidence from longitudinal data in the Netherlands. *Transp. Res. Interdiscip. Perspectives*, 100150 (2020).
8. ME Kuiper, et al., The intelligent lockdown: Compliance with COVID-19 mitigation measures in the Netherlands (PsyArXiv) (2020).
9. P Tullis, Dutch cooperation made an 'intelligent lockdown' a success. *Bloom. Businessweek* (2020).
10. OECD, *Students, computers and learning: Making the connection*. (OECD Publishing), (2015).
11. Statistics Netherlands (CBS), The Netherlands leads Europe in internet access (https://www.cbs.nl/en-gb/news/2018/05/the-Netherlands-leads-europe-in-internet-access) (2018).
12. G Di Pietro, F Biagi, P Costa, Z Karpinski, J Mazza, *The likely impact of COVID-19 on education: Reflections based on the existing literature and recent international datasets*. (Publications Office of the European Union), (2020).
13. FM Reimers, A Schleicher, *A framework to guide an education response to the COVID-19 Pandemic of 2020*. (OECD Publishing), (2020).
14. SIVON, Opnieuw extra geld voor laptops en tablets voor onderwijs op afstand (https://www.sivon.nl/actueel/opnieuw-extra-geld-voor-laptops-en-tablets-voor-onderwijs-op-afstand/) (2020).
15. E Grewenig, P Lergetporer, K Werner, L Woessmann, L Zierow, COVID-19 and educational inequality: How school closures affect low-and high-achieving students (IZA Discussion Paper 13820, Institute of Labor Economics, Bonn.) (2020).
16. T Bol, Inequality in homeschooling during the corona crisis in the Netherlands: First results from the LISS panel (SocArXiv) (2020).
17. A Andrew, et al., Inequalities in children's experiences of home learning during the COVID-19 lockdown in England. *Fiscal Stud.* **41**, 653–683 (2020).
18. A Bacher-Hicks, J Goodman, C Mulhern, Inequality in household adaptation to schooling shocks: Covid-induced online learning engagement in real time. *J. Public Econ.* **193**, 104345 (2020).
19. C Bansak, M Starr, COVID-19 shocks to education supply: how 200,000 US households dealt with the sudden shift to distance learning. *Rev. Econ. Househ.*, in press (2021).
20. MM Jæger, EH Blaabæk, Inequality in learning opportunities during COVID-19: Evidence from library takeout. *Res. Soc. Stratif. Mobil.* **68**, 100524 (2020).
21. KF Vlug, Because every pupil counts: the success of the pupil monitoring system in the Netherlands. *Educ. Inf. Technol.* **2**, 287–306 (1997).
22. D Fettelaar, E Smeets, Mogelijke indicatoren van schoolgewichten: Onderzoek naar de voorspellende waarde (ITS Institute of Applied Social Sciences, Radboud Universiteit Nijmegen) (2013).
23. G Driessen, De wankele empirische basis van het onderwijsachterstandenbeleid. *Mens en Maatschappij* **90**, 221 (2015).
24. A de Wijs, F Kamphuis, F Kleintjes, M Tomesen, Wetenschappelijke verantwoording: Spelling voor groep 3 tot en met 8 (Cito) (2010).
25. H Feenstra, F Kamphuis, F Kleintjes, R Krom, Wetenschappelijke verantwoording: Begrijpend lezen voor groep 3 tot en met 6 (Cito) (2010).
26. J Janssen, N Verhelst, R Engelen, F Scheltens, Wetenschappelijke verantwoording: Rekenen-wiskunde voor groep 3 tot en met 8 (Cito) (2010).
27. I Jongen, R Krom, M van Onna, N Verhelst, Wetenschappelijke verantwoording van de toetsen technisch lezen voor groep 3 tot en met 5 (Cito) (2011).
28. JK Torgesen, CA Rashotte, RK Wagner, *TOWRE: Test of word reading efficiency*. (Pro-ed Austin, TX), (1999).
29. Ouders van Nu, De drie-minuten-toets (DMT) (https://www.oudersvannu.nl/kind/school/drie-minuten-toets/) (2020).
30. J Jerrim, A Vignoles, Social mobility, regression to the mean and the cognitive development of high ability children from disadvantaged homes. *J. Royal Stat. Soc. Ser. A* **176**, 887–906 (2013).
31. L Borghans, BH Golsteyn, U Zölitz, Parental preferences for primary school characteristics. *The BE J. Econ. Analysis & Policy* **15**, 85–117 (2015).
32. N Ruijs, H Oosterbeek, School choice in Amsterdam: Which schools are chosen when school choice is free? *Educ. Finance Policy* **14**, 1–30 (2019).
33. H Posthumus, et al., Herziening gewichtenregeling primair onderwijs: Fase I (Statistics Netherlands) (2016).
34. H Posthumus, S Scholtus, J Walhout, De nieuwe onderwijs achterstandenindicator primair onderwijs: Samenvattend rapport (Statistics Netherlands) (2019).
35. P Engzell, A Frey, MD Verhagen, Pre-analysis plan for: Learning inequality during the COVID-19 pandemic (Open Science Framework) (2020).
36. JD Angrist, JS Pischke, *Mostly Harmless Econometrics: An empiricist's companion*. (Princeton University Press), (2008).
37. M Carlsson, GB Dahl, B Öckert, DO Rooth, The effect of schooling on cognitive skills. *Rev. Econ. Stat.* **97**, 533–547 (2015).
38. V Lavy, Do differences in schools' instruction time explain international achievement gaps?: Evidence from developed and developing countries. *The Econ. J.* **125**, F397–F424 (2015).
39. PR Rosenbaum, DB Rubin, The central role of the propensity score in observational studies for causal effects. *Biometrika* **70**, 41–55 (1983).
40. GW Imbens, JM Wooldridge, Recent developments in the econometrics of program evaluation. *J. Econ. Lit.* **47**, 5–86 (2009).
41. J Hainmueller, Entropy balancing for causal effects: A multivariate reweighting method to produce balanced samples in observational studies. *Polit. Analysis*, 25–46 (2012).
42. JM Wooldridge, *Econometric analysis of cross section and panel data*. (MIT press), (2010).
43. TA Snijders, RJ Bosker, *Multilevel analysis: An introduction to basic and advanced multilevel modeling*. (Sage), (2011).
44. MA Kraft, Interpreting effect sizes of education interventions. *Educ. Res.* **49**, 241–253 (2020).
45. J Cohen, *Statistical power analysis for the behavioral sciences*. (Academic press), (2013).
46. JP Azevedo, A Hasan, D Goldemberg, SA Iqbal, K Geven, Simulating the potential impacts of COVID-19 school closures on schooling and learning outcomes: A set of global estimates. *World Bank Policy Res. Work. Pap.* (2020).
47. CJ Hill, HS Bloom, AR Black, MW Lipsey, Empirical benchmarks for interpreting effect sizes in research. *Child development perspectives* **2**, 172–177 (2008).
48. J Bound, C Brown, N Mathiowetz, Measurement error in survey data in *Handbook of econometrics*. (Elsevier) Vol. 5, pp. 3705–3843 (2001).
49. P Engzell, JO Jonsson, Estimating social and ethnic inequality in school surveys: Biases from child misreporting and parent nonresponse. *Eur. Sociol. Rev.* **31**, 312–325 (2015).
50. DJ Aigner, Regression with a binary independent variable subject to errors of observation. *J. Econom.* **1**, 49–59 (1973).
51. CR Bollinger, Bounding mean regressions when a binary regressor is mismeasured. *J. Econom.* **73**, 387–399 (1996).

52. ED Looker, Accuracy of proxy reports of parental status characteristics. *Sociol. Educ*. **62**, 257–276 (1989).
53. J Jerrim, J Micklewright, Socio-economic gradients in children's cognitive skills: Are cross-country comparisons robust to who reports family background? *Eur. Sociol. Rev*. **30**, 766–781 (2014).
54. D Black, S Sanders, L Taylor, Measurement of higher education in the census and current population survey. *J. Am. Stat. Assoc*. **98**, 545–554 (2003).
55. A Kapteyn, JY Ypma, Measurement error and misclassification: A comparison of survey and administrative data. *J. Labor Econ*. **25**, 513–551 (2007).
56. JM Abowd, MH Stinson, Estimating measurement error in annual job earnings: A comparison of survey and administrative data. *Rev. Econ. Stat*. **95**, 1451–1467 (2013).
57. C Buchmann, TA DiPrete, The growing female advantage in college completion: The role of family background and academic achievement. *Am. Sociol. Rev*. **71**, 515–541 (2006).
58. DR Entwisle, KL Alexander, LS Olson, Early schooling: The handicap of being poor and male. *Sociol. Educ*. **80**, 114–138 (2007).
59. D Autor, D Figlio, K Karbownik, J Roth, M Wasserman, Family disadvantage and the gender gap in behavioral and educational outcomes. *Am. Econ. Journal: Appl. Econ*. **11**, 338–81 (2019).
60. U Simonsohn, JP Simmons, LD Nelson, Specification curve analysis. *Nat. Hum. Behav*. **4**, 1208–1214 (2020).
